# Inhibition Mechanism of Anti-TB Drug SQ109: Allosteric Inhibition of TMM Translocation of Mycobacterium Tuberculosis MmpL3 Transporter

**DOI:** 10.1021/acs.jcim.3c00616

**Published:** 2023-08-17

**Authors:** Justin Carbone, Nicholas J. Paradis, Lucas Bennet, Mark C. Alesiani, Katherine R. Hausman, Chun Wu

**Affiliations:** Department of Chemistry & Biochemistry, College of Science and Mathematics, Rowan University, Glassboro, New Jersey 08028, United States

## Abstract

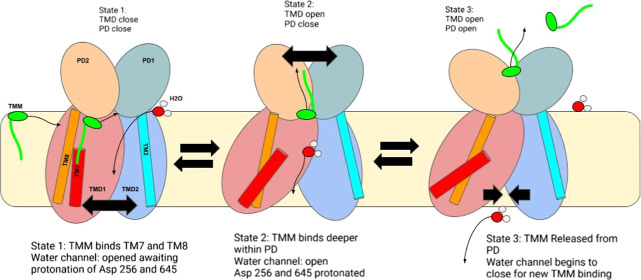

The mycolic acid transporter MmpL3 is driven by proton motive forces (PMF) and functions via an antiport mechanism. Although the crystal structures of the *Mycobacterium smegmatis* MmpL3 transporter alone and in complex with a trehalose monomycolate (TMM) substrate and an antituberculosis drug candidate SQ109 under Phase 2b-3 Clinical Trials are available, no water and no conformational change in MmpL3 were observed in these structures to explain SQ109’s inhibition mechanism of proton and TMM transportation. In this study, molecular dynamics simulations of both apo form and inhibitor-bound MmpL3 in an explicit membrane were used to decipher the inhibition mechanism of SQ109. In the apo system, the close-open motion of the two TM domains, likely driven by the proton translocation, drives the close-open motion of the two PD domains, presumably allowing for TMM translocation. In contrast, in the holo system, the two PD domains are locked in a closed state, and the two TM domains are locked in an off pathway wider open state due to the binding of the inhibitor. Consistent with the close-open motion of the two PD domains, TMM entry size changes in the apo system, likely loading and moving the TMM, but does not vary much in the holo system and probably impair the movement of the TMM. Furthermore, we observed that water molecules passed through the central channel of the MmpL3 transporter to the cytoplasmic side in the apo system but not in the holo system, with a mean passing time of ∼135 ns. Because water wires play an essential role in transporting protons, our findings shed light on the importance of PMF in driving the close-open motion of the two TM domains. Interestingly, the key channel residues involved in water passage display considerable overlap with conserved residues within the MmpL protein family, supporting their critical function role.

## Introduction

Every year, approximately 10 million people across the globe become infected with the bacterial agent that causes tuberculosis (TB), *Mycobacterium tuberculosis* (*Mtb*), leading to approximately 1.5 million deaths in 2020.^1^ In recent years, the Covid-19 pandemic has aided in the decrease in the number of *Mtb* infections through new public health requirements and quarantine, with an unfortunate tradeoff in increased Covid-19-related deaths worldwide.^1^ Due to increasing TB drug resistance for current therapies and the risk of accelerated drug resistance from poor treatment compliance, it is a necessity to develop new drugs that aim at novel targets in *Mtb*.

**Figure 1:**
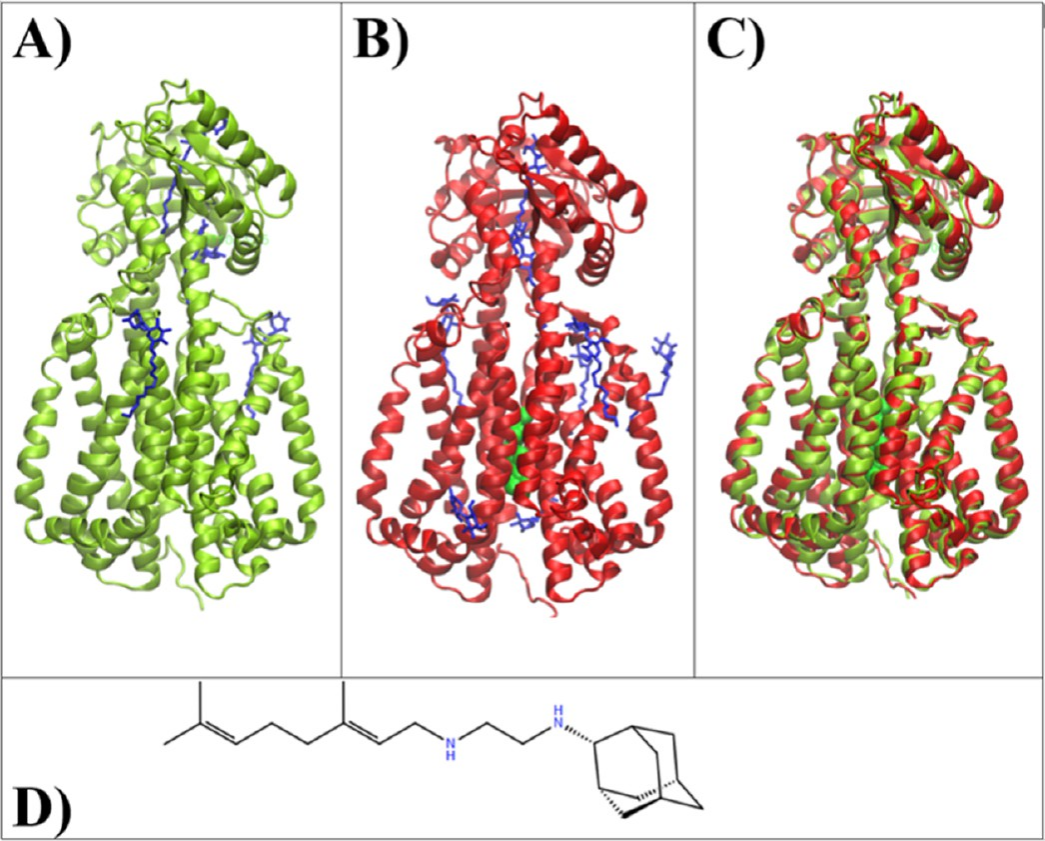
(A) Apo structure of MmpL3 (PDB ID: 6AJF) (yellow) and bound TMM mimic substrates (blue). (B) Holo structure of MmpL3 (PDB ID: 6AJG) (red) with bound inhibitor SQ109 (green) and TMM mimic substrates (blue). (C) Superimposed apo structure (yellow) and holo structure (red) of MmpL3, with global protein rmsd < 2 Å. (D) SQ109 chemical structure.

Mycolic acids are major lipid components of the mycobacterial cell wall and are essential for the survival of *Mtb*.^2−4^ Mycobacterial membrane protein large 3 (MmpL3, Figure 1A) is the key proton-motive-force (PMF)-dependent transporter of trehalose monomycolates (TMMs) across the cell membrane and into the periplasmic space for building the outer cell envelope.^5,6^ MmpL3 is an antiporter protein composed mainly of α-helices and a short β-turn motif (Figure S1), which couples the outbound movement of TMMs with the inbound movement of protons driven by the cross membrane electrostatic potential and the chemical potential gradient (Figure S2).^5^ This mechanism groups MMPL3 as a member of the resistance-nodulation-cell-division (RND) transporters family, which is a protein family of secondary active transporters that share a highly conserved secondary structure using the coupling of proton transport or ATP to transport substrates out of the cytoplasm.^7^ Many RND pumps’ secondary structure includes a transmembrane domain (TMD) with two structural repeats composed of 12 TM helices and porter domain (PD) with two structural repeats. Structural repeats of MMPL3 defined as TMD are composed of TMD1: (TM1-6 residues 1–34, 170–343) and TMD2 (TM7-12 residues 400–420, 548–730), while structural repeats of PD are connected to TMD from TM1–TM2 (residues 35–169) for PD1 and TM7–TM8 (residues 421–547) for PD2. While structurally similar to RND’s, MMPL3 is highly conserved for its protein family, MMPL3 also shares a highly conserved genomic sequence between its orthologs *M. Tuberculosis* and *M. Smegmatis;* MMPL3 orthologs may have a similar functionality. Along with high conservation, insertional inactivation of MmpL3 leads to complete loss of viability of *Mtb*.^8^ Furthermore, inhibition of MmpL3 by small molecule drugs (e.g., EMB and INH) leads to a sharp decline in TMM transport^8^ and bacterial growth both in vitro and in a TB mouse model, suggesting that MmpL3 inhibitors are a promising therapeutic strategy.^9,10^

SQ109 (Figure 1D) is a chemical derivative of ethambutol (EMB) and is currently under Phase 2b-3 Clinical Trials as a promising new treatment of TB. From EMB, SQ109 incorporates an additional bulky head group and unsaturated isoprene units to enhance its anti-TB activity.^11^ Encouragingly, SQ109 has been shown to be a promising inhibitor of MmpL3. Tahlan et al. have shown that SQ109 decreased the incorporation of TMMs into the mycobacterial cell wall by inhibiting TMM transport similarly to EMB and INH.^12^ Consistently, drug-resistant mutant strains produced by EMB and INH were also cross-resistant to SQ109.^8,10,12,13^ Whole-genome sequencing of these mutant strains highlight mutations in the MmpL3 gene, suggesting that SQ109, EMB, and INH act on MmpL3.^13,14^ However, SQ109 decreases cellular mycolic concentrations more than EMB and INH and even accumulates in the pulmonary system.^12^ This accumulation is critical to SQ109’s efficacy, as the pulmonary system is the primary site of *Mtb* infection.^15^

Multiple high-resolution structures for the MmpL3 transporter have been solved. Zhang et al. solved 2.8 Å X-ray crystal structures of the *Mycobacterium smegmatis* MmpL3 in the unbound state (apo-form) (PDB ID: 6AJF) and inhibitor-bound state (holo-form) with several MmpL3 inhibitors including SQ109 (PDB ID: 6AJG) (Figure 1A,B),^16^ sharing high sequence similarity to MmpL3 from *Mtb* (Figure S3). Su et al. solved a 2.8 Å cryo-EM structure of *M. smegmatis* MmpL3 in the TMM-bound state (holo-form) in a 1:2 MmpL3/TMM binding ratio (PDB ID: 7N6B) (Figure S4C–F).^17^ The comparison between the apo-form and two holo-form structures reveals high conformational similarity, suggesting that the conformational changes in crystal MMPL3 are very small; rmsd ∼1.1 Å for 6AJF and 7N6B, while 6AJG and 7N6B showed higher rmsd 1.5 Å (Figures S4A–F, 1C, S4D,E). More importantly, comparison between the apo-form and inhibitor holo-form structures suggests that SQ109 binds inside the structural repeats MmpL3 TMD, where the central channel of the TMD is formed and disrupts two Asp–Tyr pairs (Asp256–Tyr646 and Asp645–Tyr257) located in the center of the TMD, which appear to be key facilitators of proton translocation. Therefore, it has been suggested that SQ109 works by directly blocking the proton translocation pathway and indirectly blocking TMM translocation.^16^ Yet, these structures are not sufficient to explain the dynamic nature of Mmpl3 in its TMM transportation and its inhibition by SQ109.

MMPL3 is a secondary efflux transporter that follows the structure and functionality to the efflux transporter classifications.^18−20^ Efflux transporters commonly couple transport of a substrate in return for an energy generation step. In primary transporters such as ABC (ATP binding cassette superfamily), ATP hydrolysis is utilized for substrate transport.^21^ In secondary transporters such as RND (resistance nodulation division transporters), substrate transport is coupled with an ion gradient or proton motive force (PMF) to utilize biological energy.^21^ MMPL3 is a secondary efflux transporter that utilizes PMF. Proton translocation is conducted through a series of hydrogen bonded networks of water molecules (water wire). The water wire that plays a critical role in conducting protons in bulk water^22,23^ and through the networked water molecules and titratable amino acids by hydrogen bonds in biological channels.^16,24^

RND transporters play a role in antimicrobial drug extrusion as well as cell wall synthesis by transporting key lipid components from the cytoplasm to the outer membrane.^21^ Many multidrug resistant (MDR) bacteria utilize RND for its adaptive multidrug efflux mechanisms to remove antimicrobials from the cell.^21^ Most RND transporters are proposed to function as trimers but recently have found some such as HpnN and MMPL3 can form other oligomeric states such as dimers and monomers (Figure S5).^21^ The tertiary structures of RND transporters are highly conserved in the TM region including 12 TM helices,^25^ as shown in Figure S5. Small conformational changes to the TM helices cause significant changes in the PDs. In RNDs such as multiple transferable CDEs (Mtr), the MtrD inner membrane region utilizes the protonation of central aspartate residues located in the TM proton channel to become protonated and induces conformational changes to allow for entry of substrates to the PD channels MtrD^25^ (Figure S5B). MexB is another RND pump that undergoes key conformational changes in the TMD induced by protonation of central Asp residues^26^ (Figure S5B). HpnN, a hopanoid shuttle, was crystallized as a homodimer with two monomeric subunits that shuttle hopanoids to the out membrane to remodel the cell wall in Gram negative bacteria^21^ (Figure S5A). AcrB is one of the most studied RND transporters, which has the same functionality as MtrD and MexB; they function as a homotrimer to transport cytotoxic substrates in MDR bacteria and have been shown to induce conformational changes based on protonation of 2 Asp residues in the center of the channel between structural repeats of the TMDs^7^ (Figure S5A,B). Small conformational changes induced by this protonation event have very small rmsd values, 3 Å in MexB,^26^ 2 Å in MtrD,^25^ and >0.5 Å rmsd in AcrB.^7^ The O/T/L (open/loose/tight) model proposed by Eicher et al. in 2014 explains the effect of these changes on the functionality of trimeric RND transporters such as AcrB^7^ (Figure S5D). Fairweather et al. (2021) showed through simulations that MtrD central channel residues Asp 405, Asp406, Lys948, and Tyr985 contribute to the conformational changes, allowing opening and closing of drug efflux channels located in the PD based on the engagement of these residues controlled by the protonation of Asp residues and their subsequent interaction with corresponding Lys948 and Tyr985. In AcrB, Asp 407 and Asp 408 initially interact with positively charged Lys 544 residue that acts as an ionization state, intermediately fulfilling the need for the ionic interaction of the deprotonated Asp 407 and Asp 408.^7^ As proton relay occurs through the center of the channel and reaches Asp 407 and Asp 408, protonation causes dissociation from Lys 544 and a repulsion effect, inducing a conformational change in the TM domain. This conformational change is caused by the newly protonated Asp 407 and Asp 408, interacting with flanking helices associated with movement of PD. This in turn opens the channel to the center cavity of PD in AcrB.^7^ While MMPL3 is vastly different from AcrB, MtrD, and other RND transporters, MMPL3 works as a monomer and has no funnel domain; these insights are useful to help identify small changes in the protein structure that allow its transporting mechanism such as protonation and deprotonation events causing opening and closing of the central TMD channel (Figure S5). A sequence and structural comparison of the TMDs of MmpL3, HpnN, and AcrB shows that Asp 256, Asp 344, and Asp 407 are conserved across all three transporters, as shown in Figure S5C. Additionally, the latter fact of no conformational change for MmpL3 makes it harder to explain how TMM translocation is inhibited: after flipping from the inner lipid leaflet layer to the outer lipid layer, TMM must enter and pass through its PD central cavity (PDB ID: 7N6B).^17^ Therefore, a further analysis of the structure dynamics of MMPL3 is needed to determine the significance of small changes in rmsd, causing water efflux and PMF along with its connection to allow for entry and exit of the TMM substrate. Elucidating the water molecule and protein dynamics in the central channel and the two TM domains of MmpL3 is critical for understanding the mechanism of proton transport in MmpL3 and elucidating the conformational changes of MmpL3 is critical to decipher the TMM translocation inhibition mechanism by SQ109. Understanding these mechanisms will allow for further insight into developing novel and effective anti-TB drugs acting on the PMF of MmpL3.^8,27,28^

Molecular dynamics (MD) simulations provide structural and motional properties (i.e., atomic mean-square fluctuations) of a system at atomic-level and femtosecond resolutions, which is complementary to experimental data to decipher molecular mechanisms.^29^ MD simulations have successfully uncovered the proton translocation mechanisms of some proton channels, including influenza M2, gramicidin, and MmpL5, demonstrating that water plays important roles in proton translocation.^30−34^ This work focuses on MmpL3 from *M. smegmatis* over *M. tuberculosis* for a few reasons. First, while the MmpL3 proteins from *M. tuberculosis* and *M. smegmatis* show high sequence homology to each other, only the *M. smegmatis* protein crystal structure has been solved. Second, *M. smegmatis* is non-pathogenic and is much safer to work with compared with *M. tuberculosis*. Finally *M. smegmatis* is considered a viable substitute for *M. tuberculosis*; in vivo studies have shown that MmpL3 orthologs from *M. smegmatis* and *M. tuberculosis* can substitute for each other and replace loss in MmpL3 functionality. In this study, 3 × 1000 ns MD simulations of both the apo-form (PDB ID: 6AJF) and the inhibitor-bound holo-form (PDB ID: 6AJG) of MmpL3 were carried out in explicit water solvent to decipher the MmpL3 proton translocation pathway and inhibition mechanism of SQ109. We observed conformational changes of TM and PDs of both apo and holo systems, but their structural states were very different in comparison. Water molecules were observed to pass through the MmpL3 central channel from the extracellular space to the cytoplasmic space in the apo system with a mean passing time of ∼135 ns. Comparison with the TMM-bound holo-form and inhibitor-bound holo-form suggests an allosteric inhibition mechanism by SQ109 in negatively impacting TMM entry into the extracellular channel for its translocation.

## Materials and Methods

### Protein and Ligand Structure Preparation

**Figure 2:**
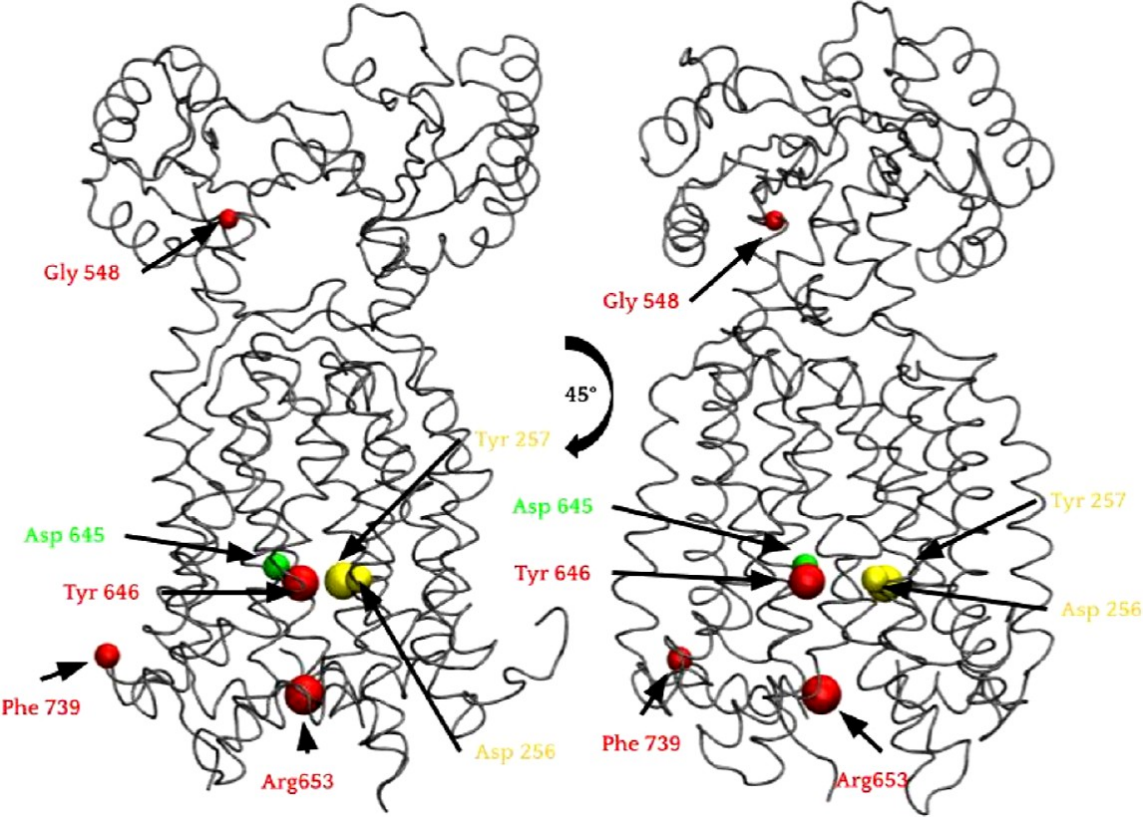
Three-dimensional structure of the MmpL3 sequence with conserved residues displayed with a cutoff at 80% with the protein family. Residues are colored by conservation, 100 (red), 92 (green), 84% (yellow).

The crystal structure of the MmpL3 apo-form (PDB ID: 6AJF) and MmpL3 inhibitor-bound holo-form with SQ109 (PDB ID: 6AJG) were imported from the Protein Data Bank. Included within the structures were multiple detergents and substrate mimic ligands that were removed. As previously stated, no water molecules were in the original structures (Figure 1). The T4 lysozyme (residues 749–929) portion of the protein was removed, as it was necessary only for fusing to the C-terminus during protein crystallization to prevent degradation.^27^ A homology model was constructed based on the *M. smegmatis* MmpL3 FASTA sequence (UniProt ID: I7G2R2)^35^ using Prime software of Schrodinger Suites 2018 to ensure the structure sequence is correct (Figure 2 and S6–S8).

The SQ109 crystal ligand bond order was corrected, and empirical p*K*_a_ calculation was performed at pH 7 using Epik software of Schrodinger Suites 2018 to generate correct ionization states.^36^ The lowest charge state for the crystal ligand was chosen for geometry minimization to relax the atoms for a best-fit structure. The merged protein–ligand complex was then prepared using the Protein Preparation Wizard in Maestro of Schrodinger Suites 2018. Protein preprocessing was performed on the apo- and holo-forms to assign correct bond orders, insert hydrogen atoms and disulfide bonds, and remove water beyond 5 Å from heteroatomic groups. Prior to preprocessing, missing protein loops and side chains were also generated using Prime. Visual comparison between the initial crystal structure and the Prime structure was done to validate structure correctness. Optimization of the complex charge state was completed using PROPKA at pH 7. Restrained minimization was then performed to relax the protein using the OPLS3e force field.^37^

### MmpL Protein Family Alignment

The MmpL protein family (Pfam ID: PF03176) alignment was obtained from the Pfam database. The seed file contained approximately 25 different members of the MmpL family used as representative sequences for the entire family (approximately 29,000 members). Multiple sequence alignment of these sequences against the *M. smegmatis* MmpL3 protein sequence was done using Jalview (Figure S9). Residues with ≥50% sequence identity were tabulated and mapped to the MmpL3 structure (Table S1, Figures 2 and S9B,C).

### Channel Analysis

To understand the possible pathways water may take through MmpL3, the channel was analyzed using MOLEOnline channel analysis calculation.^38^ First, the protein was imported to maestro in apo-form (PDB ID: 6AJF) and holo-form (PD BID: 6AJG) and prepared to fill in missing side chains and loops using Prime. All ligands (except SQ109 in the holo-form) were discarded in the apo- and holo-forms. The prepared protein was imported to VMD to remove the T4 lysozyme (residues 749–929). The protein was then submitted to MOLEOnline pore analysis where all surfaces, cavities, and voids were selected with a probe radius of 13 Å and an interior threshold of 0.8 Å with beta structure, membrane region, and selections turned off. The channel was selected, and key residues were analyzed for bottlenecks (Figures S10–S13). Bottlenecks were determined using the channel properties diagram with the lowest radius.

### MD Simulation System Setup

The prepared protein apo- and holo-form structures were submitted to the OPM server^39^ to place the protein in the correct membrane orientation. Structures were then prepared for MD simulations. Both structures were surrounded by a POPC (300 K) lipid membrane model^40^ and then solvated in a SPC^41^ water box with a buffer distance of 10 Å. A salt concentration of 0.15 M NaCl was added, and additional Na^+^ ions were added to neutralize the negative system charge. All systems were built with an OPLS2005^42^ force field using the Desmond System Builder.

### Equilibration Phase

Using the Desmond module, the systems were relaxed using the eight-step default relaxation protocol for membrane proteins.^43−45^ First, solute heavy atoms were minimized without restraints and then repeated with restraints. The systems were equilibrated by slowly increasing the temperature from 0 to 300 K, followed by a water barrier and gradual restraining. The *NPT* ensemble was then simulated with a constant number of particles, constant pressure (1 bar), and constant temperature (300 K) with a water barrier and restraints on heavy atoms. The systems were then further simulated under *NPT* conditions with additional equilibrations of both lipids and solvents. Simulations under *NPT* conditions were performed with heavy atoms annealing from 10 to 2 kcal/mol and then with Cα atoms retrained at 2 kcal/mol. Finally, simulations under *NPT* conditions were done with no restraints for 1.2 ns.

### Production Run

Three separate production runs were performed for each complex under the *NPT* ensemble using the default protocol for 1000 ns. The detailed simulation system information is within the Supporting Information (Table S3). Using M-SHAKE,^46^ bonds with hydrogen atoms were constrained, allowing for a 2.0 fs time-step within the simulations. Long-range electrostatic interactions were analyzed using the *k*-space Gaussian plot Ewald method,^47^ while using a charge grid spacing of ∼1.0 Å and a direct sum tolerance of 10^–9^. Short-range non-bonding interactions had a cutoff of 9 Å, and long-range van der Waals interactions were based on an approximate uniform density. An r-RESPA integrator^48^ was used to condense the computation and calculate non-bonding forces. Short-range forces were updated every 2 fs, and the long-range forces were updated every 6 fs. The trajectories were saved every 50 fs for analysis. A pressure of 1 bar was controlled by the Martyna–Tobias–Klein chain coupling scheme (coupling constant = 2 ps), and the temperature of 300 K was controlled by the Nosé–Hoover chain coupling scheme (coupling constant = 1 ps).^49^

### Convergence of Simulations

Convergence of the MD simulations was ensured by analyzing the protein Cα and ligand heavy atom root-mean-square deviation (rmsd) plots for each trajectory. For each complex, steady–state equilibrium was reached when the plots become relatively flat and stable (Figures S18–S20), which suggests that the simulation time of 1000 ns was sufficient to reliably investigate the protein–ligand interactions for the three systems.

### Simulation Interaction Diagram Analysis

**Figure 3:**
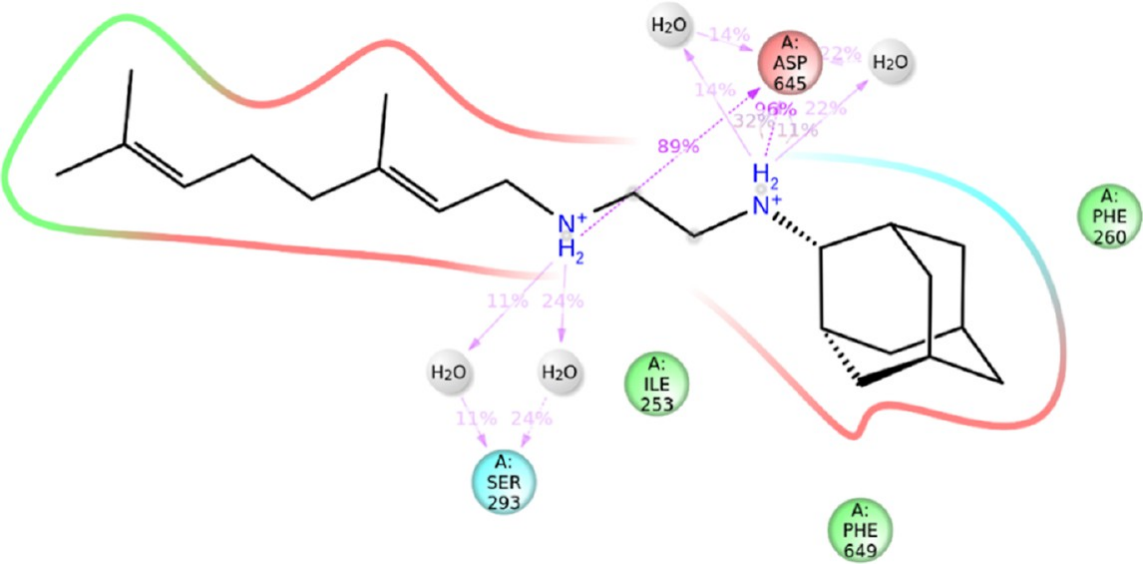
Detailed 2-D schematic of interactions between ligand and protein residues from the combined holo trajectory. Interactions that occur more than 10.0% of the simulation time in the combined trajectory are shown.

**Figure 4:**
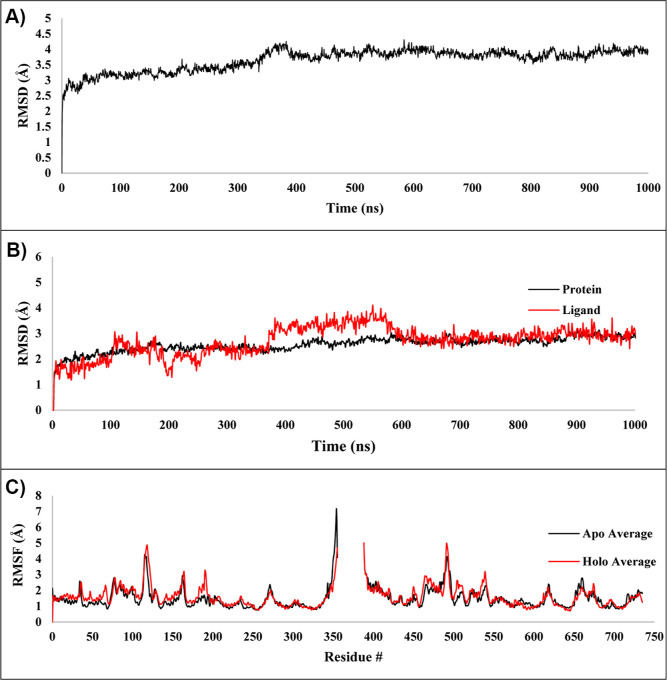
Average rmsd for the three MD simulations of (A) apo-form protein MmpL3 crystal structure (PDB ID: 6AJF) and (B) protein and ligand for holo-form MmpL3 complex crystal structure (PDB ID: 6AJG). (C) Average protein RMSF for the three MD simulations of the apo-form MmpL3 crystal structure (PDB ID: 6AJF) and the holo-form MmpL3 complex crystal structure (PDB ID: 6AJG).

**Figure 5:**
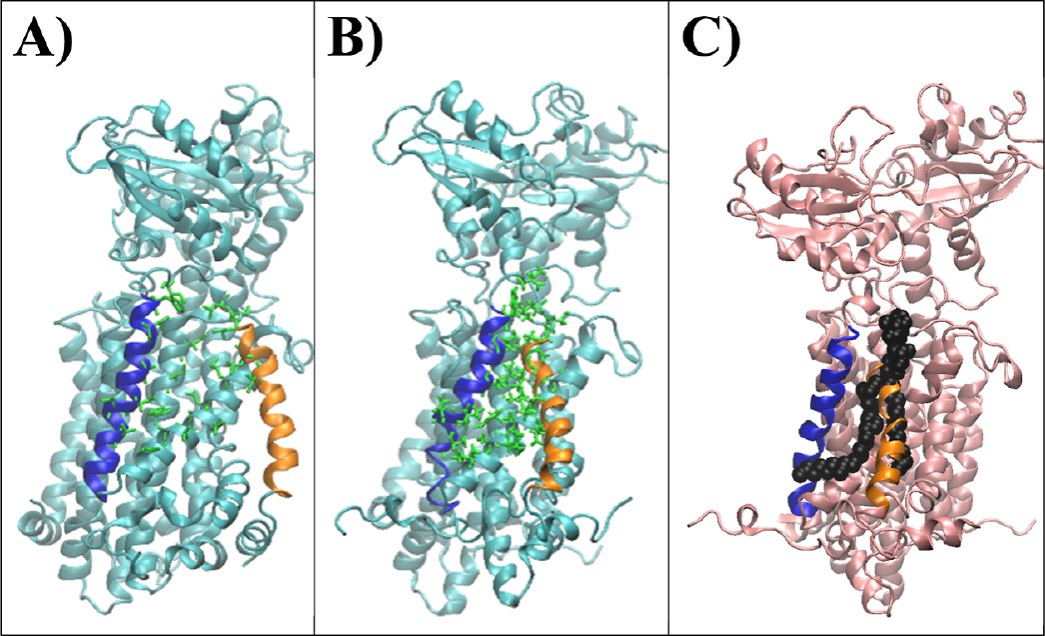
Conformation of TM7 (orange) and TM8 (blue) in MmpL3. Representative (A) apo and (B) holo forms from our MD simulations. (C) Holo form of our MD structure with crystal ligand TMM from PDB ID 7N6B (black). TMM was obtained by superimposing our holo MD structure with the solved MmpL3–TMM crystal complex (PDB ID: 7N6B). Residues interacting with TMM are represented as green licorice sticks (See Figures S27–S29).

Using Maestro, the Desmond simulation interaction diagram (SID) tool was used to analyze the apo- and holo-forms of MmpL3 throughout each MD trajectory (1000 ns) and the combined trajectory (3000 ns). This revealed the protein rmsd (Figure S16), protein secondary structure elements (Figures S18–S20), ligand interaction diagram (Figure 3), and protein and ligand root-mean-square fluctuation (RMSF) (Figure 4C). Dynamics of TM helices 7 and 8 revealed through SID in (Figure 5).

### Normal Mode Analysis

**Figure 6:**
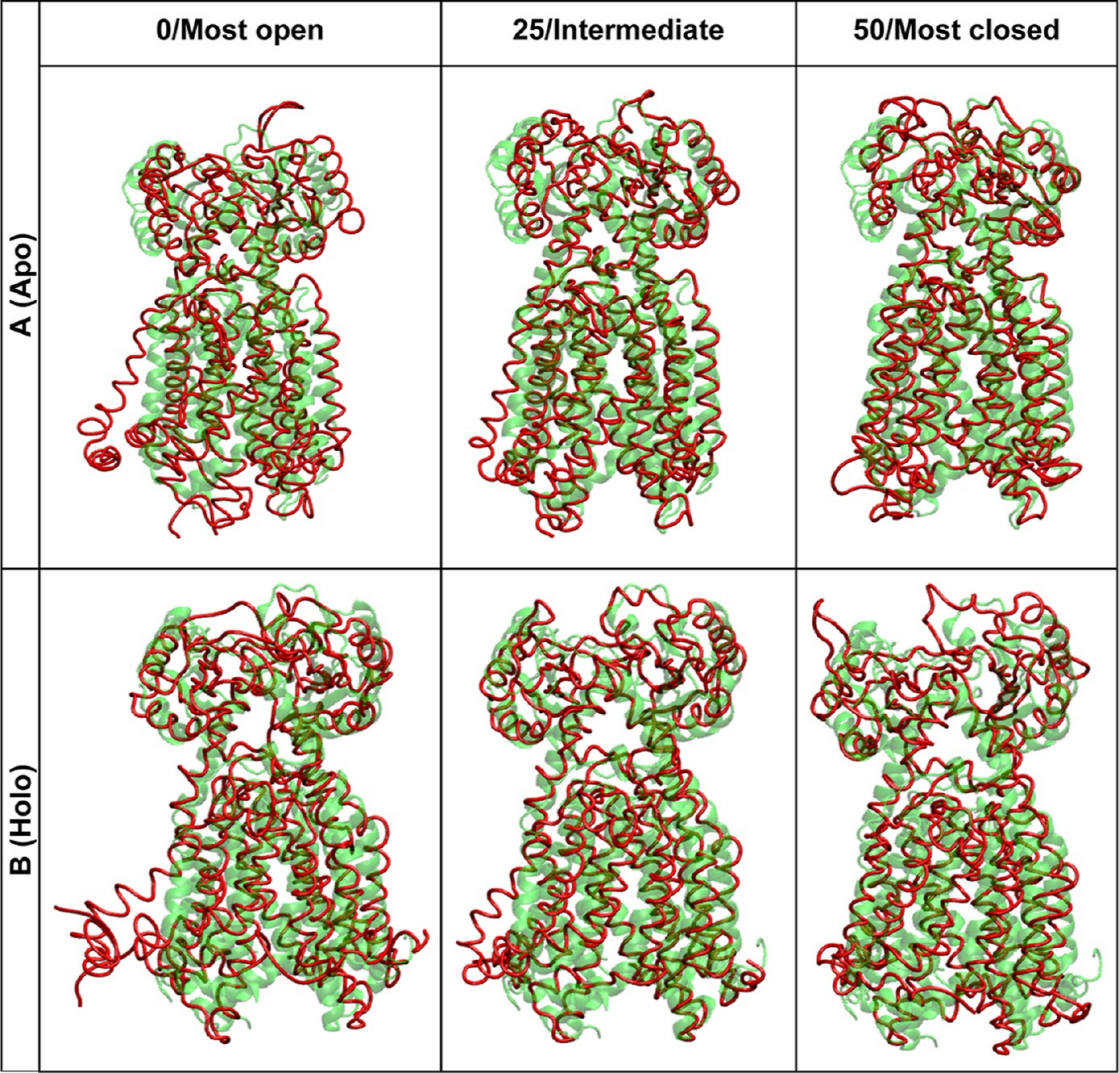
Key conformations of normal mode #3 for Apo system (A) and Holo system (red) (B) compared to the reference crystal structures apo 6AJF and holo 6AJG (green).

The top 10 normal modes of each system (Apo or Holo) were obtained using VMD Normal Mode Wizard (ProDy): 716 CA atoms were selected; principle component analysis calculation was selected; and the combined trajectory (3000 frames of 3000 ns; frame/ns) was used. The picture of each mode is shown in Figure S21. The movies of the selected mode 3 for Apo and mode 5 are included in Supporting Information. In these modes, the clear coupling between the open–close motion of the two TM domains and the open–close motion of the two PD are observed (Figure 6).

### Free Energy Landscape

**Figure 7:**
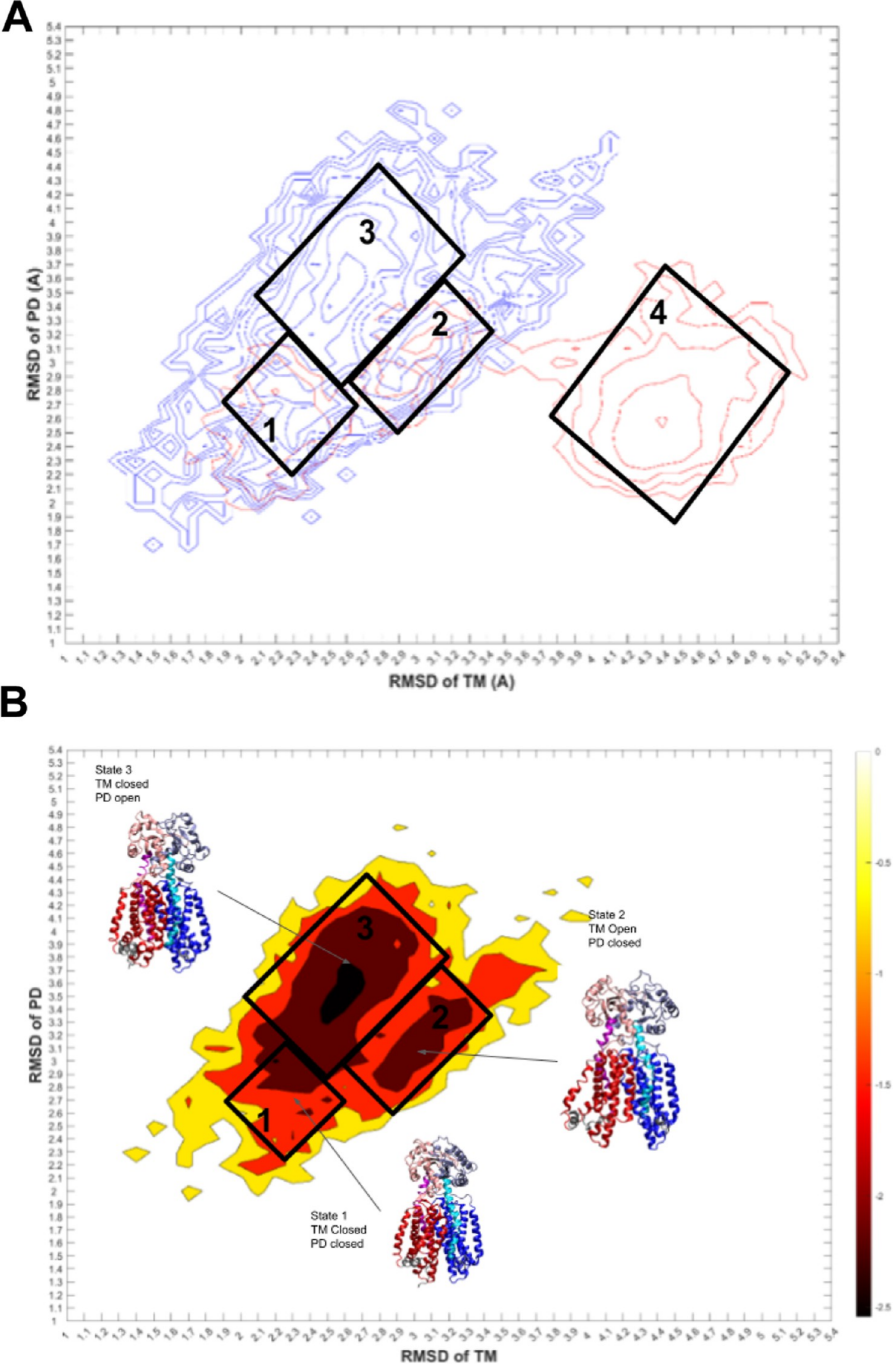

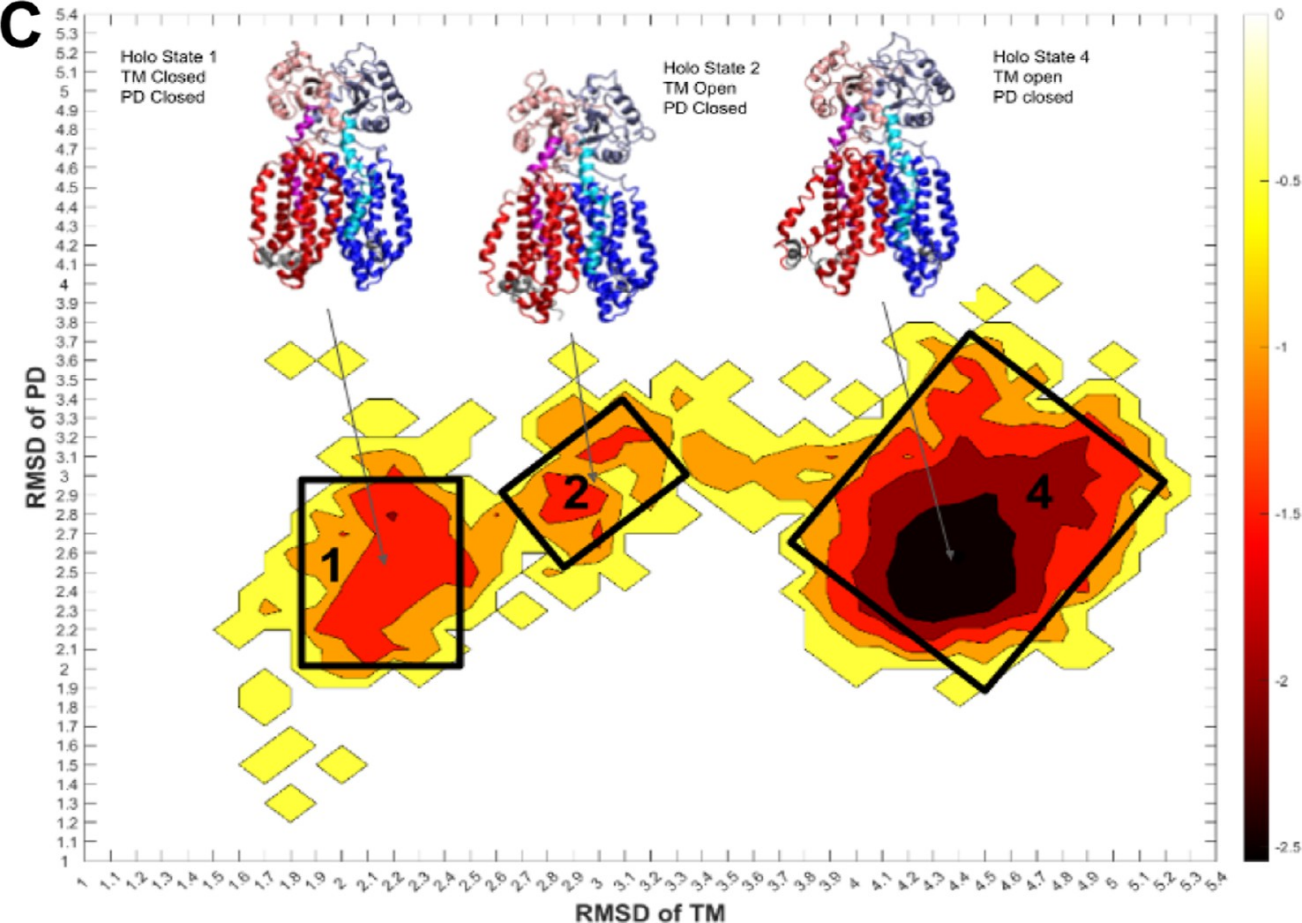
Free energy landscape (unit: kcal/mol) of the combined apo and holo system (A), the apo system (B), and the holo system (C) of Mmpl3. State 1: closed two TM domains and closed two PD domains. State 2: opened two TM domains and closed two PD domains. State 3: closed two TM domains and opened two PD domains. State 4: opened two TM domains and closed two PD domains. A representative structure for each state of the two systems is selected as closest to the middle of the free energy landscape rmsd: state 1 (frame 2337), state 2 (frame 1872), and state 3 (frame 397) for the Apo system; state 1 (frame 295), state 2 (frame 414), and state 4 (frame 648) for the Holo system. Structure coloring: TMD1 residues (1–34, 206–344) blue TM2 (170–205) cyan, PD1 (35–169) iceblue, TMD2 (400–420, 585–730) red, TM8 residues (548–584) magenta, PD2 residues (421–547) salmon.

While the Ca rmsd of the two TMD T1 TM helices 1–6 (residues 10–34, 170–342) and T2 TM helices 7–12 (residues 405–420, 548–584) was calculated to monitor the change in rmsd and association to conformational free energy of these two domains, the Ca rmsd values of the two PDs (the PDs PN/residues 35–169) and PC/residues (437–547) were calculated to monitor the close-open motion of these two PDs (Figures 7 and S22). These two order parameters are used to characterize the conformational coupling between the TMDs and the PDs. Six structures were selected, three from the Apo system and three from the holo system. Selections were based on the closest to lowest free energy within the rmsd range of PD and TMD, as shown in Figure S24. Three states from each system were then pairwise compared within the three system states, as shown in Figure S25A,C for the apo system and Figure S25B,D for pairwise comparison of cross system states.

### Channel Analysis for Free Energy States

**Figure 8:**
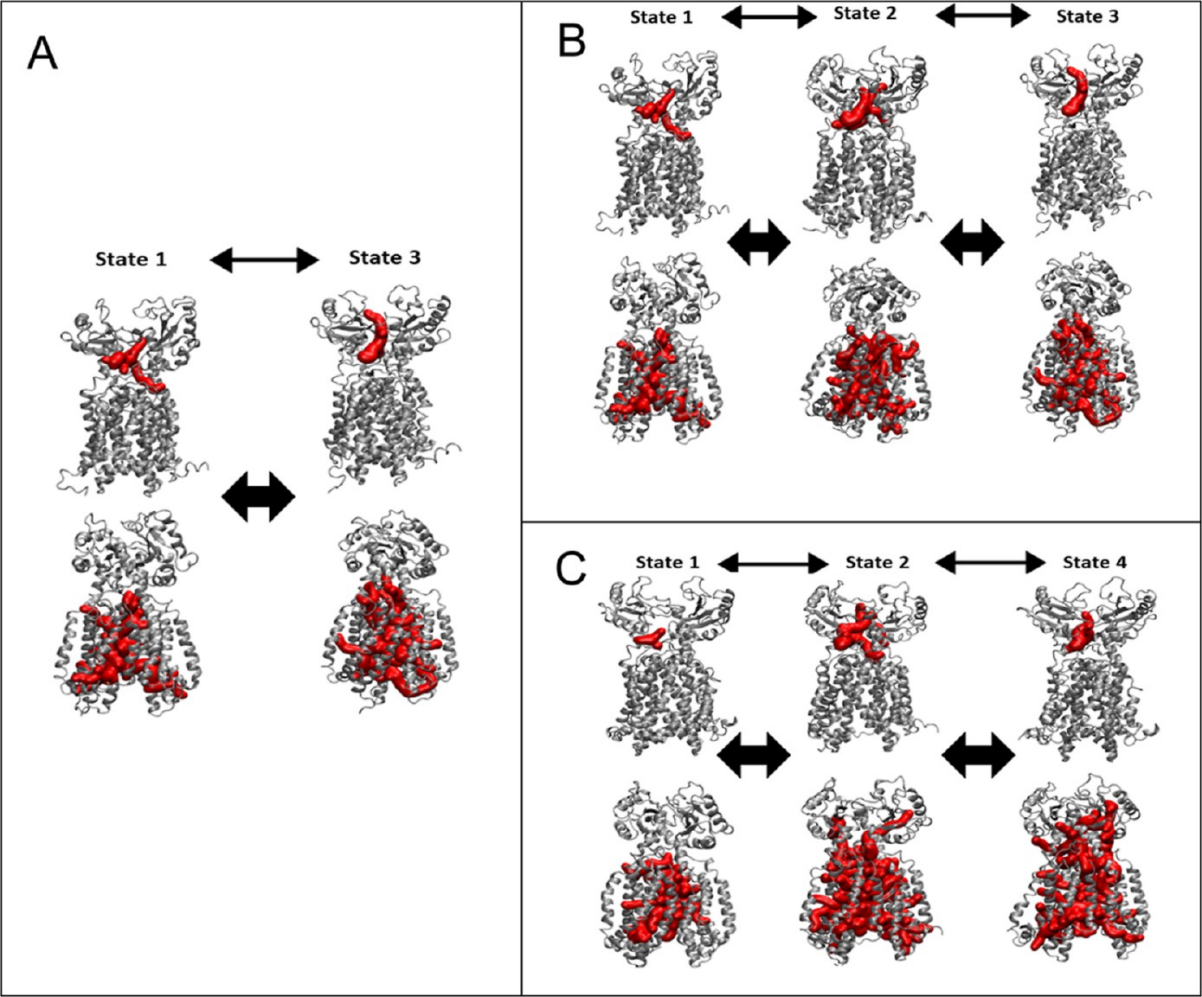
Two parallel pathways (A,B) of the apo system leading to the opened state of the two PD domains and off-pathway (C) of the holo system leading to the closed state of the two PD domains. The structure is rotated to show two viewpoints (Top: the two PDs’ motion; bottom: the two TMDs’ motion). Caver analysis is used to calculate channels of each state. Structures of states are shown in gray, and calculated channels are shown in red. Additionally, see Figure S26 for channel comparisons of Apo and Holo of all states.

To identify key structural changes between four conformational states, each state snapshot was loaded into Pymol for Caver 3.0 Channel analysis. TM domain parameters were consistent for both systems starting the calculation at the center of Asp 256 and 645 and Tyr 257 and 646 core. Next, the parameters of shell depth 3 Å and shell radius 2 Å and a minimum probe size of 0.6 were used in case water was within the channel during the snapshot timeframe. Max distance of the channel was set to 10 Å to cover the length of the protein and the desired radius was set to 1.9 Å Figures 8 and S26. For the PD channel analysis, the radius was decreased by 1 Å because the structures are solid-state, so a larger radius caused low channel detection. Next the TMM entry channel was depicted as the starting point at N524 and G426 with the same parameters of shell depth, radius, and probe radius.

### Water Pathway Analysis

**Figure 9:**
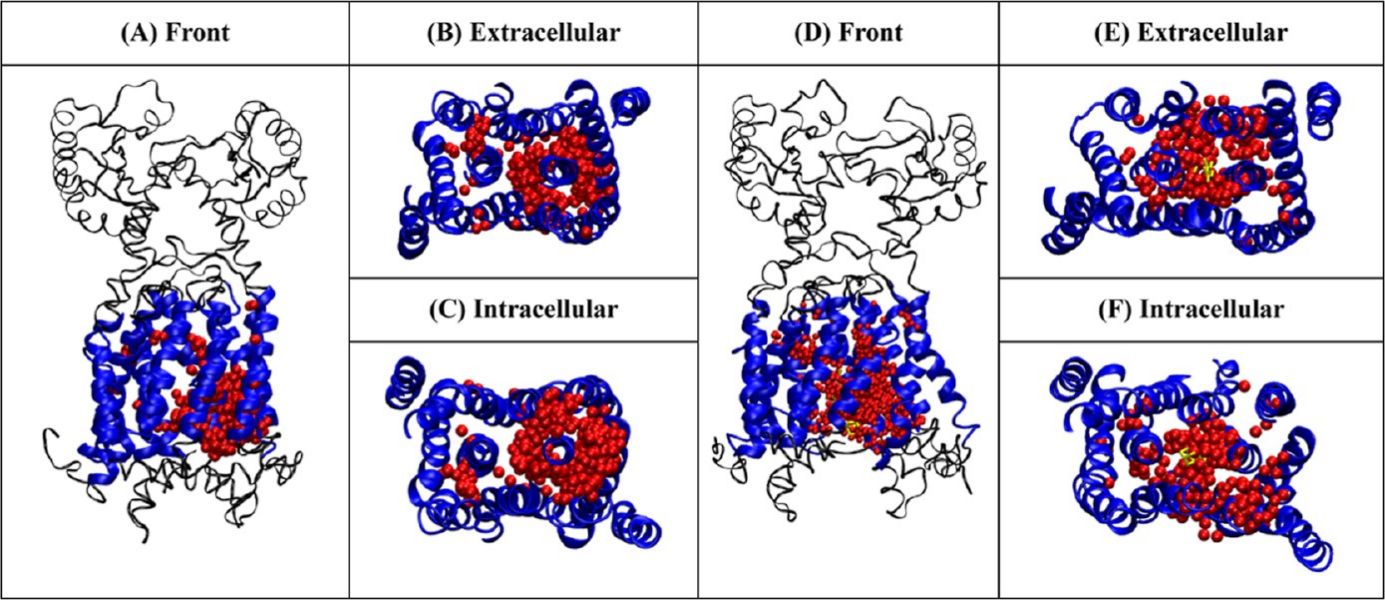
Distribution of all water molecules within the TM throughout entire trajectories of the apo from (A–C) and the holo form (D–F).

Each MD simulation was rigorously analyzed to observe water molecules pass through the TM portion of MmpL3 (Figures 9 and S32 and S34). Water molecules serve as carriers for protons and are likely the mechanism by which the PMF operates. After identifying specific water molecules passing through the protein channel (Table S4 and Figure S34), the VMD hydrogen bond extension tool was used to analyze all hydrogen bonds that formed between identified water molecules and MmpL3 channel residues throughout the trajectory, using a donor–acceptor distance of 4.0 Å, an angle cutoff of 40°, and occupancy of each residue that made a hydrogen bond with the water molecules (Tables S5–S9). Sample occupancy was calculated using the trajectory time for each respective water molecule from the time of entrance to the point of exit. The entrance of a water molecule is defined by the first hydrogen bond that occurs between the water molecule and the protein. The exit of a water molecule is defined as the last hydrogen bond that occurs between the water molecule and the protein. Each binding residue was then annotated for its frequency in binding to water molecules that passed through MmpL3.

## Results

### Protein Family Sequence Alignment Identified MmpL3 Conserved Residues within the Protein Family

The MmpL protein family was aligned against the *M. smegmatis* MmpL3 sequence to identify residues conserved in the MmpL family. By identifying conserved amino acids between *M. Smegmatis* and *M. Tuberculosis*, we were able to identify key residues that led to determining functionality of MMPL3. We considered residues to be conserved within MmpL3 that has at least a percentage sequence identity of 50%. Table S1 displays the identities of these conserved residues, which are visualized in Figures 2 and S14. Increasing the conservation cutoff restriction from 50 to 80% allows for a more refined view. It can be seen here that the most conserved residues are located toward the cytoplasmic portion of the protein. Tyr646 was among the highest of conserved residues (100%), along with Arg653, Gly548, and Pro739. These four residues appeared in every member of this protein family alignment. Asp645 had slightly lower conservation (92%), followed by Asp256 and Tyr257 (84%).

To further analyze the structure of MMPL3, we identified structural repeats within the monomeric MMPL3 crystal structure PDB: 6AJF and identified the RND-like secondary structural elements. The prepared crystal structure was split into two molecules, residues 1–344 for the first structural repeat and residues 388–730 for the second structural repeat. These two molecules were then aligned by the secondary structure and pairwise aligned by sequence yielding a 48% similarity, 23% identity, and 53% conservation. Then, TM domains and PD domains were aligned separately from the secondary structure and then pairwise sequence aligned to show the similarities within these domains, as shown in Figures S14–S6. Interestingly, the TM domain has a similar secondary structure but has a low sequence identity at 15%, but the sequence similarity and conservation both yielded 39 and 52%, as shown in Figures S15C and S16C. On the other hand, the PD shows a highly conserved secondary structure but the sequence when pairwise aligned yielded a 9% ID, 28% similarity, and 29% conservation, as shown in Figures S15B and S16B. Although the secondary structure between structural repeats is very similar, the sequences are vastly different, which may portray the difference in functionality. Interestingly, PD1 includes eight more residues than PD2 which may be the cause for some of the restricted conformational movement observed from normal mode analysis, as shown in Movies 1 and 2.

### Channel Analysis Predicts Key Gating Residues which Regulate Water Passage through MmpL3

The channel of MmpL3 was calculated and analyzed using MOLEonline^38^ to observe the pathway, in which water travels through the protein and its interactions with channel residues. Analysis of TM domain showed that while Asp256–Tyr646 and Tyr257–Asp645 are in close interaction, the Phe260 and Phe649 bottleneck is at 0 Å (Figures S12B and S13B). In this state, the TMD channel is proposed to be in the closed state, not allowing for water translocation due to the bottleneck at Phe260 and Phe649. Apo-form analysis also showed that the calculated channel does not interact with Asp256 and Tyr257 like it did in the holo-form (Table S2). Being also highly conserved among mycobacterial sequences, these residues are important for SQ109 binding. However, they may only serve as simple donor and acceptor groups of protons for a key gating residue, such as Phe649, and are thus more important in overall proton translocation. Protonation of these Asp645 seems to be a key residue in the channel, while protonation may induce conformational changes that will open the bottlenecks and allow for water transport through the TMD channel. These bottlenecks may have increased functionality in conformational change activity since it has been previously reported in proton channels for gating residues to commence conformational change.^31,50^

### rmsd Values in Apo-form and Holo-form MmpL3 Show Subtle Differences between the Two Systems

The average rmsd values for the three apo-form and holo-form simulations can be found in Figure 4, while the rmsd of the individual trajectories can be found in Figure S17. The average apo-form rmsd values were generally consistent throughout the simulation (3–4 Å). There was a slight conformational adjustment at ∼400 ns, after which the protein rmsd assumed steady-state equilibrium. The initial conformational adjustment of the holo-form average rmsd occurred over the first 400 ns. At 600 ns, the MmpL3–SQ109 complex had assumed a stable conformation. To understand further the dynamics of the structure, rmsd was calculated for each structural repeat: TMD 1 residues 1–34 and 170–344, PD1 residues 35–169, TMD2 residues 400–421 and 548–730. A-helices residues 345–399 and residues connecting T4 lysozyme 730–748 were not included in this calculation since they were unfinished and cut from the structure. rmsd of these structural repeats were then plotted over the time of the three trajectories of Apo (Figure S22A) and Holo (Figure S22B).

TMD1 and PD1 in the Apo system are stable throughout all three trajectories, while in Holo, TMD1 and PD1 experience fluctuations at ∼250, 1400, and 2650 ns. rmsd for structural repeats TMD2 and PD2 in both apo and holo systems show large fluctuations through the course of the three trajectories. rmsd of TMD1 and TMD2 show stabilization throughout all three trajectories in apo and holo systems, while PD1 and PD2 in the holo system show large fluctuations at 250, 800, and 2600. From this, we conclude that TMD2 and PD2 exhibit the most conformational changes in the apo system but remain significantly more stable in the Holo system. TMD1 and PD1 remain relatively stable in apo and holo systems.

### Receptor RMSF Data Show Slight Differences in Holo-form Receptor Flexibility upon SQ109 Interaction that May Impact TMM Binding and Translocation

The average protein RMSF for the apo- and holo-form simulations can be found in Figure 4C Expectedly, rigid components of the receptor (i.e., some TM helices) exhibit lower RMSF values, while more flexible regions (i.e., N- and C-termini) exhibit higher RMSF values. Comparing the two protein structures, the holo-form structure displays slightly more fluctuations than the apo-form structure. This is most likely due to the ligand binding and the breaking of the hydrogen bond between Asp256 and Tyr646 and between Asp645 and Tyr 257, causing the respective TM helices to separate. Surprisingly, conformational shifting of TM helix 7 (TM7) toward TM helix 8 (TM8) was observed in the holo-form structure after ∼400 ns, narrowing the region between TM7 and TM8 (Figure 5). After ∼600 ns, this conformation was maintained throughout the remainder of the trajectory. This was not observed in the apo-form structure. Interestingly, the region above where TM7–TM8 becomes narrow is the periplasmic central cavity involved in TMM translocation, as previously indicated by Su et al.^17^ To investigate if SQ109 may indirectly block TMM translocation, we superimposed our holo-form MD structure with the MmpL3–TMM crystal structure (PDB ID: 7N6B)^17^ and compared the conformations of TM7 and TM8. Representative last snapshots of our apo- and holo-form structures (Figure 5) and from each trajectory (Figures S27 and S28) were generated. 7N6B shows the TM7–TM8 region to be wide enough to accommodate TMM binding (Figure S29). TMM seemingly cannot fit within the narrowed TM7–TM8 region of our MD holo-form structure, suggesting that SQ109 might act as an allosteric inhibitor by inducing conformational changes in MmpL3 that likely disrupts TMM translocation.

### SQ109 Binding Disrupts Hydrogen Bonding within MmpL3

Upon SQ109 binding to MmpL3, the hydrogen bond occupied by Asp645 and Tyr257 is broken and the two amine groups within the inhibitor form a new hydrogen bond with Asp645. This interaction occurred 89% of the time and 96% of the time throughout the entire combined holo-form trajectory. Most notably in the Apo simulations, trajectories 1 and 3 show the most events of Tyr646 opening the channel and re-formation of Tyr646 and Asp256 bond. Trajectory 2 only shows this occurrence once throughout the entire trajectory. While in holo form simulations, the protein never returns to the closed state. All trajectories showed that Tyr646 would flip away from Asp256 and SQ109 and move to an intermediate phase to interact with SQ109 but not enough to close the channel and induce conformational change in the PD. In addition, these amines also formed hydrogen bonds with water molecules that have entered and surrounded this region. The known hydrogen bonding between Asp256 and Tyr646 and between Asp645 and Tyr257 is broken in the holo-form, causing the open conformational change in the TM helices.

### Normal Mode Analysis

To decompose the complex conformation change into simple normal modes, normal mode analysis was carried as described in the method section and top 10 modes for each system are presented in the Supporting Information (Figure S21). The best normal mode was selected to represent the open and closing dynamics of MMPL3 as all 10 normal modes presented this movement and normal mode 3 presented the clearest dynamic movement in both systems. Both systems begin at a relatively comparable formation as seen in the reference crystal structure (6AJF and 6AJG) in the most closed state and then move to an intermediate state and lastly to the most open state. In the Apo system, as shown in Figures 6, S21A, and Movie 1, normal mode 3 MMPL3 shows the TM domain opening and closing, specifically TM7 and TM8 causing the PD to close and open. This movement worked as expected where the channel has free movement to open and close based on protonation of the proton channel. In the Holo system (Figures 6, S21B, and Movie 2), normal mode 3 presented a locked conformation of MMPL3. All four sub domains move together, with the most prominent change being in the TM domain where it is locked in the open state and never recloses as it did in the Apo system. Snapshots of normal mode can be seen in Figure 6 compared to the prepared crystal structure of Apo MMPL3 PDB: 6AJF and for all normal mode systems in Figure S21. Compared to the prepared crystal structure of Apo form 6AJF, the structure dynamically moves with the desired movement of TMD and PD. These data support the free energy landscape with the lack of an open PD state in the holo system as the structure dynamically moves together rather than PD2 separating from PD1, as it does in the Apo system.

### Free Energy Landscape and Implied Kinetic Pathways

Free energy landscape for the apo and holo system were obtained using the two-order parameters characterizing the conformational change (rmsd) of the PD and the TM domain (Figure 7). Four lowest free energy states (state 1–4) were identified: state 1–3 for the apo system; states 1–2 and 4 for the holo system (Figure 7A). For the apo system, state 1 is defined as rmsd PD: 2.5–2.9 Å and rmsd TM: 2.0–2.6 Å, state 2 as rmsd PD: 2.9–3.4 Å and rmsd TM: 2.9–3.6 Å, and state 3 as rmsd PD: 3.2–3.9 Å and rmsd TM: 2.4–2.9 Å (Figure 7B). For the Holo system, the state 1 is defined as rmsd PD: 2.2–2.9 Å and rmsd TM: 2.0–2.3 Å, state 2 is determined as an intermediate between state 1 and 4 and was closely reflected to apo state 2 at rmsd TM: 2.7–3.3 and rmsd PD: 2.5–3.3, and lastly state 4 was the most prominent state with the lowest free energy state with rmsd PD: 2.3–3.2 Å and rmsd TM: 4.1–4 (Figure 7C). We observed that a larger PD rmsd state like in the apo system state 3 was absent for the holo system (Figures 7, S22, and S23). This observation agrees with our theory that SQ109 is inhibiting TMM transport allosterically through causing the TM domain to remain in an open state and a PD closed state.

The free energy landscape also allows us to identify a kinetic pathway for each system. In the Apo system, there are two possible parallel routes in which states may move where state 1 and state 3 fluctuate by inversely opening and closing simultaneously or possible second scenario, where state 1 conformationally shifts to state 2 and then to state 3 (Figure 7A,B). The 3 Apo states all have distinct conformations and only fluctuate within 2.0 Å rmsd. The key components of fluctuations are primarily TM 7 and 8 of TMD2 and PD2, as TMD1 and PD1 do not have many conformational fluctuations and stable through the three trajectories. This movement of TM opening and PD closing occurs with the conformational change associated with PD2, TM7, and TM8 and fluctuates inversely, as we see that there is no case in which the structure is energetically stable, and TM and PD are both within an open conformation. Each state is defined in Figure S23 for the Apo and Holo system and compared in Figure S25. The comparison of each state is shown in Figures 7 and S24; three-way pairwise comparisons can show the difference in the open–close state switching in MMPL3 (Figure S25). In the Apo system (Figure S25A), state one presents a TM7 closely intact with the rest of TM domain, while the PD domain is closely related with a small rmsd, pointing to the closed structure. The key difference lies in the comparison with state 2 and state 3, where PD shows an opened and flattened conformation in state 3 as opposed to state 2. This goes in compliance with what was previously reported by Su et al. (2019); they stated that PD conformational movement occurs as PD2 shifts away from PD1.^17,51^ Within these open states, it is also observed that Tyr646 and Asp245 are not interacting via hydrogen bonding from our channel analysis of the MD systems. This agrees with the results that were observed from the holo form simulation where the TM domain is consistently within the open state, as Asp257 and Tyr646 are not interacting. This interaction could play a key role in the closing of the TM domain, which drives the opening of the PD domain, allowing for TMM entry and exit. In the holo system, one pathway is identified: state 1 conformationally shifts to state 2 and then to state 4. All three Holo states (state 1, 2, and 4) show a very similar structure, with the differences lying in the TM domain where the structure is close to a closed state but is blocked through SQ109 blocking Tyr646 and Asp257 interaction, as shown in Figure S25. While the TM domain is in the open state, the PD domain is in the closed state, and as the TM domain closes, the PD domain opens for entry and exit. In the Apo system, state 1 is defined as a closed–closed state and has a much higher free energy than the other states. This state is in close relation with state 1 of the holo system (Figure S25D) where both states show a very similar closed conformation. Apo state 2 is a conformation closed–open state where PD domain has small rmsd and closed entry and exit sites, while the holo state two represents a similar rmsd; the structures do exhibit differences in the TM7 and TM8 location, along with a subtle closing of the TMM entry channel. Holo state 4 is not observed in the apo system but was compared to with states 2 and 3 of the apo system to show the key differences in the TM and PD domains and how within the Holo state 4 the PD domain remain closed (Figure S25D). The structures and their conformational states comparison now present many structural changes of MMPL3 where the connected conformational changes of the TM domain opening and closing cause the inversely closing and opening of PD, while in the Holo system, the absence of an open PD state may be caused by the TM domain unable to rejoin its Asp257 and Tyr646 interaction due toSQ109 binding.

### Channel Analysis on the Free Energy States

Conformation states of Apo and Holo systems from the free energy landscape were then subjected to channel analysis in Pymol’s extension Caver 3.0 (Figures 8 and S26). It was observed that throughout MMPL3’s state conformational changes, the overall channel volume and bottleneck size changed subjected to the state MMPL3 was in. Both domains of MMPL3 were tested separately due to different entry points. The Apo system presented an inverse relation to increase volume of TMD, which led to a decrease in PD. The average bottleneck radii of PD were 1.3, 1.03, and 2.39 Å in states 1, 2, and 3, respectively. In the TMD analysis, volume and bottlenecks changed inversely to the PD domain, and the radii were 0.70, 0.73, and 0.71 Å in states 1, 2, and 3, respectively. The Holo system channel analysis presented an overall decrease in PD volume and bottleneck radius, while TMD presented an increase from states 1, 2, and 4. The bottleneck radius of PD presented radii of 1.58, 1.00, and 0.94 Å, and the TMD showed an increase from 0.74, 0.79, and 0.85 Å in states 1, 2, and 4, respectively. Here, we observed dynamically connected inverse movement of PD and TMD and the effects of SQ109 on the channel volume and bottleneck radius in PD domain showing that SQ109 greatly affects the PD transport channel while being bound deep within TMD.

### Water Passage Analysis Revealed Distinct Binding Pathways through MmpL3

Analyzing the differences in the distribution of all water molecules within the TM channel for apo- and holo-form simulations reveals slight differences (Figures 9, S32, and S33). The apo-form allowed for a more organized, clustered distribution of water molecules, while the holo-form caused a slight dispersion of water molecules within the TM region. It is important to note that all observed water molecules in the holo-form simulations entered and exited through the cytoplasmic portion of the protein; in the apo-form simulations, observed water molecules entered MmpL3 through the periplasmic portion and exited through the cytoplasmic portion. Therefore, it is much less likely for a water molecule to pass through the protein when bound to an inhibitor.

**Figure 10:**
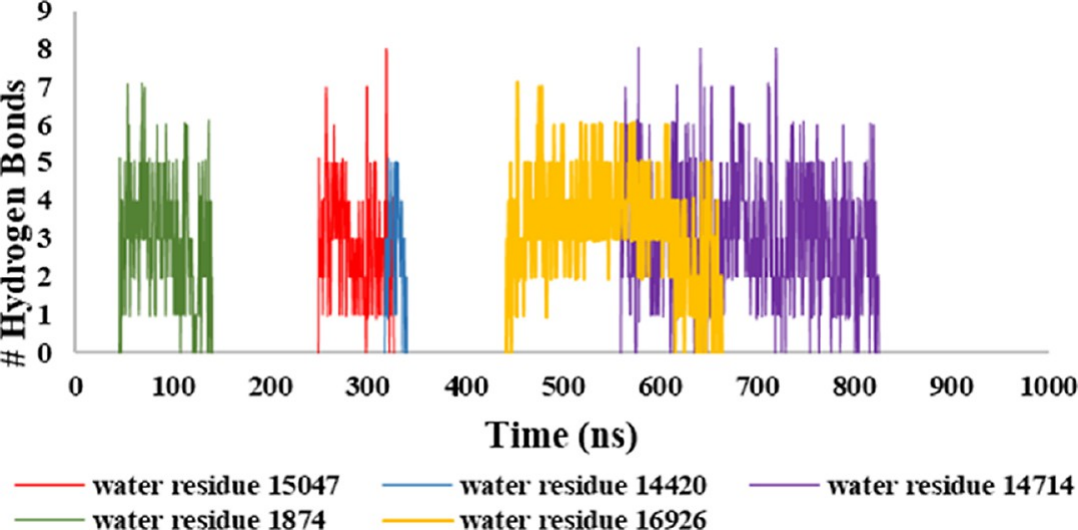
Simulation time scale displaying the time during which each water molecule passes through the apo form MmpL3. See Table S2.

**Figure 11:**
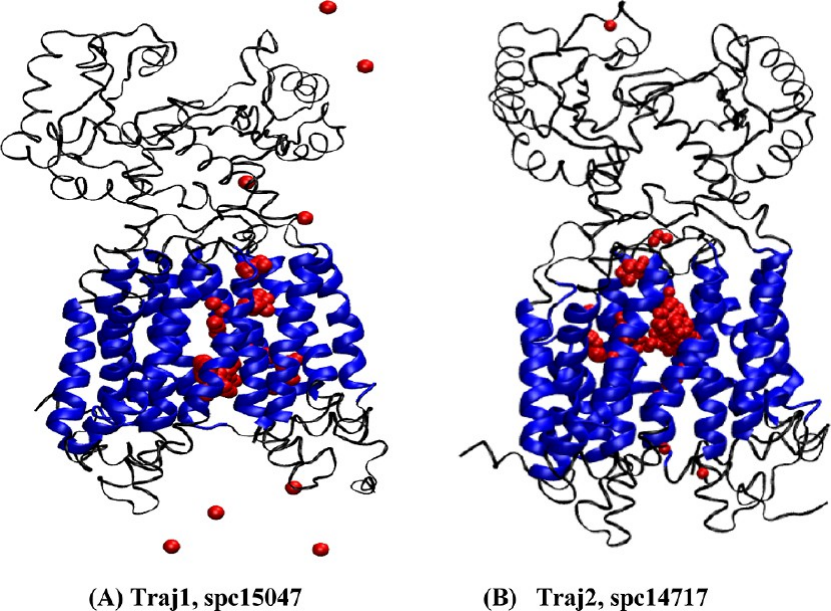
MmpL3 structures of two representative unique water pathways passing through the extracellular space into the TM, and out into the intracellular space throughout the MD apo form simulations. (A,B) Simulations 1 and 2, respectively.

**Figure 12:**
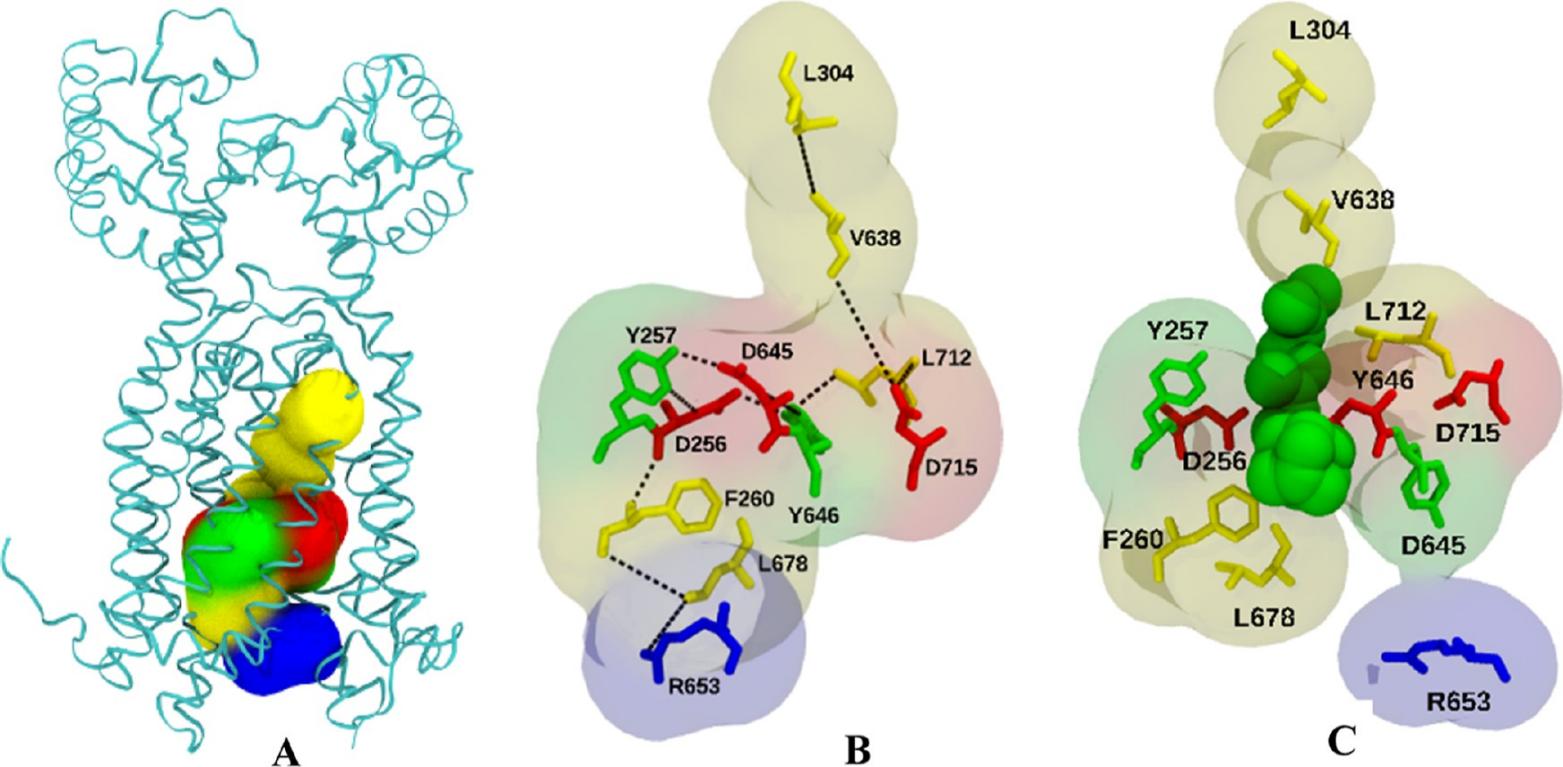
Proposed refined water molecule pathway based on MmpL protein family conserved residues and the most occurring hydrogen-bonding residues throughout the five water passage events. The front view is displayed in (A) and the pathway details in (B,C). Residues are colored by acidic (red), basic (blue), polar (green), and non-polar (yellow).

Five representative water molecules within the apo-form simulations were chosen to illustrate the movement of a proton through MmpL3. The time details for these events can be found in Figure 10, and the distribution of each water molecule within the protein throughout its passing event is displayed in Figure S34. Clearly, there were little similarities relating to the time it takes a water molecule to pass through the channel. Interestingly, the distribution of each water molecule reveals different pathways. Three of five water molecules (Figure S34A,B,E) displayed an elongated path, binding to residues that were considerably spread out within MmpL3. The other two water molecules (Figure S34C,D) displayed a more aggregated path, binding to residues closer together within MmpL3. Figure S34A (water 15047) represents the “elongated” path, while Figure S34C (water 14714) represents the “aggregated” path. Figures 11 and 12 display detailed information for the two water binding pathways (15047 and 14714), including point of entry, point of highest occupancy, and point of exit. Table S4 provides detailed information of each residue that bonded to each water molecule in time order throughout the trajectories. Four of the five chosen water molecules made their first interaction with MmpL3 in a similar residue location. Water molecule 15047 first made contact with MmpL3 at residues Phe307 and Pro630 at ∼250 ns into simulation 1 (Figure 11A,B). Similarly, water molecule 14420 made its first interaction with MmpL3 at Ser301 (Figure S37A–C), only 6 residues away from Phe307. Water molecule 16926 initially bound to Phe307 (Figure S39A–C). Water molecule 14714 was first bound to MmpL3 at residues Gly310 and Lys313 (Figure S39A,B), in close proximity to the previously mentioned residues. These four water molecules entered MmpL3 by diffusing directly into the TM space. The remaining water molecule, 1874, entered MmpL3 by passing through the top funnel located on the periplasmic portion, rather than directly into the TM space (Figure S40A–C). Most residues within the periplasmic portions of MmpL3 are acidic or basic, and it is likely that a water molecule could enter through this area by engaging in hydrogen bonding or electrostatic interactions with these residues.

Each water molecule made its way through MmpL3, staying in contact with different residues for different lengths of time. Hydrogen bond occupancy represents the fraction of conformations within a given set of conformations. Residues binding the representative water molecules with the highest occupancies in each trajectory are displayed in Table S4. Sample occupancy represents the amount of time a residue was bound to the water molecule over the course of a water passing event through MmpL3, whereas population occupancy (Tables S5–S9) represents the amount of time a residue is bound to a water molecule over the course of the entire simulation. Residues exhibiting higher occupancies could be functionally important in the passage of water molecules. Ser300 and Ser301 displayed the highest occupancies in four out of five water passing events and are near the water pathway entrance, determined to be near the Phe307 region.

Water-binding residues were also analyzed for their frequency in these five water-passing events. This was done to narrow down the residues that most likely participate in proton translocation. These residues were all found to be within the space and many of them comprise the SQ109 inhibitor binding pocket (Figure 9D–F). Regardless of the specific path of the water molecule, all five waters made their way to the Asp256, Tyr257, Asp645, and Tyr646 region before exiting the protein. These four residues displayed relatively strong binding with the water molecules. Tables S6–S10 display all the residues that formed a hydrogen bond with the water molecules, ordered by highest occupancy.

### MmpL3 Residues that were Subsequently Conserved throughout the MmpL Family and Participated in Water Passage Events Reveal a Final Pathway

Considering MmpL3 residues that exhibit ≥50% sequence identity, and residues that participated in all five, four, or three water passage events, a positive overlay of these residues is revealed. When mapped to the protein (Figure 12), it was observed that these residues are not only part of the binding pocket, but also form a distinct pathway starting from the periplasmic portion and ending toward the cytoplasmic portion of MmpL3. In this newly proposed pathway, the PMF occurs when a water molecule enters MmpL3 at the Leu304 region, passing down to Val638, Asp715, and Leu712 and then binding with high occupancy to Tyr646, and to a lesser extent Asp256, Tyr257, and Asp645. Phe260 acts a hydrophobic gate for water and small molecules, with water passing only after the phenyl ring adjusts to an “open position”. Finally, it passes to Leu678 and exits out from Arg653.

## Discussion

MmpL3 is a secondary PMF-dependent antiporter that couples proton influx with TMM outflux in *Mtb* cells for mycobacterial cell wall synthesis.^52^ A reduced bacterial load in mouse lungs infected with *Mtb* MmpL3-knockdown mutants demonstrated MmpL3 as essential for *Mtb* replication and viability,^9^ making it a good drug target for treating TB. SQ109 is a potent MmpL3 inhibitor that is under clinical investigation for TB treatment, with previously unsolved mechanism of inhibition and transportation. SQ109 binds to the central channel of MMPL3, between structural repeats in the TMD where most water molecules and Hydrogen bonds were calculated to allosterically inhibit MMPL3. It was previously believed that SQ109 binds within the proton translocation channel of MmpL3 and does not competitively inhibit TMM binding, flipping, and transportation.^27^ It presumably disrupts proton translocation and subsequently the PMF required for TMM translocation.^17^ Therefore, with our study, we introduce the free energy landscape and kinetic pathway of Mmpl3 transportation mechanism along with the way SQ109 locks the protein conformation within one state through understanding the dynamics of MMPL3.

Proton translocation was first described using Grotthuss’ mechanism in 1806, where hydronium ions “hop” between a network of hydrogen-bonded water molecules.^24,53^ Later, Nagle and Morowitz proposed a “proton wire” model, where protons translocate between water and hydrophilic amino acids in a membrane-embedded proton transporter.^54^ Recently, Paulino et al. used ^17^O NMR spectroscopy and DFT-treated MD simulations to characterize the strong binding interactions between a water wire and amino acids in gramicidin A, indicating the significance of water wires in other biological channels.^55^ The ability of water and titratable amino acids to accept and donate a proton to quicken proton translocation, rather than by self-diffusion, is seen in both bulk water and within biological channels. Diffusion and translation coefficient rates have been determined using both experimental and computational methods (Table S11, see references attached). Within bulk water, proton translocation (9.3 × 10^–5^ cm^2^/s) is ∼4-fold faster than water diffusion (2.3 × 10^–5^ cm^2^/s) and ∼7-fold faster than Na^+^ translocation (1.33 × 10^–5^ cm^2^/s). In gramicidin, proton translocation (3 × 10^–5^ cm^2^/s) is the fastest of any known biological transporter. The proton translocation rate has not yet been experimentally determined for MmpL3 or MmpL5; however, MD simulations have calculated water translocation occurring in MmpL3 (∼135 ns, this study) and in MmpL5 (∼20 ns), which are significantly slower than water translocation in influenza M2 (1–2 ns) or gramicidin (∼0.2 ns). Although quantum MD can theoretically calculate proton translocation rates through these channels, the computational cost is quite high. However, we expect proton translocation in these channels to be appreciably faster than water translocation, based on the differences in their experimentally determined rates.

The apo- and SQ109-bound crystal structures of MmpL3 have been determined to investigate SQ109’s inhibition mechanism.^27^ The comparison between the two structures shows that SQ109 binding disrupts hydrogen-bonding interactions between Asp257–Tyr646 and Tyr256–Asp645 pairs, preventing a conformational state change in the TMD that might contribute to TMM binding and translocation.

**Figure 13:**
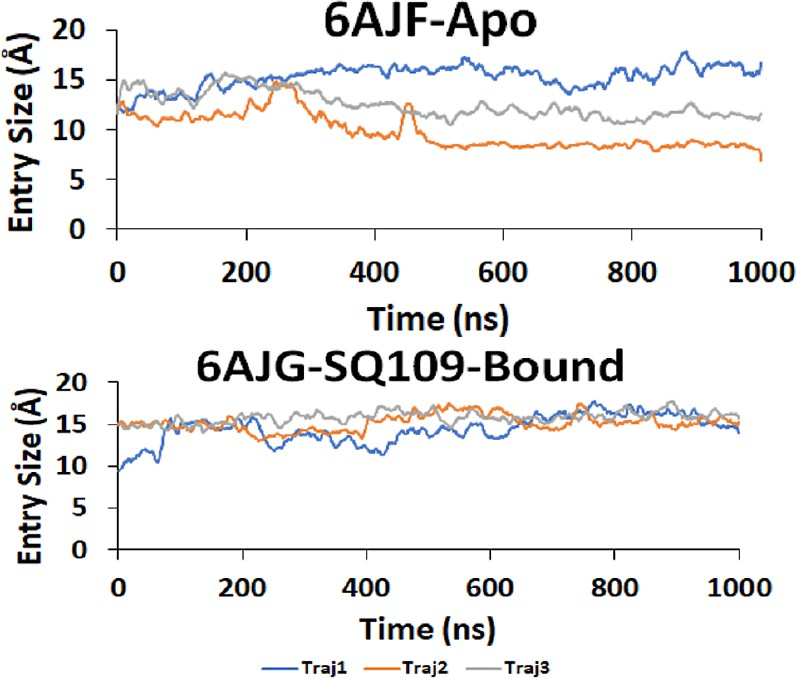
TMM entry site size formed by the distance between gating residues S423 and Asn524 in the apo-form (top) and SQ109-bound (bottom) MmpL3 structures over each trajectory. Channel is considered open at ∼8 Å. See Figure S29.

We believe that this interaction is key in MMPL3’s translocation mechanism due to the apo free energy landscape shown in Figure 7 and normal mode analysis shown in Figure 5, which indicates linked movement of TMD and PD, whereas water hydrates the channel and protonates polar residues such as Asp257 and Tyr646 moving away and toward flanking helices TM7 and TM8. This conformational change closes the entry channel for TMM at residues Ser 423 and Asn524 from 10.2 to 7.73 Å in the apo state 2 (Figures 13 and S30). This channel closing also causes the PD to have a bigger central cavity volume than while the entry and exit channels are closed, as shown in Figure S25,for Apo state 2. The binding of the TMM substrate must occur before the central water channel is protonated and entry of TMM occurs upon protonation of Asp257 and opening of the TM domain. Then for release of TMM from the central cavity of the PD, the central water channel residues are deprotonated due to the water channel entry channel, closing possibly due to TMM binding to the central cavity of PD. These two sites are possibly in the same location. Due to PMF being cut from periplasmic space, the channel pumps water out, causing deprotonation of the channel. Thus, the channel will close, and the PD will once again open, allowing for the exit of a TMM substrate.

**Figure 14:**
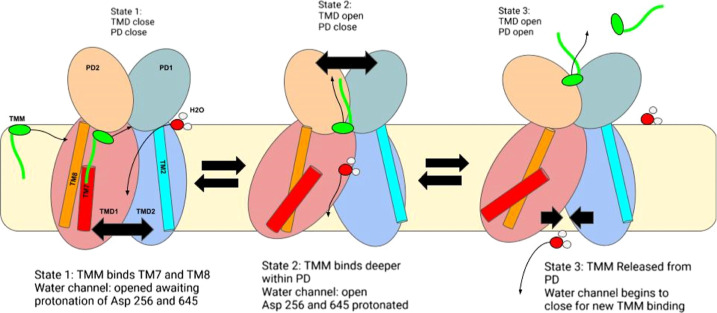
Putative mechanism of the MMPL3 antiporter. The close-open motion of the two TM domains driven by the proton translocation drives the close-open motion of the two PD domains, allowing for TMM translocation. Water enters the channel, while the TMM substrate (green) binds to the TMM binding site between TM8 (red) and TM7 (orange). Water fills the central channel of TM1 and TM2, causing protonation of polar residues lining the channel and causing TMD2 to move away from TMD1. This conformational change caused the closing of the TMM entry channel, which led the TMM substrate to translocate deeper between PD1–PD2, as seen in state 2. As water begins to empty out of the water channel, polar residues holding the channel open will return to normal interactions beginning to close the channel where the PD will open, allowing for TMM exit and re-entry.

SQ109 presumably blocks TMM translocation through filling the central water cavity and interacting exclusively with Asp257 and Asp645, impairing the close-open motion of the two TM domains and thus the PDs to inactivate MmpL3. Through our analysis, we observed that whenever the TMD was within an open state, the PD exhibited its smallest rmsd values. However, the apo-and holo-form crystal structures do not include water molecules that are believed to be critical in water wire-mediated proton translocation; thus inhibition of the PMF can only be inferred here. The absence of structural waters may be due to the use of glycerol, which likely suppresses ice formation that damage the protein crystal structure.^56^ Interestingly, our MD simulations showed water entering MMPL3 and wetting the channel, creating a potential water wire that may participate in proton translocation in both apo and holo systems, raising further questions involving water movement within MMPL3. We also identified interactions between five specific water molecules and key residues that seem to be crucial for proton translocation. From this, Figure 14 displays our newly proposed mechanism: a proton, in the form of water, enters MmpL3 at the top of the TMD at deprotonated polar residues before passing down the center of the TMDs structural repeats and exiting into the cytoplasm (Figure 14).

Proton translocation remains to be the driving force of MMPL3 and its conformational states where central residues Asp 256 and Asp 645 are proton acceptors interacting with the opposing Tyr 257 and Tyr 646. Upon protonation of Asp 256 and Asp 645, we observed the TM domain change conformation into an open state. In the open state, it was also observed that the channel in which TMM passes through had the smallest entry and exit sites (Figures 5 an S27–S30). This entry site interaction with TMM was believed to be the mechanism in which opened the channel but through thorough review and analysis we learned that the repulsion and the re-interaction of these residues are the key driving components to MMPL3 and its ability to translocate TMM.

The residues within the inhibitor binding pocket undergo slight conformational changes due to binding of SQ109 with the Tyr646 region of MmpL3, resulting in a conformationally locked open state, but does not completely inhibit water translocation, just slowing it. In fact, translocating protons and water can trigger conformational changes that permit their passing through biological channels. Watkins et al. demonstrated that the M2 water wire donates a proton to His37 and alters its protonation state, increasing its electrostatic repulsion toward the other His37 residues in the tetramer, causing the Trp41 gating residues to spread apart, and opening the channel for water efflux. Disrupting the water wire in M2 at or before His37 prevents channel opening and proton import into the influenza viral envelope, inactivating the virus before replication.^50^ Similarly, in MmpL3, protonation of Asp256 in the Asp256–Tyr646 interaction likely causes electrostatic repulsion toward Tyr 646, causing the TM domain to open. While the deprotonation of His37 residues in influenza M2 channel causes the channel to close by the loss of electrostatic repulsion, MMPL3’s deprotonation of Asp256 will cause the interaction to occur again, thus closing the channel. From our MD simulations, Asp256–Tyr646 and Phe649 of MmpL3 apo- and holo-form likely act similarly to His37 and Trp41 in M2, where electrostatic repulsion may cause a conformational shift to allow TMM binding and translocation.

From Tyr646 being 100% conserved within the MmpL family and participating in every observed water passage event, our data suggest that Tyr646 is the key residue involved in the proton transport pathway of MmpL3 and a key feature in which conformational states are achieved, as any TM open state also exhibits a Tyr 646 flipped away from its opposing Asp 256 residue, showing that the key to this interaction is not that the Tyr 646 residue is interacting with Asp256 but that it becomes protonated and repulses Tyr 646. Indeed, the key residues identified in our study are consistent with the ones identified from a mutagenesis study of the MmpL3 function by Belardinelli et al.,^57^ where mutation of 5 residues in the core TMD (Asp251, Ser288, Gly543, Asp640, and Tyr641) to Cys prevented *Mtb* rescue. Interestingly, Asp256, Ser293, Asp645, and Tyr646 in both Apo and Holo systems interacted with water in our study, suggesting that these residues are critical for protonation and MmpL3 function.

**Figure 15:**
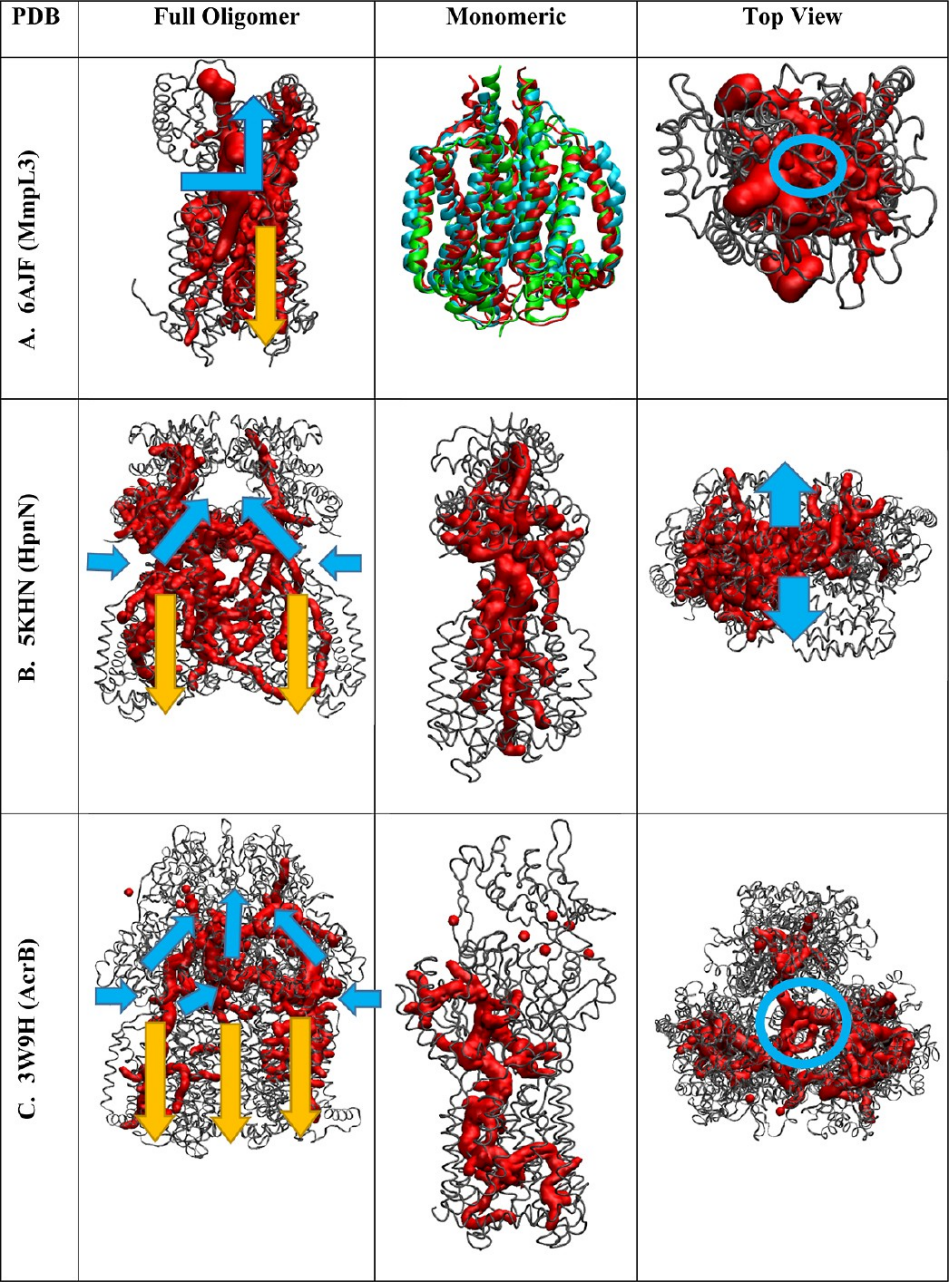
Channel analysis of RND transporters. (A) MmpL3 monomeric RND transporter with structural alignment of HpnN and AcrB for TMD. (B) Dimeric RND transporter HpnN. (C) Trimeric RND transporter AcrB. Substrate entry, transport, and exit pathways through PD to funnel domains/periplasmic space shown with blue arrows. The exit channel for the substrate transport pathway is shown with a blue circle (top view). PMF direction through each monomeric subunit is shown with yellow arrows (column 2).

In contrast to gramicidin and influenza M2 uniporters, which transport water and protons in one direction, RND transporters such as HpnN, AcrB, MmpL5, and MmpL3 antiporters couple proton influx with substrate outflux.^33^ In contrast, MmpL3 is a monomer, and other RND transporters such as MmpL5 (trimer), AcrB (trimer), and HpnN (dimer) show different mechanisms of substrate transport, as their substrate channels lead to the funnel domains connected above the PD (Figure S5D,E). The channels of HpnN and AcrB also lead to the center of their respective oligomeric structures, which leads to a large funnel domain for substrate efflux out of the cell, as shown in Figure 15B,C. MmpL3, on the other hand, is a transporter to periplasmic space through the top of the PD, as shown in Figures 7 and 15A. The use of the trimer and dimer oligomeric states might provide a much greater PMF (multiple effects) than MmpL3’s monomeric PMF. This difference could be key in overcoming the high energetic cost of drug efflux rather than MmpL3’s TMM cell wall component transport. Through all three structures, the TMD of three RND transporters is structurally conserved (Figure 15A/monomeric), suggesting a conserved water/proton transporting mechanism, so is the substrate’s initial binding and entry site next to TM8–TM7. MmpL5 is a trimer that exhibits inter-protein diffusion of water through its center, conferring virulence,^58^ multidrug-efflux,^59^ and siderophore export in iron acquisition.^60^ An MD simulation study investigated MmpL5’s drug-targeting mechanism via inhibiting the substrate-binding sites with pyrazinamide and linezolid, rather than by inhibiting the water wire and proton channel. This showed the slowing of proton movement, suggesting that these processes may be linked by conformational changes in MmpL5 that pump proton-shuttling water molecules at greater capacity, while flipping substrates to the other side of the membrane.

Finally, SQ109 acts as an allosteric inhibitor for TMM translocation. The TMM translocation pathway has been recently proposed by Su et al.^17^ Briefly, after flipping from the inner leaflet membrane layer to the outer leaflet membrane layer, TMM binds between TM7 and TM8 before entering the periplasmic central cavity, whose channel pore size is regulated by the distance between gating residues Ser423 and Asn524. TMM then migrates between periplasmic subdomains 1 (PD1) and 2 (PD2) before finally exiting out into the periplasm.^17^ In our kinetic pathway model, we observed that through the Apo system, the TM and PD domains work in an antiparallel manner. We believe that the binding of TMM occurs in the TM closed state and PD open state (state3) and translocation of TMM through PD occurs in state 2 (TM open state and PD closed state), as the PD entry and exit channels close. Interestingly, rmsd conformational changes observed were very small differences between the TMM-bound cryo EM structures solved by Su et al.^17^ and the apo- and SQ109-bound holo-form crystal structures solved by Zhang et al.^27^ This leads to the conclusion that MMPL3 undergoes very small changes to achieve its functionality, which in previous reports were not as precise. Through thorough analysis of these states, we can propose a coupling mechanism between the open–close motion of PD domains and the open–close motion of TM domains, that is, driven through the water channel protonation, leading to opening of the TM domain, where TM7 and TM8 drive the closing of TMM entry site and exit. Channel analysis shows the entry and exit of the PD domain closed in all TM open states, as Apo state 3 was the only one to show a channel exiting the PD domain. This coupling between TM and PD domains shows the mechanistic insights into MMPL3 and further helps determine that the locked holo state 4 is caused through SQ109 and its disruption of Asp256 and Tyr646, causing the TM channel to never enter a closed state where the PD domain re-opens. From our MD simulations, opening of the TM domain allows for binding of TMM at the TM7–TM8 helices, but without the TM domain closing, the TMM translocation through the PD channel may never occur, suggesting allosteric inhibition by SQ109. Indeed, the truncated channel in our holo-form MD structure (Figure S17) suggests that TMM would face further difficulty in translocating out into the periplasm. With these results, we present the theoretical mechanism for TMM translocation in MMPL3 and allosteric inhibition by SQ109 through a coupling mechanism in which both proton translocation and TMM translocation are dependent mechanisms of each other. Nevertheless, the allosteric inhibition mechanism makes SQ109 a more attractive TB treatment for targeting MmpL3.

## Conclusions

In this study, MD simulations on the apo- and SQ109 holo-form crystal structures of the *Mtb* MmpL3 transporter in explicit membrane were conducted to determine mechanistic insights into the function of MMPL3, to target the inhibition mechanism of SQ109 and to elucidate a water pathway within MMPL3 proton channel. Interestingly, through deeper analysis of the structure of MMPL3, we can determine three conformational states where the TMD opens and closes depending on protonation of Asp256. TMD inversely moves with the PD of MMPL3, showing that there is a proton-dependent mechanism causing conformational state changes. These conformational changes lead to an open PD channel and a closed PD channel where TMM translocation occurs. Conversely in holo form, TM7, TM8, and TM9 all remain in the open state, causing the TMM translocation channel to be closed. While TMM may still enter this PD in the holo form, the holo structure reveals that the proper domain does not reopen to allow for TMM exit. Free energy landscape analysis helped determine thermodynamic states of MMPL3, and normal mode analysis confirmed the anti-parallel movement of TM and PD domains, suggesting the mechanism of SQ109 allosterically inhibits MMPL3 through binding within the water channel, forcing a locked TMD open domain where PD is closed causing inhibition of TMM translocation. Furthermore, in the apo-system, water molecules passed through MmpL3. Hydrogen bonding analysis between five waters and apo MD structure residues revealed high binding occupancies to residues that are highly conserved within the MmpL protein family, indicating that Asp256, Tyr257, Phe260, Leu304, Val638, Asp645, Tyr646, Arg653, Leu678, Leu712, and Asp715 are critical for water and proton passage. Importantly, Asp256–Tyr257 and Asp645–Tyr646 pairs modulate opening/closing of the Phe260 hydrophobic gating residue to allow for water and proton passage. Conversely, in the Holo system, water molecules are less likely to pass, suggesting that SQ109 disrupts the hydrogen-bonded water network and disrupts Asp645–Tyr257 and Asp256–Tyr646 interactions to prevent the channel from closing. This specific ligand analysis led to a better understanding of the protein’s interactions with SQ109.

## Supporting Information Available

Experimental details and images for further understanding and clarification (PDF)
Starting MD simulation systems (.cms) and simulation parameter files (.cfg) for the production run (Topology_Simulation_Protocol) and individual MD simulation trajectory frames (.cms, .mae) which observed water passing in the apo-system (water_passing) and TM7–TM8 conformations in both apo- and holo-systems (TM7–TM8_conformchange) (ZIP)
The full trajectories for each system are available upon request.

## Notes

Protein sequence alignment was obtained from the pfam database. Jalview was used to visualize structure alignments. MOLEonline was used to generate channel pores. Protein structures were obtained from the Protein Data Bank and OPM server. We used Schrodinger 2016-3 for protein preparation. The Desmond package within Schrodinger suite was used to calculate the rmsd timer series and as the MD engine. Caver 3.0 Pymol Extension was used to calculate protein channels and VMD version 1.9.3 was used to generate protein structure representations, draw bonding interactions between residues and SQ109 and measure hydrogen-bonding interactions between waters and MmpL3 channel residues. The implementation of VMD is open-sourced at https://www.ks.uiuc.edu/Research/vmd/vmd-1.9.3/ under Download.
