# Supplementary material for: Inhibition Mechanism of Anti-TB Drug SQ109: Allosteric Inhibition of TMM Translocation of Mycobacterium Tuberculosis MmpL3 Transporter
Source: J Chem Inf Model. 2023 Aug 17;63(16):5356–74. doi: 10.1021/acs.jcim.3c00616 (PMC10466384; doi:10.1021/acs.jcim.3c00616)
Supplement: Supplementary file 1 — ci3c00616_si_001.pdf [file ci3c00616_si_001.pdf]

Supporting Information

Inhibition Mechanism of anti-TB drug SQ109: Allosteric Inhibition of TMM Translocation of Mycobacterium Tuberculosis MmpL3 Transporter

Justin Carbone[1], Nicholas J. Paradis[1], Lucas Bennet[1], Mark C. Alesiani, Katherine R. Hausman, and Chun Wu[*]

Department of Chemistry & Biochemistry, College of Science and Mathematics, Rowan University, Glassboro, New Jersey 08028, United States.

[1] These authors contributed equally

* To whom correspondence should be addressed: wuc@rowan.edu

Table S1. Summary of pfam sequence alignment including the most populated residue at each of the *M. smegmatis* sequence residues and the percent conservation. Conservation of 50% or higher is represented by yellow highlight.

MmpL3 Residue	Consensus Residues	Percentage identity (%)	MmpL3 Residue	Consensus Residues	Percentage identity (%)	MmpL3 Residue	Consensus Residues	Percentage identity (%)
M1	L	16	E46	P	20	T91	D	40
F2	[FL]	20	G47	D	48	E92	[AKRT]	12
A3	P	28	S48	A	64	E93	L	52
W4	R	20	Q49	P	44	L94	V	48
W5	L	28	S50	S	72	D95	D	32
G6	G	36	V51	M	16	Q96	[AEKR]	16
R7	R	68	A52	R	20	V97	L	68
T8	L	16	A53	A	72	V98	R	40
V9	I	44	S54	M	32	K99	A	28
Y10	R	24	L55	[DK]	20	D100	D	72
Q11	R	48	I56	R	32	H101	T	32
F12	F	24	G57	I	28	E102	K	24
R13	A	32	D58	G	32	D103	D	8
Y14	V	28	E59	Q	28	Q104	H	48
I15	P	24	V60	A	28	I105	V	64
V16	I	40	Y61	F	80	V106	Q	24
I17	I	44	G62	P	24	G107	S	28
G18	L	48	R63	E	48	W108	V	44
V19	[ACGV]	16	D64	A	20	V109	Q	44
M20	W	84	R65	D	36	G110	D	56
V21	L	36	T66	S	36	W111	F	40
A22	A	52	S67	D	40	L112	W	40
L23	L	36	H68	S	32	K113	G	36
C24	A	36	V69	V	24	A114	D	44
L25	V	28	V70	R	16	P115	P	76
G26	V	24	A71	A	36	D116	L	36
G27	L	40	I72	[MV]	24	T117	T	4
G28	N	20	L73	V	40	T118	T	4
V29	V	28	T74	V	44	D119	D	4
Y30	[FGL]	16	P75	L	44	P120	[EP]	4
G31	[AV]	36	P76	E	40	T121	T	40
I32	P	60	D77	G	40	V122	A	36
S33	S	36	D78	D	28	S123	A	28
L34	L	76	K79	K	20	A124	G	32
G35	E	36	K80	[GP]	20	M125	L	28
N36	[AE]	20	V81	L	60	K126	Q	28
H37	V	44	T82	G	48	T127	S	84
V38	G	36	D83	D	32	Q128	K	24
T39	Q	28	K84	[AK]	12	D129	D	88
Q40	[AENQR]	12	A85	A	32	L130	G	60
S41	H	28	W86	A	40	R131	K	48
G42	S	36	Q87	H	32	H132	A	64
F43	L	28	K88	A	24	T133	A	48
Y44	S	36	K89	Y	24	F134	Y	48

D45	P	72	V90	Y	56	I135	V	40
**MmpL3 Residue**	**Consensus Residues**	**Percentage identity (%)**	**MmpL3 Residue**	**Consensus Residues**	**Percentage identity (%)**	**MmpL3 Residue**	**Consensus Residues**	**Percentage identity (%)**
S136	Q	28	T181	A	40	L226	R	32
I137	V	48	I182	G	56	G227	G	44
P138	[NT]	16	G183	D	32	I228	I	24
L139	L	52	E184	R	36	M229	V	44
Q140	A	32	D185	S	28	R230	A	44
G141	G	68	Q186	[DLMR]	12	L231	L	24
D142	[DN]	28	K187	[AK]	16	V232	L	64
D143	Q	20	R188	L	20	A233	A	40
D144	G	64	A189	I	44	E234	G	24
D145	T	24	E190	E	44	F235	G	20
E146	E	20	V191	[AL]	24	T236	L	28
I147	L	28	A192	V	32	P237	G	36
L148	A	32	A193	T	36	V238	L	36
K149	E	40	I194	I	24	H239	S	52
N150	S	44	P195	V	24	F240	T	40
Y151	V	32	L196	V	44	F241	F	52
Q152	A	32	V197	I	36	A242	A	40
V153	A	48	A198	L	32	Q243	V	24
V154	V	64	V199	V	48	P244	N	28
E155	R	40	V200	I	28	V245	L	56
P156	D	28	L201	L	92	V246	L	52
E157	I	44	F202	L	56	T247	[TV]	28
L158	V	52	F203	[IL]	32	L248	A	36
Q159	E	20	V204	V	44	I249	L	40
Q160	Q	36	F205	Y	64	G250	A	24
V161	T	24	G206	R	64	L251	I	44
N162	P	24	T207	S	72	G252	G	48
G163	G	68	V208	V	32	I253	A	56
G164	L	56	I209	I	36	A254	G	64
D165	[KT]	28	A210	T	52	I255	T	72
I166	A	44	A211	A	40	D256	D	84
R167	Y	32	A212	L	36	Y257	Y	84
L168	V	48	L213	L	60	G258	A	64
A169	T	60	P214	P	52	L259	I	36
G170	G	88	A215	L	68	F260	F	68
L171	P	60	I216	L	36	I261	L	44
N172	A	56	I217	T	52	V262	V	32
P173	A	44	G218	V	44	S263	G	36
L174	L	32	G219	G	56	R264	R	84
A175	V	24	L220	I	24	F265	Y	68
S176	A	56	A221	S	36	R266	R	36
E177	D	68	I222	L	52	E267	E	64
L178	L	24	A223	A	24	E268	A	32
T179	T	20	G224	[AV]	24	I269	R	40
G180	[DEGH]	12	A225	A	52	A270	R	28

MmpL3 Residue	Consensus Residues	Percentage identity (%)	MmpL3 Residue	Consensus Residues	Percentage identity (%)	MmpL3 Residue	Consensus Residues	Percentage identity (%)
E271	A	20	T316	G	80	N361	F	12
G272	G	72	Y317	P	32	W362	L	48
Y273	E	32	A318	A	36	Q363	Q	4
D274	D	52	I319	[CL]	24	S364	F	4
T275	R	40	I320	A	60	F365	S	4
E276	E	40	A321	I	36	R366	[RT]	4
A277	[AE]	24	S322	G	64	R367	[PR]	4
A278	A	80	V323	V	40	I368	[IP]	4
V279	V	24	M324	L	40	I369	[AI]	4
R280	Y	28	L325	V	40	D370	R	8
R281	T	32	A326	A	40	W371	R	8
T282	A	52	A327	V	48	F372	[EFL]	4
V283	Y	28	I328	L	56	A373	R	8
M284	R	40	L329	A	44	E374	P	12
T285	G	32	S330	A	56	K375	[AGR]	8
S286	T	32	I331	L	52	T376	[GKLPRST]	4
G287	G	44	T332	T	92	Q377	P	8
R288	K	24	V333	L	72	K378	K	12
T289	V	48	L334	L	36	T379	F	24
V290	V	44	A335	P	92	K380	[AD]	20
V291	L	36	A336	A	88	T381	P	76
F292	A	36	A337	L	44	R382	K	36
S293	S	68	L338	L	56	E383	R	52
A294	G	56	A339	A	32	E383	D	20
V295	L	44	I340	L	36	V385	R	24
I296	T	76	L341	A	32	E386	[GKT]	16
I297	V	56	G342	G	56	R387	A	24
V298	A	48	P343	R	56	F388	R	44
A299	G	36	R344	R	36	G389	F	20
S300	A	56	V345	V	12	W390	W	76
S301	[FLMT]	20	D346	[DKR]	4	G391	R	52
V302	L	36	A347	[AKP]	4	R392	R	76
P303	C	32	L348	[AGL]	4	L393	I	28
L304	L	72	G349	G	8	V394	G	60
L305	S	40	V350	[ASV]	4	N395	T	36
L306	F	52	T351	[AKT]	4	V396	A	20
F307	A	40	T352	[GPT]	4	V397	V	56
P308	R	44	L353	[LP]	4	M398	V	56
Q309	L	44	L354	[AL]	4	K399	R	72
G310	P	48	K355	[KR]	4	R400	R	44
F311	Y	24	I356	[IR]	4	P401	P	84
L312	F	52	P357	[FP]	4	I402	[GL]	16
K313	Q	24	F358	[FR]	4	A403	P	40
S314	T	44	L359	[KL]	4	F404	[IV]	28
I315	L	32	A360	G	48	A405	L	60

MmpL3 Residue	Consensus Residues	Percentage identity (%)	MmpL3 Residue	Consensus Residues	Percentage identity (%)	MmpL 3 Residue	Consensus Residues	Percentage identity (%)
A406	V	24	G451	[PQ]	24	K496	[EK]	20
P407	A	40	F452	G	36	M497	C	60
I408	S	36	R453	R	36	W498	W	32
L409	L	28	T454	M	32	K499	S	36
V410	A	36	E455	[GN]	16	E500	A	16
V411	V	32	P456	P	88	R501	R	60
M412	L	40	L457	E	44	P502	S	32
V413	L	32	V458	V	28	A503	F	44
L414	V	44	L459	L	56	N504	[KN]	20
L415	G	32	T460	V	32	D505	N	48
I416	[AL]	32	M461	I	44	S506	P	24
I417	L	48	K462	E	48	G507	D	48
P418	A	36	R463	S	44	S508	G	92
L419	L	44	E464	D	48	K509	H	36
G420	P	36	D465	H	44	D510	A	52
Q421	G	32	G466	D	60	P511	A	48
L422	Y	36	E467	N	40	S512	R	68
S423	R	44	P468	P	56	V513	F	32
L424	T	24	I469	A	32	R514	I	24
G425	G	40	T470	D	32	V515	I	52
G426	Y	48	D471	L	24	I516	[SV]	20
I427	D	32	A472	L	36	Q517	H	32
E428	D	48	Q473	[DV]	24	N518	D	32
S429	R	40	I474	L	40	G519	G	48
K430	K	28	A475	D	32	L520	D	44
Y431	Y	28	D476	K	24	E521	P	80
L432	L	28	M477	L	28	N522	A	36
P433	P	64	R478	A	44	R523	S	60
P434	A	28	A479	K	36	N524	[PTV]	16
D435	S	36	K480	A	24	D525	A	36
N436	T	24	A481	V	32	A526	G	44
A437	P	44	L482	F	20	A527	I	36
V438	S	44	T483	A	16	K528	D	40
R439	N	36	V484	V	48	K529	R	28
Q440	V	24	S485	P	44	I530	I	48
S441	G	64	G486	G	76	D531	[DN]	24
Q442	Y	60	F487	V	44	E532	A	36
E443	A	48	T488	T	64	L533	I	72
Q444	A	60	D489	R	48	R534	R	48
F445	[AL]	32	P490	P	64	A535	L	44
D446	D	32	D491	[DEN]	12	L536	G	36
K447	R	44	N492	G	60	Q537	T	60
L448	H	56	D493	T	16	P538	P	48
F449	F	88	P494	P	36	P539	L	68
P450	P	36	E495	L	24	H540	[DG]	40

MmpL3 Residue	Consensus Residues	Percentage identity (%)	MmpL3 Residue	Consensus Residues	Percentage identity (%)	MmpL3 Residue	Consensus Residues	Percentage identity (%)
G541	G	40	V586	L	28	M631	V	64
I542	A	64	V587	V	52	I632	P	48
E543	Y	23	L588	A	68	G633	A	32
V544	I	56	P589	P	52	L634	M	36
F545	Y	44	I590	L	48	I635	S	44
V546	L	48	K591	V	44	I636	V	48
G547	G	40	A592	I	40	A637	I	44
G548	G	100	A593	V	64	V638	V	52
T549	T	48	L594	G	60	I639	L	84
P550	A	40	M595	T	52	W640	L	40
A551	A	64	S596	V	68	G641	A	72
L552	[TV]	24	A597	[AL]	32	L642	V	52
E553	F	28	L598	L	72	S643	G	80
Q554	L	36	T599	S	80	T644	S	36
D555	D	96	L600	L	40	D645	D	92
S556	I	44	G601	G	48	Y646	Y	100
I557	R	40	S602	A	64	E647	N	72
H558	D	48	T603	A	44	V648	L	44
S559	G	40	M604	F	48	F649	L	60
L560	A	24	G605	G	96	L650	L	88
F561	[NT]	20	I606	L	52	V651	V	52
D562	Y	24	L607	S	52	S652	S	72
K563	D	80	T608	V	72	R653	R	100
L564	L	60	W609	L	60	M654	F	32
P565	L	20	M610	V	40	V655	K	48
L566	[IL]	40	F611	W	48	E656	E	68
M567	A	36	V612	Q	76	A657	E	88
A568	G	32	D613	H	44	R658	Y	8
L569	V	36	G614	I	36	E659	V	12
I570	V	24	H615	L	64	R660	I	32
L571	A	24	G616	G	68	G661	G	32
I572	L	52	S617	I	32	M662	A	44
V573	C	36	G618	E	36	S663	G	72
T574	L	52	L619	[GL]	16	T664	L	32
T575	I	44	M620	L	60	A665	R	32
T576	F	60	N621	H	44	E666	T	44
V577	I	40	Y622	W	56	A667	G	64
L578	I	64	T623	E	12	I668	I	52
M579	L	52	P624	T	8	R669	I	60
F580	L	40	Q625	G	16	I670	R	68
L581	I	44	P626	P	20	G671	A	36
A582	L	40	L627	[IV]	8	T672	M	44
F583	T	40	M628	M	12	A673	[AG]	32
G584	R	92	S629	[AS]	8	T674	G	40
S585	S	68	P630	[LS]	24	T675	T	76

MmpL3 Residue	Consensus Residues	Percentage identity (%)	MmpL3 Residue	Consensus Residues	Percentage identity (%)	MmpL3 Residue	Consensus Residues	Percentage identity (%)
G676	G	92	M701	I	40	A726	A	56
R677	G	36	K702	G	36	V727	I	44
L678	V	92	Y703	Q	76	M728	A	56
I679	V	56	L704	V	36	K729	A	44
T680	T	84	A705	G	84	L730	L	80
G681	A	44	F706	T	44	L731	L	68
A682	A	96	G707	T	52	G732	G	92
A683	G	80	L708	I	52	D733	R	60
L684	L	68	L709	G	56	D734	W	48
I685	V	56	I710	L	36	C735	F	40
L686	F	60	A711	G	84	W736	W	92
A687	A	76	L712	L	60	W737	W	96
V688	A	36	L713	L	80	A738	L	24
V689	T	60	L714	F	52	P739	P	100
A690	M	60	D715	D	92	R740	Q	24
G691	A	48	A716	T	68	W741	R	40
A692	S	32	T717	L	52	M742	~	~
F693	[FLM]	32	I718	V	48	K743	~	~
V694	V	44	I719	V	76	R744	~	~
F695	V	32	R720	R	96	V745	~	~
S696	S	68	M721	S	40	Q746	~	~
D697	D	36	F722	F	44	E747	~	~
L698	L	56	L723	M	40	K748	~	~
V699	R	44	V724	V	44			
M700	V	36	P725	P	92			

Table S2. Lined Residues within the channel of Apo (**Column 1)** and Holo (**Column 2)** form from MOLEOnline analysis and the channel residues in contact with the water from the MD simulations of Apo system (**Column 3**). Residues in the bottlenecks of the channel are bolded.

**Channel/Apo**	**Channel/Holo**	**MD/Apo**
V245	**P244**	
**I249**	**V245**	I249
I253	**L248**	
T289	**I249**	
S293	**I253**	S293
I297	D256	I297
F311	Y257	
I315	**F260**	
I319	**R264**	I319
I427	**E267**	
F452	**E271**	
T454	**I427**	
K496	**L642**	
K499	**D645**	
P502	**Y646**	
T549	**F649**	
L634	R653	L634
V638	R660	V638
L642	M662	L642
**D645**<br>**Y646**	I670	D645
V648	T674	Y646
F649	T675	F649
S652	A682	
R653	L686	R653
Q40	L698	
S41	M701	
G42	L708	
F43	G170	
A242	L178	
F452	P244	
T454	V245	
V638	L248	V638
G641	D256	G641
D645	T674	D645
V648		
F649		F649
R653		R653

Table S3. Simulation system parameters.

System	# Atoms	# Waters	# POPC	# Na^+ ions	# Cl^- ions	NPT (ns)	Production run (ns)
Apo-form (3)	72043	15303	111	52	42	1.2	1000
Holo-form* (3)	72043	15303	111	52	42	1.2	1000

*MmpL3-SQ109 complex.

Table S4. Residue binding sites of representative water molecules from each trajectory. Highlighted residues indicate conservation among the MmpL protein family.

Trajectory	Water ID	Trajectory Frames	Binding Residues
6AJF_1	SPC 15047	500	F307, P630
		524	I633, L304, L571
		569	V638, T316, S301, I297
		577	F260, S293, I297, D256, Y257, D645, Y646
		603	F649, I679, D715, T680
		637	D715, S596, S643, L712, R599
		649	S293, D645, Y646
6AJF_2	SPC 14420	640	V298, T316, I320, S301, A637, V638
		659	I297, I319, V638, R316, S300
		667	V686, L712
		668	L642, S643, Y646, L712, D715
		675	F649, R653, L678, I679, G671, T672
	SPC 14714	1121	G310, K313, T316, R236, P237, V238
		1179	I319, V638, L642, S643, L686, I297, I319, I320, S322, V246, I249, T316, V638
		1631	I639, G641, S643, T599, Y646, A711, L712, D715
		1648	D256, F260, R653
6AJF_3	SPC 1874	89	L171, D144, R63, S67
		95	I635, L704
		101	I297, S300, S301, A637, V638
		129	L642, S643, Y646, L686, L708, A711, L712, D715, T599
		279	D256, F260, L678
	SPC 16926	886	F307, T316
		888	I297, V298, S300, S301, T316, I319, I320, L634
		919	F260, S293, I297, D256, Y257, G641, L642, S643, D645, Y646, L712
		1280	T599, Y646, S643, I679, L712, D715,
		1328	T672, R720

Table S5. Hydrogen bonds formed between MmpL3 apo-form (PDB ID: 6AJF) and water residue 15047 throughout the entirety of trajectory one, with a donor-acceptor distance of 4.0 Å and an angle cutoff of 40°. Entries in yellow, orange, and green indicate the interacting residue appeared in five, four, or three of the representative water pathways, respectively. Bold entries indicate the interacting residue is conserved among the MmpL protein family.

H-bond Donor	H-bond Acceptor	Occupancy (%)
SPC15047	S300	3.25
SPC15047	F307	1.85
SPC15047	PRO630	1.7
**SPC15047**	**D715**	**1.45**
SPC15047	F649	1.2
PRO303	SPC15047	0.95
**LEU304**	**SPC15047**	**0.9**
**TYR257**	**SPC15047**	**0.8**
**SPC15047**	**LEU304**	**0.7**
**ASH256**	**SPC15047**	**0.7**
SPC15047	S301	0.65
**LEU571**	**SPC15047**	**0.5**
**ASH645**	**SPC15047**	**0.5**
SPC15047	GLU647	0.5
**SPC15047**	**LEU712**	**0.45**
LEU312	SPC15047	0.4
**S293**	**SPC15047**	**0.35**
**SPC15047**	**ASH256**	**0.35**
**R720**	**SPC15047**	**0.35**
MET567	SPC15047	0.3
**SPC15047**	**TYR646**	**0.3**
SPC15047	D734	0.3
SPC15047	ILE679	0.25
SPC15047	D143	0.25
SPC15047	LEU619	0.25
ILE297	SPC15047	0.2
**SPC15047**	**S293**	**0.2**
**SPC15047**	**S293**	**0.2**
ILE679	SPC15047	0.2
ALA716	SPC15047	0.2
SPC15047	D491	0.2
SPC15047	D78	0.2
SPC15047	GLU666	0.15
SPC15047	D697	0.15
SPC15047	HIS540	0.15
**V638**	SPC15047	0.15
ILE319	SPC15047	0.15
F649	SPC15047	0.15
SPC15047	THR599	0.15
SPC15047	D446	0.15
SPC15047	GLU102	0.15
SPC15047	D510	0.1
SPC15047	ALA347	0.1
SPC15047	S485	0.1
SPC15047	THR470	0.1
SPC15047	GLU666	0.1
SPC15047	TYR14	0.1
LYS313	SPC15047	0.1
R523	SPC15047	0.1
SPC15047	GLU553	0.1
G633	SPC15047	0.1
SPC15047	MET567	0.1
**V638**	**SPC15047**	**0.1**
**SPC15047**	**V638**	**0.1**
SPC15047	ILE297	0.1
ILE253	SPC15047	0.1
**TYR646**	**SPC15047**	**0.1**
ALA683	SPC15047	0.1
MET595	SPC15047	0.1
S643	SPC15047	0.1
THR599	SPC15047	0.1
**SPC15047**	**TYR646**	**0.1**
**LEU650**	**SPC15047**	**0.1**
**SPC15047**	**ASH645**	**0.1**
**SPC15047**	**ASH645**	**0.1**
SPC15047	GLU102	0.1
GLU102	SPC15047	0.1
SPC15047	GLU92	0.1
SPC15047	GLU59	0.1
SPC15047	GLU146	0.1
SPC15047	GLU659	0.1
**SPC15047**	**G272**	**0.1**
R188	SPC15047	0.1
SPC15047	V396	0.1
SPC15047	D491	0.1
SPC15047	D77	0.1
Q160	SPC15047	0.1
SPC15047	D119	0.1
SPC15047	G618	0.1
SPC15047	GLU543	0.1
SPC15047	GLU464	0.1
SPC15047	D525	0.1
SPC15047	ASN395	0.1
SPC15047	GLU184	0.1
SPC15047	D476	0.1
**SPC15047**	**LEU139**	**0.1**
ASN524	SPC15047	0.1
D505	SPC15047	0.05
THR352	SPC15047	0.05
G349	SPC15047	0.05
LYS529	SPC15047	0.05
ILE32	SPC15047	0.05
R281	SPC15047	0.05
Q517	SPC15047	0.05
THR82	SPC15047	0.05
HIS37	SPC15047	0.05
SPC15047	LEU312	0.05
ALA637	SPC15047	0.05
S301	SPC15047	0.05
SPC15047	G633	0.05
SPC15047	ALA637	0.05
**SPC15047**	**LEU304**	**0.05**
**SPC15047**	**LEU571**	**0.05**
THR316	SPC15047	0.05
S301	SPC15047	0.05
**SPC15047**	**TYR257**	**0.05**
ILE253	SPC15047	0.05
ILE679	SPC15047	0.05
**ALA682**	**SPC15047**	**0.05**
LEU686	SPC15047	0.05
**SPC15047**	**THR680**	**0.05**
**LEU712**	**SPC15047**	**0.05**
**F260**	**SPC15047**	**0.05**
**TYR646**	**SPC15047**	**0.05**
**V651**	**SPC15047**	**0.05**
GLU647	SPC15047	0.05
LYS591	SPC15047	0.05
V724	SPC15047	0.05
SPC15047	ALA711	0.05
SPC15047	LEU581	0.05
ASN621	SPC15047	0.05
THR623	SPC15047	0.05
THR623	SPC15047	0.05
GLU59	SPC15047	0.05
V191	SPC15047	0.05
SPC15047	ILE469	0.05
LYS399	SPC15047	0.05
SPC15047	GLU532	0.05
LYS126	SPC15047	0.05
PRO502	SPC15047	0.05
GLU495	SPC15047	0.05
SPC15047	S136	0.05
F134	SPC15047	0.05
MET125	SPC15047	0.05
LYS447	SPC15047	0.05
LYS462	SPC15047	0.05
LYS528	SPC15047	0.05
SPC15047	D465	0.05
Q49	SPC15047	0.05
**GLU656**	**SPC15047**	**0.05**
**R264**	**SPC15047**	**0.05**
PRO237	SPC15047	0.05
GLU146	SPC15047	0.05
GLU271	SPC15047	0.05
R463	SPC15047	0.05
SPC15047	GLU184	0.05
LYS187	SPC15047	0.05
SPC15047	S423	0.05
THR66	SPC15047	0.05
MET477	SPC15047	0.05
SPC15047	R344	0.05
D435	SPC15047	0.05
D142	SPC15047	0.05
V106	SPC15047	0.05
SPC15047	R523	0.05
GLU429	SPC15047	0.05
Q625	SPC15047	0.05
LYS430	SPC15047	0.05
ASN522	SPC15047	0.05

**THR680**	**SPC15047**	**0.1**	SPC15047	S643	0.05			
**SPC15047**	**LEU712**	**0.1**	SPC15047	ILE719	0.05			

Table S6. Hydrogen bonds formed between MmpL3 apo-form (PDB ID: 6AJF) and water residue 14420 throughout the entirety of trajectory two, with a donor-acceptor distance of 4.0 Å and an angle cutoff of 40°. Entries in yellow, orange, and green indicate the interacting residue appeared in five, four, or three of the representative water pathways, respectively. Bold entries indicate the interacting residue is conserved among the MmpL protein family.

H-bond Donor	H-bond Acceptor	Occupancy (%)	H-bond Donor	H-bond Acceptor	Occupancy (%)	H-bond Donor	H-bond Acceptor	Occupancy (%)
SPC14420	S301	1.8	PRO511	SPC14420	0.1	SPC14420	G671	0.05
SPC14420	THR316	0.6	G349	SPC14420	0.1	THR672	SPC14420	0.05
SPC14420	ALA637	0.6	SPC14420	THR352	0.1	LEU55	SPC14420	0.05
ILE319	SPC14420	0.45	SPC14420	GLU495	0.1	R63	SPC14420	0.05
**SPC14420**	**D715**	**0.4**	SPC14420	ALA475	0.1	HIE68	SPC14420	0.05
**R653**	**SPC14420**	**0.3**	SPC14420	R744	0.1	R453	SPC14420	0.05
SPC14420	GLU184	0.25	SPC14420	GLU102	0.1	SPC14420	V153	0.05
**SPC14420**	**TYR646**	**0.25**	R660	SPC14420	0.1	**V154**	**SPC14420**	**0.05**
SPC14420	F452	0.25	SPC14420	D165	0.1	SPC14420	D103	0.05
SPC14420	GLU455	0.25	SPC14420	LEU354	0.1	R514	SPC14420	0.05
SPC14420	GLU521	0.2	SPC14420	D446	0.1	THR470	SPC14420	0.05
SPC14420	ILE297	0.2	SPC14420	G466	0.1	LYS748	SPC14420	0.05
LEU171	SPC14420	0.2	**R264**	**SPC14420**	**0.1**	Q159	SPC14420	0.05
SPC14420	Q517	0.2	SPC14420	S263	0.1	R13	SPC14420	0.05
**SPC14420**	**GLU267**	**0.2**	**R7**	**SPC14420**	**0.05**	F43	SPC14420	0.05
SPC14420	GLU276	0.15	R65	SPC14420	0.05	S41	SPC14420	0.05
V291	SPC14420	0.15	V438	SPC14420	0.05	SPC14420	S67	0.05
THR316	SPC14420	0.15	R392	SPC14420	0.05	SPC14420	S54	0.05
R266	SPC14420	0.15	LYS80	SPC14420	0.05	Q128	SPC14420	0.05
SPC14420	GLU46	0.15	GLU102	SPC14420	0.05	GLU521	SPC14420	0.05
**SPC14420**	**S652**	**0.15**	LYS79	SPC14420	0.05	GLU659	SPC14420	0.05
SPC14420	D465	0.1	D119	SPC14420	0.05	R740	SPC14420	0.05
SPC14420	GLU177	0.1	**THR488**	**SPC14420**	**0.05**	LYS499	SPC14420	0.05
ASN395	SPC14420	0.1	SPC14420	ILE182	0.05	SPC14420	ASN172	0.05
SPC14420	GLU271	0.1	ILE182	SPC14420	0.05	D144	SPC14420	0.05
SPC14420	D78	0.1	Q243	SPC14420	0.05	PRO434	SPC14420	0.05
SPC14420	D119	0.1	G183	SPC14420	0.05	SPC14420	HIS558	0.05
SPC14420	F487	0.1	SPC14420	HIS615	0.05	V106	SPC14420	0.05
**SPC14420**	**THR488**	**0.1**	R344	SPC14420	0.05	PRO502	SPC14420	0.05
SPC14420	G183	0.1	R344	SPC14420	0.05	ALA503	SPC14420	0.05
SPC14420	D525	0.1	TYR273	SPC14420	0.05	R167	SPC14420	0.05
SPC14420	TYR273	0.1	**D274**	**SPC14420**	**0.05**	G618	SPC14420	0.05
SPC14420	R344	0.1	SPC14420	THR674	0.05	SPC14420	THR352	0.05
**SPC14420**	**D274**	**0.1**	Q11	SPC14420	0.05	**THR488**	**SPC14420**	**0.05**
SPC14420	HIS540	0.1	S559	SPC14420	0.05	**SPC14420**	**LYS149**	**0.05**
R131	SPC14420	0.1	HIS540	SPC14420	0.05	Q152	SPC14420	0.05
SPC14420	D489	0.1	SPC14420	V90	0.05	V153	SPC14420	0.05
**SPC14420**	**PRO490**	**0.1**	GLU93	SPC14420	0.05	F205	SPC14420	0.05
THR285	SPC14420	0.1	LEU94	SPC14420	0.05	F205	SPC14420	0.05
SPC14420	F2	0.1	SPC14420	V291	0.05	**SPC14420**	**THR675**	**0.05**
R288	SPC14420	0.1	ILE320	SPC14420	0.05	SPC14420	V290	0.05
SPC14420	V298	0.1	S301	SPC14420	0.05	V290	SPC14420	0.05
**SPC14420**	**V638**	**0.1**	**V638**	**SPC14420**	**0.05**	R167	SPC14420	0.05
**LEU712**	**SPC14420**	**0.1**	SPC14420	THR316	0.05	G206	SPC14420	0.05
**SPC14420**	**LEU712**	**0.1**	**SPC14420**	**LEU304**	**0.05**	R677	SPC14420	0.05
F649	SPC14420	0.1	ILE297	SPC14420	0.05	SPC14420	ALA3	0.05
SPC14420	LEU678	0.1	S300	SPC14420	0.05	PRO450	SPC14420	0.05
SPC14420	S263	0.1	SPC14420	ALA637	0.05	S512	SPC14420	0.05
SPC14420	F452	0.1	SPC14420	LEU634	0.05	SPC14420	THR74	0.05

**SPC14420**	**LEU34**	**0.1**	**V638**	**SPC1442 0**	**0.05**	SPC14420	S286	0.05
SPC14420	Q40	0.1	SPC14420	LEU686	0.05	**SPC1442 0**	**F260**	**0.05**
SPC14420	D144	0.1	LEU642	SPC14420	0.05	S286	SPC14420	0.05
SPC14420	LEU520	0.1	**SPC1442 0**	**LEU712**	**0.05**	ALA347	SPC14420	0.05
SPC14420	GLU659	0.1	SPC14420	ILE639	0.05	R288	SPC14420	0.05
SPC14420	GLU271	0.1	ALA716	SPC14420	0.05			
SPC14420	D144	0.1	ILE679	SPC14420	0.05			

Table S7. Hydrogen bonds formed between Mmpl3 apo-form (PDB ID: 6AJF) and water residue 14714 throughout the entirety of trajectory two, with a donor-acceptor distance of 4.0 Å and an angle cutoff of 40°. Entries in yellow, orange, and green indicate the interacting residue appeared in five, four, or three of the representative water pathways, respectively. Bold entries indicate the interacting residue is conserved among the MmpL protein family.

H-bond Donor	H-bond Acceptor	Occupancy (%)	H-bond Donor	H-bond Acceptor	Occupancy (%)	H-bond Donor	H-bond Acceptor	Occupancy (%)
SPC14714	S301	10.19	SPC14714	S300	0.25	SPC14714	LEU520	0.1
SPC14714	ALA637	8.9	S643	SPC14714	0.25	R63	SPC14714	0.1
SPC14714	S300	4.6	SPC14714	GLU59	0.2	R167	SPC14714	0.1
SPC14714	S322	4	TYR431	SPC14714	0.2	SPC14714	GLU464	0.1
ILE297	SPC14714	4	S428	SPC14714	0.2	**SPC14714**	**R534**	**0.05**
**SPC14714**	**V638**	**3.65**	SPC14714	GLU271	0.2	SPC14714	D555	0.05
**V638**	**SPC14714**	**2.95**	SPC14714	ALA233	0.2	LYS496	SPC14714	0.05
ILE319	SPC14714	2.85	ILE222	SPC14714	0.2	R669	SPC14714	0.05
SPC14714	ILE297	2.6	SPC14714	ILE319	0.2	LYS187	SPC14714	0.05
SPC14714	THR316	2.3	SPC14714	ALA637	0.2	R534	SPC14714	0.05
ILE319	SPC14714	1.9	SPC14714	ILE249	0.2	ALA535	SPC14714	0.05
THR316	SPC14714	1.9	LEU634	SPC14714	0.2	SPC14714	V109	0.05
SPC14714	THR236	1.5	SPC14714	ILE297	0.2	THR66	SPC14714	0.05
SPC14714	S322	1.45	SPC14714	V298	0.2	THR66	SPC14714	0.05
SPC14714	G250	1.45	SPC14714	D435	0.2	V109	SPC14714	0.05
G641	SPC14714	1.45	SPC14714	ASN395	0.15	ASN436	SPC14714	0.05
ILE249	SPC14714	1.1	SPC14714	THR236	0.15	G618	SPC14714	0.05
V246	SPC14714	1.05	**LEU712**	**SPC14714**	**0.15**	SPC14714	TRP108	0.05
**V638**	**SPC14714**	**1.05**	LEU708	SPC14714	0.15	TRP108	SPC14714	0.05
ILE297	SPC14714	0.95	SPC14714	THR39	0.15	LYS113	SPC14714	0.05
S301	SPC14714	0.9	SPC14714	GLU500	0.15	PRO76	SPC14714	0.05
**SPC14714**	**D715**	**0.8**	SPC14714	MET398	0.15	LYS499	SPC14714	0.05
SPC14714	ILE319	0.75	**SPC14714**	**D274**	**0.1**	D142	SPC14714	0.05
SPC14714	V245	0.65	SPC14714	GLU495	0.1	Q625	SPC14714	0.05
ILE320	SPC14714	0.65	SPC14714	GLU146	0.1	PRO626	SPC14714	0.05
THR316	SPC14714	0.65	SPC14714	S263	0.1	LYS430	SPC14714	0.05
S322	SPC14714	0.6	SPC14714	D77	0.1	R523	SPC14714	0.05
SPC14714	LEU248	0.55	SPC14714	D78	0.1	LYS509	SPC14714	0.05
S300	SPC14714	0.55	SPC14714	D491	0.1	Q140	SPC14714	0.05
SPC14714	ALA711	0.55	SPC14714	D142	0.1	LYS729	SPC14714	0.05
SPC14714	MET229	0.5	**SPC14714**	**G141**	**0.1**	SPC14714	GLU666	0.05
V238	SPC14714	0.5	SPC14714	PRO626	0.1	R514	SPC14714	0.05
LEU642	SPC14714	0.5	SPC14714	Q11	0.1	SPC14714	D525	0.05
SPC14714	ALA221	0.5	SPC14714	ILE269	0.1	**R264**	**SPC14714**	**0.05**
V232	SPC14714	0.45	SPC14714	D697	0.1	LEU448	SPC14714	0.05
ALA221	SPC14714	0.45	SPC14714	GLU521	0.1	LYS99	SPC14714	0.05
**SPC14714**	**LEU304**	**0.45**	SPC14714	D83	0.1	LYS79	SPC14714	0.05
V246	SPC14714	0.45	SPC14714	D119	0.1	MET284	SPC14714	0.05
SPC14714	PRO237	0.4	**R653**	**SPC14714**	**0.1**	TRP741	SPC14714	0.05
ALA326	SPC14714	0.4	SPC14714	V655	0.1	R740	SPC14714	0.05
SPC14714	LEU686	0.4	TYR273	SPC14714	0.1	SPC14714	GLU271	0.05
**SPC14714**	**GLU267**	**0.35**	SPC14714	G310	0.1	THR352	SPC14714	0.05
SPC14714	V232	0.35	SPC14714	V232	0.1	Q537	SPC14714	0.05
ALA254	SPC14714	0.35	SPC14714	V238	0.1	SPC14714	D185	0.05
**LEU304**	**SPC14714**	**0.35**	SPC14714	V246	0.1	GLU543	SPC14714	0.05
LEU642	SPC14714	0.35	V245	SPC14714	0.1	**SPC14714**	**S652**	**0.05**
**SPC14714**	**V638**	**0.35**	G250	SPC14714	0.1	F583	SPC14714	0.05
SPC14714	LEU634	0.35	V689	SPC14714	0.1	**V651**	**SPC14714**	**0.05**
ALA637	SPC14714	0.35	ILE249	SPC14714	0.1	G451	SPC14714	0.05
SPC14714	Q625	0.3	LEU686	SPC14714	0.1	SPC14714	HIS37	0.05
SPC14714	ILE222	0.3	ILE253	SPC14714	0.1	**D100**	**SPC14714**	**0.05**

V323	SPC14714	0.3	SPC14714	LEU634	0.1	LYS313	SPC14714	0.05
SPC14714	S301	0.3	SPC14714	ILE320	0.1	PRO237	SPC14714	0.05
SPC14714	V323	0.3	SPC14714	LEU312	0.1	ALA233	SPC14714	0.05
SPC14714	G426	0.25	ILE639	SPC14714	0.1	V238	SPC14714	0.05
THR236	SPC14714	0.25	**TYR646**	**SPC14714**	**0.1**			
SPC14714	THR316	0.25	V655	SPC14714	0.1			

Table S8. Hydrogen bonds formed between MmpL3 apo-form (PDB ID: 6AJF) and water residue 1874 throughout the entirety of trajectory three, with a donor-acceptor distance of 4.0 Å and an angle cutoff of 40°. Entries in yellow, orange, and green indicate the interacting residue appeared in five, four, or three of the representative water pathways, respectively. Bold entries indicate the interacting residue is conserved among the MmpL protein family.

H-bond Donor	H-bond Acceptor	Occupancy (%)
**SPC1874**	**D715**	**3.75**
SPC1874	LEU708	3.45
**TYR646**	**SPC1874**	**3.1**
SPC1874	ALA637	1.85
SPC1874	ALA683	1.8
ALA711	SPC1874	1
SPC1874	LEU642	0.95
SPC1874	ILE297	0.9
**LEU712**	**SPC1874**	**0.85**
SPC1874	S300	0.8
LEU686	SPC1874	0.8
LEU642	SPC1874	0.7
**ASH256**	**SPC1874**	**0.65**
SPC1874	LEU686	0.6
SPC1874	ALA711	0.6
**SPC1874**	**TYR646**	**0.6**
S301	SPC1874	0.55
S643	SPC1874	0.45
SPC1874	LEU642	0.35
**V638**	**SPC1874**	**0.35**
ILE297	SPC1874	0.3
SPC1874	ILE639	0.3
**SPC1874**	**ASH645**	**0.3**
**SPC1874**	**V638**	**0.25**
SPC1874	LEU686	0.25
**SPC1874**	**LEU712**	**0.25**
SPC1874	D733	0.25
SPC1874	D143	0.2
THR603	SPC1874	0.2
S643	SPC1874	0.2
ALA716	SPC1874	0.2
SPC1874	D95	0.2
SPC1874	GLU495	0.2
**SPC1874**	**LEU712**	**0.15**
SPC1874	ALA711	0.15
SPC1874	S643	0.15
SPC1874	F649	0.15
**D715**	**SPC1874**	**0.15**
R392	SPC1874	0.15
SPC1874	GLU92	0.15
SPC1874	D142	0.1
SPC1874	D103	0.1
SPC1874	D531	0.1
SPC1874	Q243	0.1
SPC1874	GLU146	0.1
SPC1874	D144	0.1
SPC1874	D64	0.1
SPC1874	LEU704	0.1
LEU708	SPC1874	0.1
G641	SPC1874	0.1
SPC1874	S301	0.1
SPC1874	LEU248	0.1

H-bond Donor	H-bond Acceptor	Occupancy (%)
LEU714	SPC1874	0.1
ALA711	SPC1874	0.1
SPC1874	THR603	0.1
**ASH645**	**SPC1874**	**0.1**
LEU678	SPC1874	0.1
**SPC1874**	**ASH256**	**0.1**
**SPC1874**	**R653**	**0.1**
SPC1874	Q746	0.1
SPC1874	ILE32	0.1
SPC1874	D77	0.1
SPC1874	Q96	0.1
SPC1874	GLU155	0.1
SPC1874	D435	0.1
MET497	SPC1874	0.1
**Q554**	**SPC1874**	**0.1**
SPC1874	PRO156	0.1
Q160	SPC1874	0.1
**SPC1874**	**Q554**	**0.1**
SPC1874	GLU521	0.1
SPC1874	R669	0.1
SPC1874	GLU443	0.1
SPC1874	ASN524	0.1
SPC1874	GLU102	0.1
SPC1874	G47	0.1
V745	SPC1874	0.05
LEU168	SPC1874	0.05
GLU146	SPC1874	0.05
SPC1874	LEU171	0.05
HIE68	SPC1874	0.05
R63	SPC1874	0.05
S67	SPC1874	0.05
S67	SPC1874	0.05
ILE635	SPC1874	0.05
LEU704	SPC1874	0.05
SPC1874	LEU708	0.05
LEU686	SPC1874	0.05
G252	SPC1874	0.05
ALA637	SPC1874	0.05
ILE319	SPC1874	0.05
**SPC1874**	**ALA687**	**0.05**
ALA683	SPC1874	0.05
**SPC1874**	**TYR257**	**0.05**
**SPC1874**	**ASH256**	**0.05**
F649	SPC1874	0.05
SPC1874	S643	0.05
LEU600	SPC1874	0.05
**SPC1874**	**TYR646**	**0.05**
SPC1874	THR599	0.05
THR599	SPC1874	0.05
ILE639	SPC1874	0.05
**F260**	**SPC1874**	**0.05**
LYS89	SPC1874	0.05

H-bond Donor	H-bond Acceptor	Occupancy (%)
SPC1874	G388	0.05
S33	SPC1874	0.05
LYS496	SPC1874	0.05
SPC1874	D119	0.05
PRO76	SPC1874	0.05
THR483	SPC1874	0.05
THR118	SPC1874	0.05
THR118	SPC1874	0.05
ILE32	SPC1874	0.05
SPC1874	G584	0.05
**LEU730**	**SPC1874**	**0.05**
SPC1874	V612	0.05
PRO624	SPC1874	0.05
SPC1874	S485	0.05
LYS529	SPC1874	0.05
SPC1874	ALA527	0.05
SPC1874	ASN621	0.05
SPC1874	R65	0.05
**G519**	**SPC1874**	**0.05**
GLU495	SPC1874	0.05
SPC1874	HIS540	0.05
TYR30	SPC1874	0.05
ALA527	SPC1874	0.05
LYS528	SPC1874	0.05
ILE670	SPC1874	0.05
D510	SPC1874	0.05
THR82	SPC1874	0.05
LYS84	SPC1874	0.05
ASN621	SPC1874	0.05
S485	SPC1874	0.05
R744	SPC1874	0.05
V745	SPC1874	0.05
SPC1874	R131	0.05
GLU464	SPC1874	0.05
F545	SPC1874	0.05
R478	SPC1874	0.05
LYS149	SPC1874	0.05
SPC1874	R439	0.05
D734	SPC1874	0.05
V484	SPC1874	0.05
GLU102	SPC1874	0.05
SPC1874	R266	0.05
S441	SPC1874	0.05
S441	SPC1874	0.05
SPC1874	GLU268	0.05
SPC1874	R281	0.05

Table S9. Hydrogen bonds formed between MmpL3 apo-form (PDB ID: 6AJF) and water residue 16926 throughout the entirety of trajectory three, with a donor-acceptor distance of 4.0 Å and an angle cutoff of 40°. Entries in yellow, orange, and green indicate the interacting residue appeared in five, four, or three of the representative water pathways, respectively. Bold entries indicate the interacting residue is conserved among the MmpL protein family.

H-bond Donor	H-bond Acceptor	Occupancy (%)	H-bond Donor	H-bond Acceptor	Occupancy (%)	H-bond Donor	H-bond Acceptor	Occupancy (%)
SPC16926	G641	21.44	SPC16926	D476	0.1	Q243	SPC16926	0.05
SPC16926	THR644	15.24	LYS496	SPC16926	0.1	R167	SPC16926	0.05
**S293**	**SPC16926**	**14.69**	**SPC16926**	**PRO490**	**0.1**	LYS313	SPC16926	0.05
**SPC16926**	**TYR646**	**1.7**	SPC16926	D78	0.1	S301	SPC16926	0.05
SPC16926	S301	1.65	SPC16926	GLU553	0.1	THR316	SPC16926	0.05
**ASH645**	**SPC16926**	**1.6**	SPC16926	D510	0.1	SPC16926	ILE319	0.05
SPC16926	ILE296	1.45	PRO156	SPC16926	0.1	ALA637	SPC16926	0.05
ILE296	SPC16926	1.4	Q444	SPC16926	0.1	SPC16926	ILE320	0.05
ILE297	SPC16926	1.05	SPC16926	GLU155	0.1	V323	SPC16926	0.05
SPC16926	LEU642	0.95	SPC16926	F307	0.1	ILE320	SPC16926	0.05
SPC16926	THR644	0.8	**SPC16926**	**LEU304**	**0.1**	LEU634	SPC16926	0.05
**ASH256**	**SPC16926**	**0.6**	SPC16926	S301	0.1	SPC16926	ALA299	0.05
**SPC16926**	**D715**	**0.6**	LEU305	SPC16926	0.1	ALA299	SPC16926	0.05
**S293**	**SPC16926**	**0.5**	ILE320	SPC16926	0.1	**SPC16926**	**S293**	**0.05**
ILE319	SPC16926	0.4	SPC16926	ALA294	0.1	V648	SPC16926	0.05
SPC16926	ILE297	0.4	V298	SPC16926	0.1	SPC16926	THR289	0.05
**SPC16926**	**ASH645**	**0.4**	S286	SPC16926	0.1	**TYR257**	**SPC16926**	**0.05**
**TYR646**	**SPC16926**	**0.4**	F649	SPC16926	0.1	**SPC16926**	**ALA682**	**0.05**
SPC16926	THR316	0.35	**F260**	**SPC16926**	**0.1**	ILE679	SPC16926	0.05
SPC16926	S286	0.35	**SPC16926**	**LEU712**	**0.1**	**SPC16926**	**R720**	**0.05**
V724	SPC16926	0.35	SPC16926	THR599	0.1	**LEU712**	**SPC16926**	**0.05**
SPC16926	THR672	0.35	GLU647	SPC16926	0.1	SPC16926	MET595	0.05
SPC16926	V298	0.3	MET595	SPC16926	0.1	S596	SPC16926	0.05
**SPC16926**	**ASH645**	**0.3**	**R720**	**SPC16926**	**0.1**	THR672	SPC16926	0.05
**ASH645**	**SPC16926**	**0.3**	THR599	SPC16926	0.1	SPC16926	S643	0.05
SPC16926	TRP741	0.25	SPC16926	D95	0.1	SPC16926	ALA711	0.05
SPC16926	TRP108	0.25	SPC16926	LEU581	0.1	**TYR646**	**SPC16926**	**0.05**
SPC16926	GLU157		R288	SPC16926	0.1	SPC16926	THR672	0.05
SPC16926	ALA637	0.25	D77	SPC16926	0.1	LYS99	SPC16926	0.05
SPC16926	ILE297	0.25	SPC16926	D697	0.1	TRP4	SPC16926	0.05
**TYR257**	**SPC16926**	**0.25**	SPC16926	R660	0.1	Q160	SPC16926	0.05
G641	SPC16926	0.25	SPC16926	D145	0.1	SPC16926	V204	0.05
ILE679	SPC16926	0.25	SPC16926	TYR44	0.1	SPC16926	G35	0.05
SPC16926	GLU647	0.25	**SPC16926**	**S50**	**0.1**	SPC16926	V38	0.05
SPC16926	D77	0.2	**SPC16926**	**THR121**	**0.1**	GLU157	SPC16926	0.05
SPC16926	D435	0.2	**SPC16926**	**D129**	**0.1**	R463	SPC16926	0.05
SPC16926	D733	0.2	SPC16926	LYS462	0.1	ASN621	SPC16926	0.05
SPC16926	THR39	0.2	R131	SPC16926	0.1	SPC16926	Q746	0.05
SPC16926	THR316	0.2	LYS748	SPC16926	0.05	GLU747	SPC16926	0.05
SPC16926	LEU678	0.2	TRP4	SPC16926	0.05	MET662	SPC16926	0.05
SPC16926	S643	0.2	LYS126	SPC16926	0.05	SPC16926	GLU46	0.05
SPC16926	GLU93	0.2	ILE670	SPC16926	0.05	G548	SPC16926	0.05
SPC16926	GLU443	0.2	ALA270	SPC16926	0.05	SPC16926	F445	0.05
SPC16926	D489	0.2	LYS113	SPC16926	0.05	V70	SPC16926	0.05
SPC16926	TYR622	0.15	SPC16926	Q11	0.05	ALA124	SPC16926	0.05
THR316	SPC16926	0.15	D733	SPC16926	0.05	MET125	SPC16926	0.05
**SPC16926**	**S293**	**0.15**	TRP741	SPC16926	0.05	LYS509	SPC16926	0.05
THR289	SPC16926	0.15	ASN395	SPC16926	0.05	SPC16926	HIS615	0.05
SPC16926	**TYR257**	0.15	Q537	SPC16926	0.05	SPC16926	S263	0.05
V290	SPC16926	0.15	THR118	SPC16926	0.05	**D274**	**SPC16926**	**0.05**
SPC16926	ILE679	0.15	S123	SPC16926	0.05	**G272**	**SPC16926**	**0.05**
**SPC16926**	**F260**	**0.15**	Q49	SPC16926	0.05	SPC16926	GLU464	0.05
S643	SPC16926	0.15	**PRO490**	**SPC16926**	**0.05**	LYS462	SPC16926	0.05
**SPC16926**	**LEU731**	**0.1**	SPC16926	HIS558	0.05	R744	SPC16926	0.05
SPC16926	GLU92	0.1	SPC16926	GLU543	0.05	S617	SPC16926	0.05

SPC16926	ALA665	0.1	**Q554**	**SPC16926**	**0.05**	SPC16926	PRO75	0.05
SPC16926	GLU747	0.1	ASN621	SPC16926	0.05			

Table S10. Trajectory clustering analysis data for each individual trajectory and the two combined trajectories.

Trajectory	Cluster #	# Frames in cluster	Cluster %
6AJF_1	1	1556	77.80
	2	367	18.35
	3	75	3.75
	4	2	0.10
6AJF_2	1	1217	60.94
	2	95	4.76
	3	648	32.45
	4	39	1.95
	5	1	0.05
6AJF_3	1	129	6.45
	2	15	0.75
	3	708	35.40
	4	1148	57.40
6AJG_1	1	561	28.01
	2	2	0.10
	3	447	22.32
	4	317	15.83
	5	128	6.39
	6	547	27.31
6AJG_2	1	1242	62.10
	2	43	2.15
	3	394	19.70
	4	219	10.95
	5	102	5.10
6AJG_3	1	311	15.55
	2	1141	57.05
	3	544	27.20
	4	4	0.20
6AJF (combined)	1	773	25.77
	2	613	20.43
	3	574	19.13
	4	325	10.83
	5	319	10.63
	6	188	6.27
	7	99	3.30
	8	63	2.10
	9	43	1.43
	10	3	0.10
6AJG (combined)	1	621	20.69
	2	564	18.79
	3	384	12.80
	4	285	9.50
	5	271	9.03
	6	214	7.13
	7	197	6.56
	8	197	6.56
	9	109	3.63
	10	88	2.93
	11	51	1.70
	12	18	0.60
	13	2	0.07

Table S11. Kinetic parameters of water, proton and ions transport in various media.

Transport Medium	Method	Water transport time (ns)	Proton transport time (ns)	Water diffusion coefficient (cm^2/s)	Water transport coefficient (cm^2/s)	Proton transport coefficient (cm^2/s)	Na^+ diffusion coefficient (cm^2/s)	Ref
Bulk Water	Experimental	N/A	N/A	**2.3×10^{-5}**	**$2.4x10^{-5}$**	**$9.3x10^{-5}$**	**$1.33x10^{-5}$**	Wraight (2006) Berendsen et al. (1987)
Gramicidin	Experimental	**0.2**	N/A	N/A	**$2.7x10^{-6}$**	**$3x10^{-5}$**	N/A	Wraight (2006) Chiu et al. (1999)
Influenza A M2	MD Simulation	**1-2**	**0.388****	N/A	N/A	**$1.12\text{-}1.88x10^{-5}$**	N/A	Wei & Pohorille (2013) Smondyrev & Voth (2002)
MmpL5	MD Simulation	**20**	N/A	N/A	N/A	N/A	N/A	Sandhu & Akhter (2016)
MmpL3	MD Simulation	**135**	N/A	N/A	N/A	N/A	N/A	This study

*Diffusion coefficients refer to transport within bulk water. Transport coefficients refer to transport across biological channels (i.e. gramicidin, influenza A M2, MmpL5 and MmpL3) without electric field (cm^2/s).
**Value approximated from figure 10. See Smondyrev & Voth (2002).
***N/A: Not available.

Berendsen, H. J. C.; Grigera, J. R.; Straatsma, T. P., The missing term In effective pair potentials. J. Phys. Chem. 1987, 91, 6269-6271.

Chiu, S. W.; Subramaniam, S.; Jakobsson, E., Simulation study of a gramicidin/lipid bilayer system in excess water and lipid. II. Rates and mechanisms of water transport. Biophysical Journal 1999, 76, 1939-1950.

Sandhu, P.; Akhter, Y., The drug binding sites and transport mechanism of the RND pumps from Mycobacterium tuberculosis: Insights from molecular dynamics simulations. Archives of Biochemistry and Biophysics 2016, 592, 38-49.

Smondyrev, A. M.; Voth, G. A., Molecular dynamics simulation of proton transport through the influenza A virus M2 channel. Biophysical Journal 2002, 83, 1987-1996.

Wei, C. Y.; Pohorille, A., Activation and proton transport mechanism in influenza A M2 channel. Biophysical Journal 2013, 105, 2036-2045.

Wraight, C. A., Chance and design - Proton transfer in water, channels and bioenergetic proteins. Biochimica Et Biophysica Acta-Bioenergetics 2006, 1757, 886-912

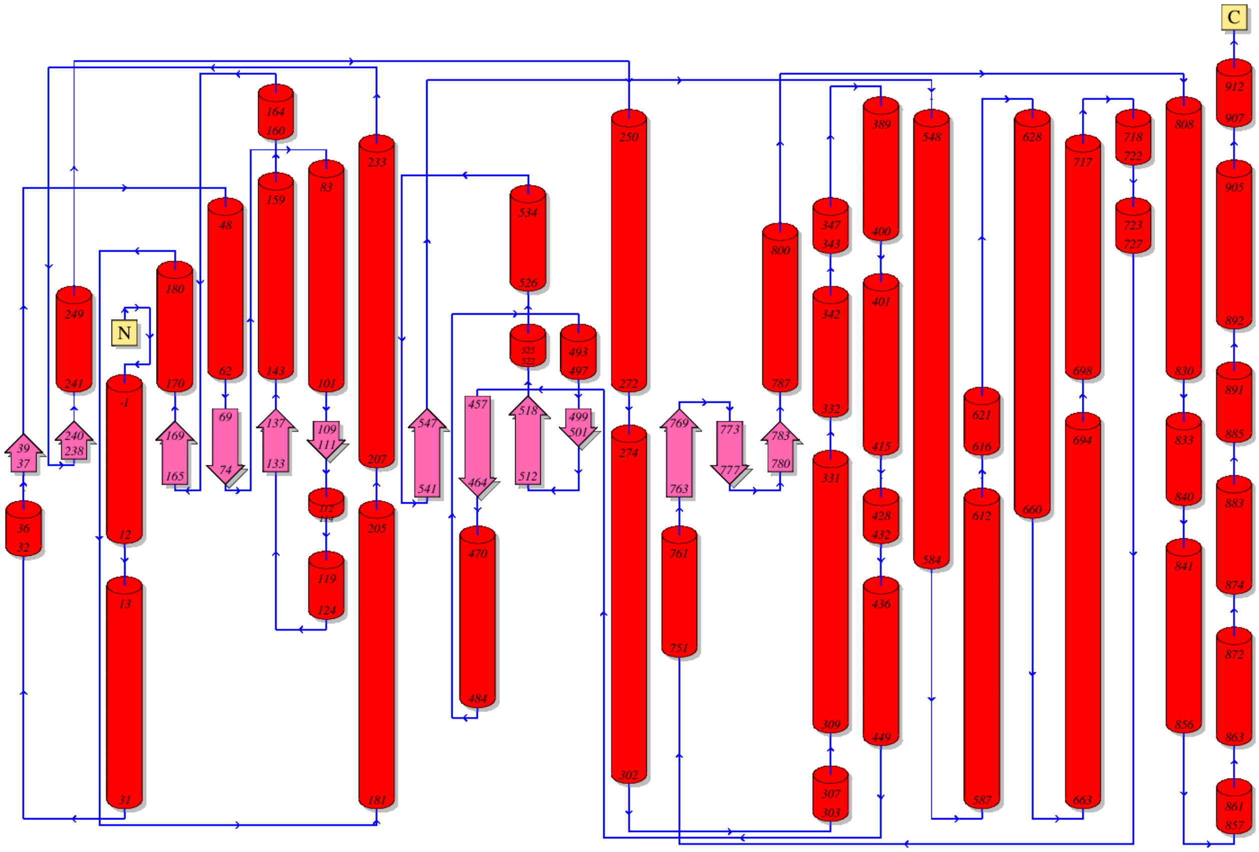

Figure S1. Complete secondary structure of MmpL3 crystal structure (6AJF) from *M. smegmatis*.

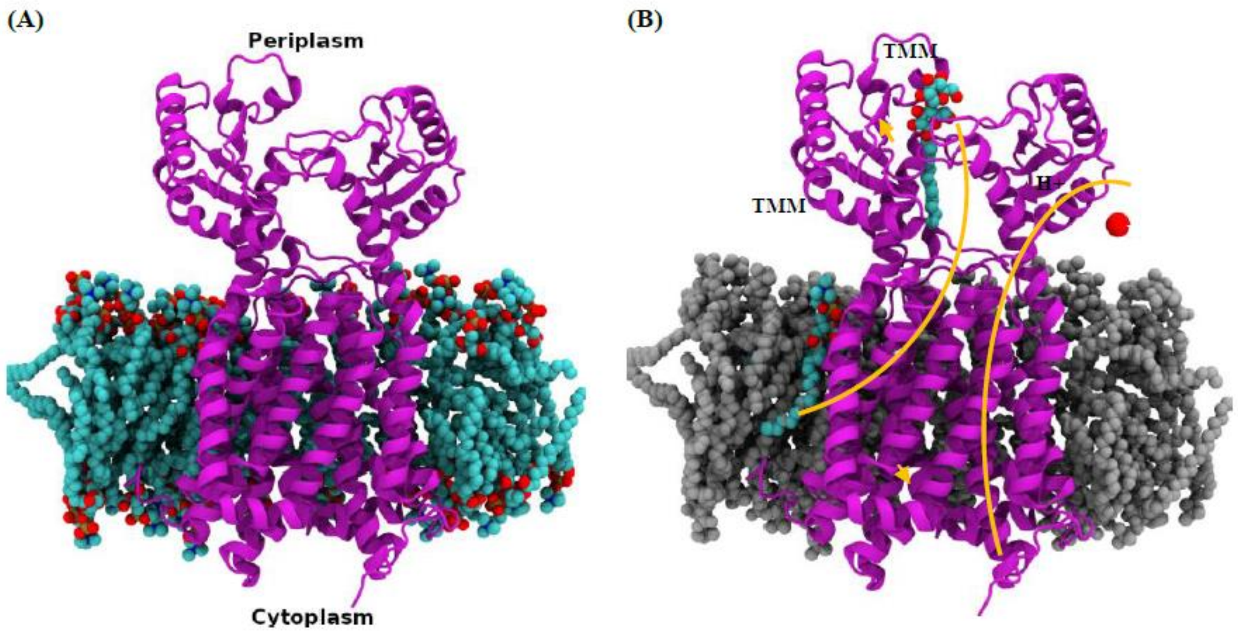

Figure S2. (A) Cartoon representation of *M. smegmatis* MmpL3 (purple ribbon) embedded in a phospholipid bilayer. **(B)** Schematic representation of the currently hypothesized transport mechanism of TMM from the cytoplasm to the periplasm.

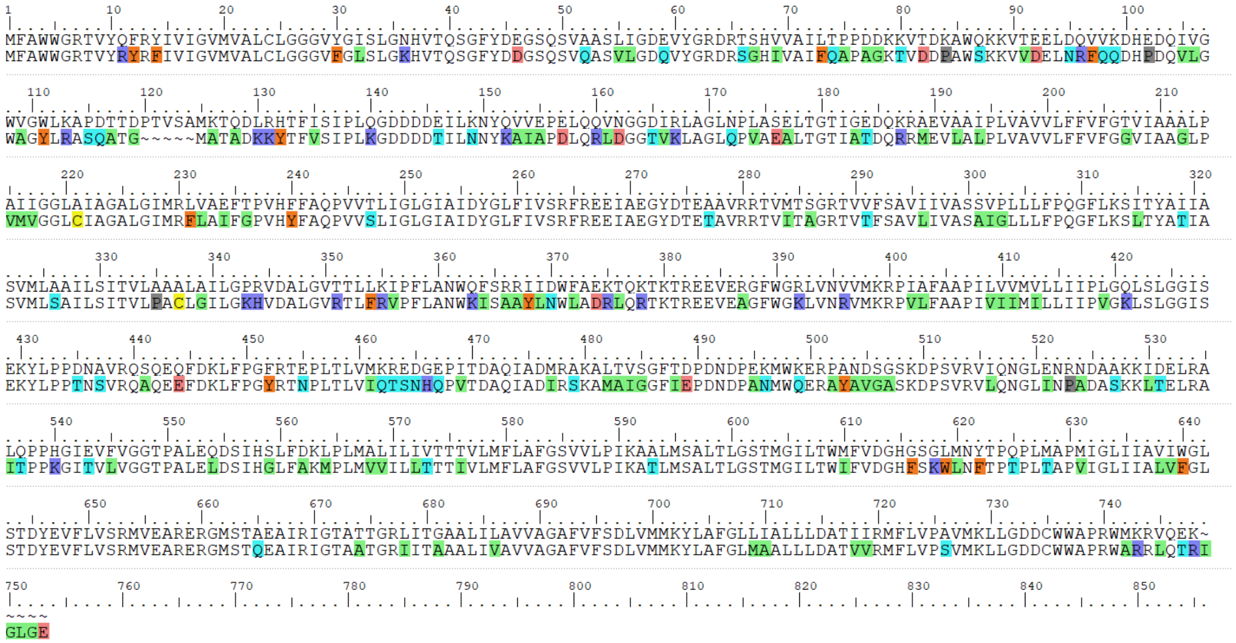

71.9% identity in 748 residues

Figure S3. MmpL3 protein sequence comparison between *M. smegmatis* (top) and *M. tuberculosis* (bottom). Conserved sequences are in white, whereas non-conserved sequences are colored.

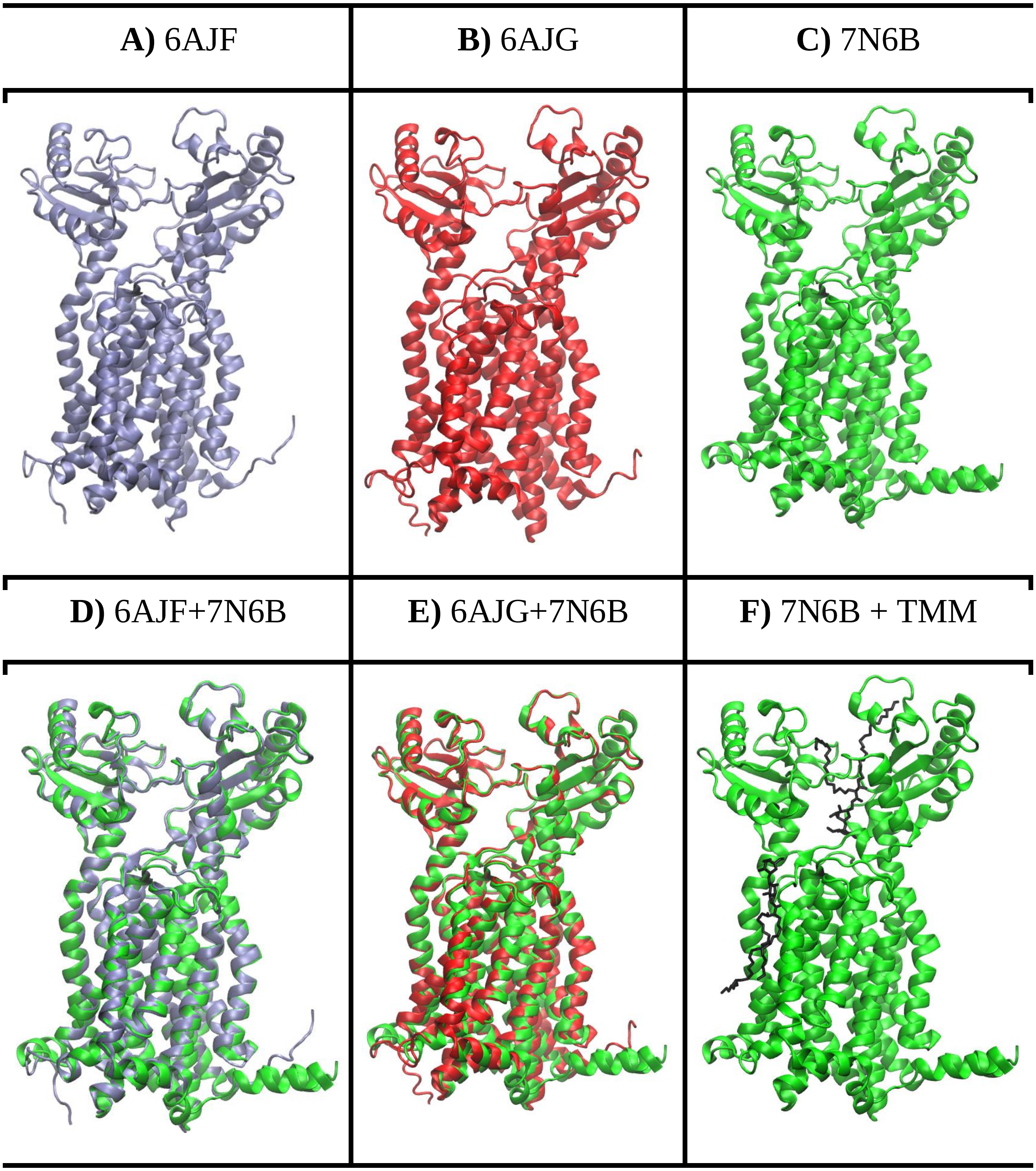

Figure S4. Structure comparison of MmpL3 transporters. **(A & B)** X-ray solved crystal structures of MmpL3 in the apo-form (PDB ID: 6AJF) and inhibitor SQ109-bound holo-form (PDB ID: 6AJG**). (C&F)** Cryo-EM structures of TMM substrate-bound holo-form (PDB ID: 7N6B) of MmpL3, with and without TMM shown (black sticks). **D)** Superimposition of **A & C** (~1.1 Å)**. E)** Superimposition of **B & C** (~1.5 Å).

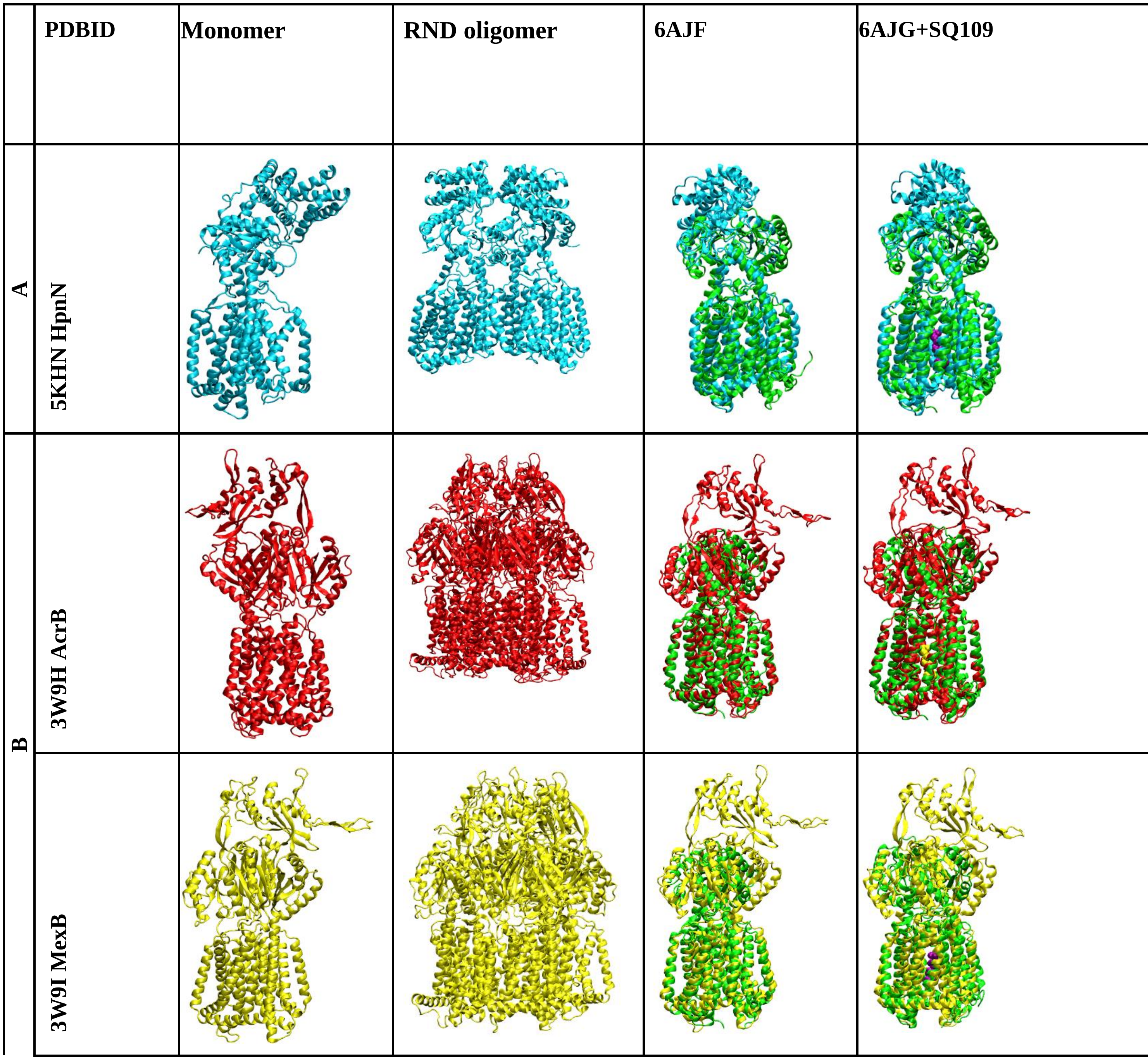
PDBID
Monomer
RND oligomer
6AJF
6AJG+SQ109
A
5KHN HpnN
B
3W9H AcrB
3W9I MexB

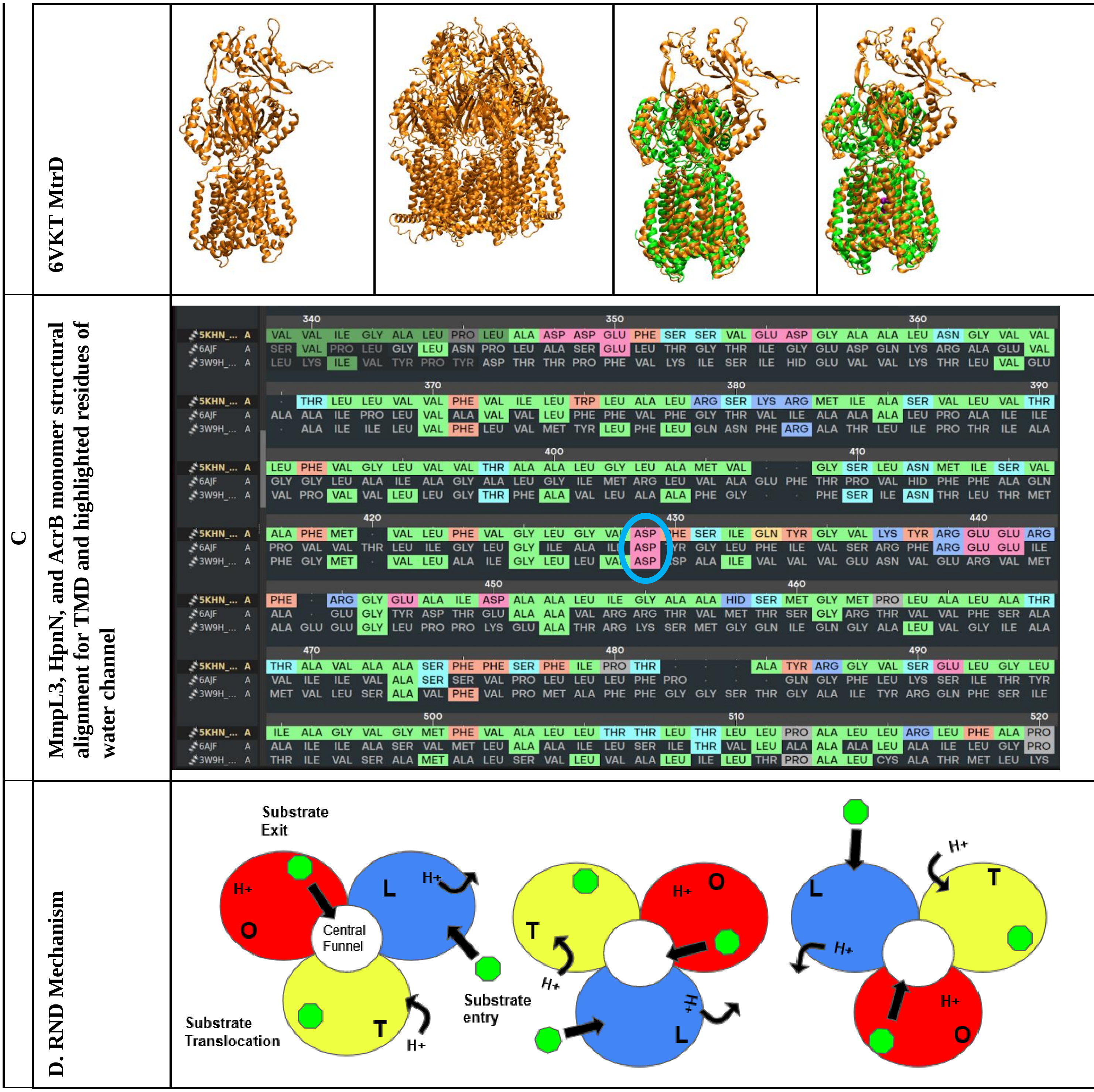

Figure S5 Structural comparison of MmpL3 (green) apo PDB:6AJF and Holo PDBID:6AJG with SQ109 aligned with RND transporters **A.** HpnN PDBID:5KHN(cyan). **B.** AcrB PDBID:3W9H(red), MexB PDBID:3W9I(yellow), MtrD PDBID: 6VKT(orange), and **C.** Sequence comparison of HpnN (PDBID:5KHN) dimer and AcrB (PDBID:3W9H) trimer, and MmpL3 (PDBID:6AJF) monomeric subunits for Trans-Membrane Domain (TMD). Conserved Asp residues shown with blue circle HpnN

Asp 344, AcrB Asp 407, and MmpL3 Asp 256 **D.** Proposed mechanism of O/T/L mechanism of RND transporters.

	Crystal Structure	Homology model		Crystal Structure	Homology model
A p o - f o r m	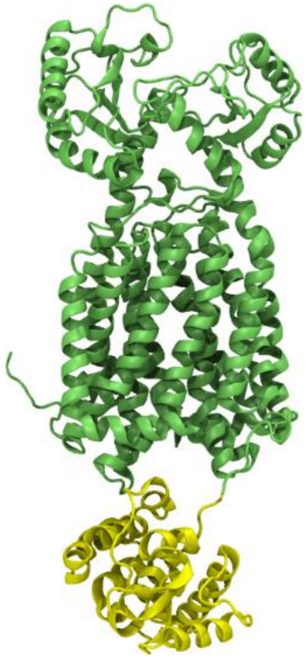	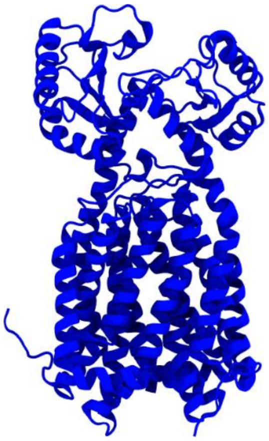	H o l o - f o r m	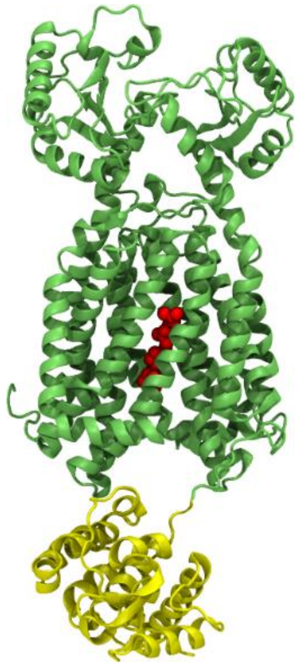	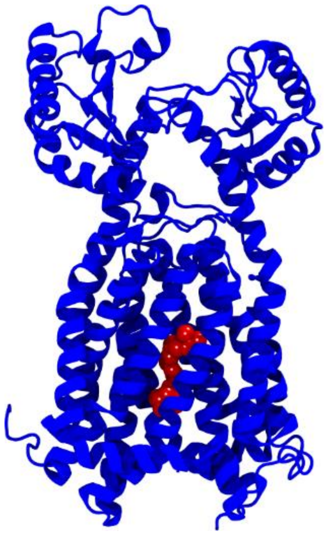

Figure S6. Comparison of MmpL3 apo-form structure (PDB ID: 6AJF) and MmpL3-SQ109 (red) holo-form structure (PDB ID: 6AJG) with the prepared homology models. The T4 lysozyme (yellow) is treated as an artifact; it was included in the crystal structure but removed to generate the homology model.

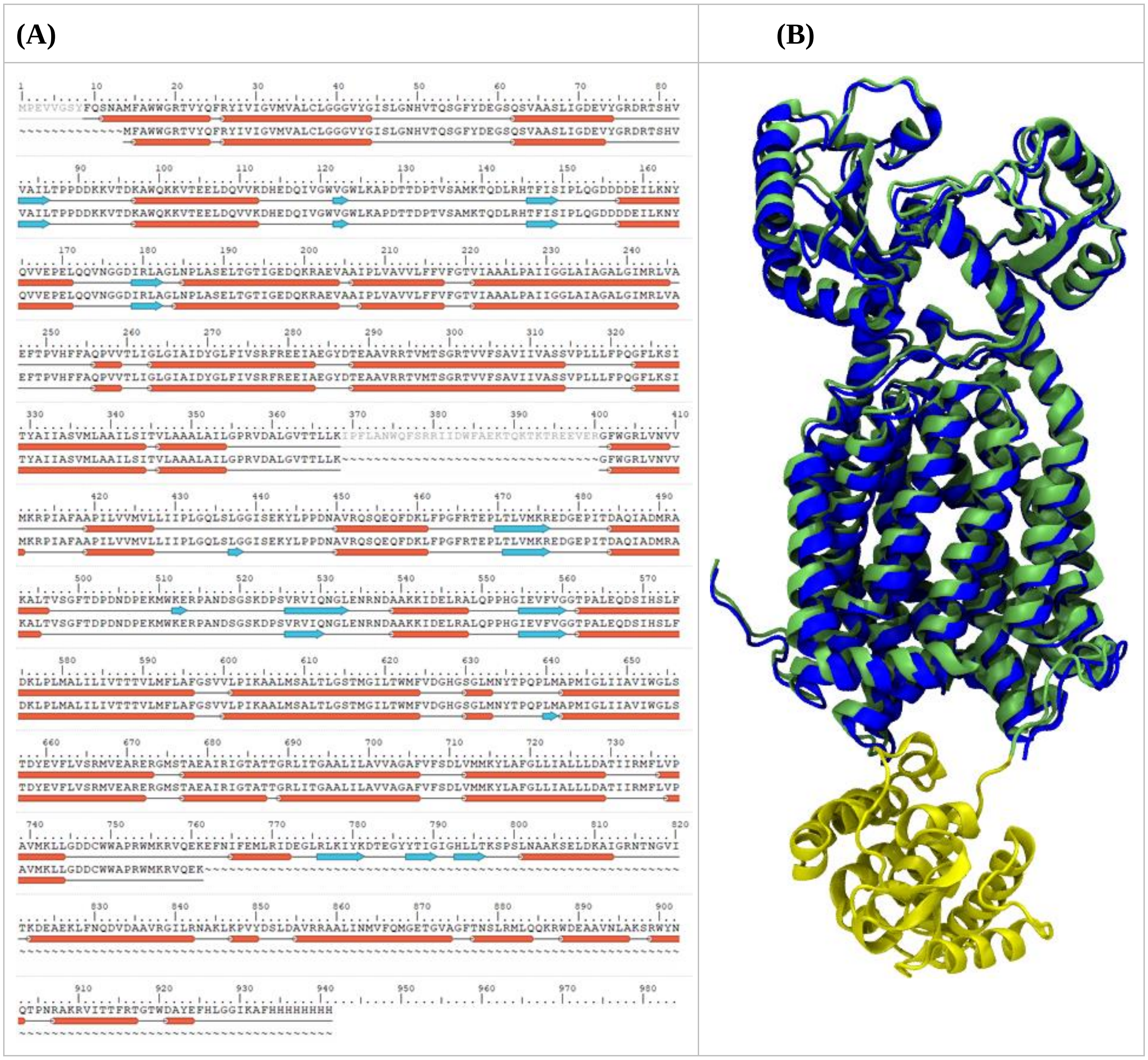

Figure S7. (A) Sequence alignment and (B) superimposition of the *M. smegmatis* MmpL3 apo-form crystal structure (PDB ID: 6AJF) (green) with the prepared homology model (blue). The top and bottom sequences in A) is the crystal structure and homology model, respectively. The T4 lysozyme (yellow) in B) is treated as an artifact; it was included in the crystal structure but removed to generate the homology model.

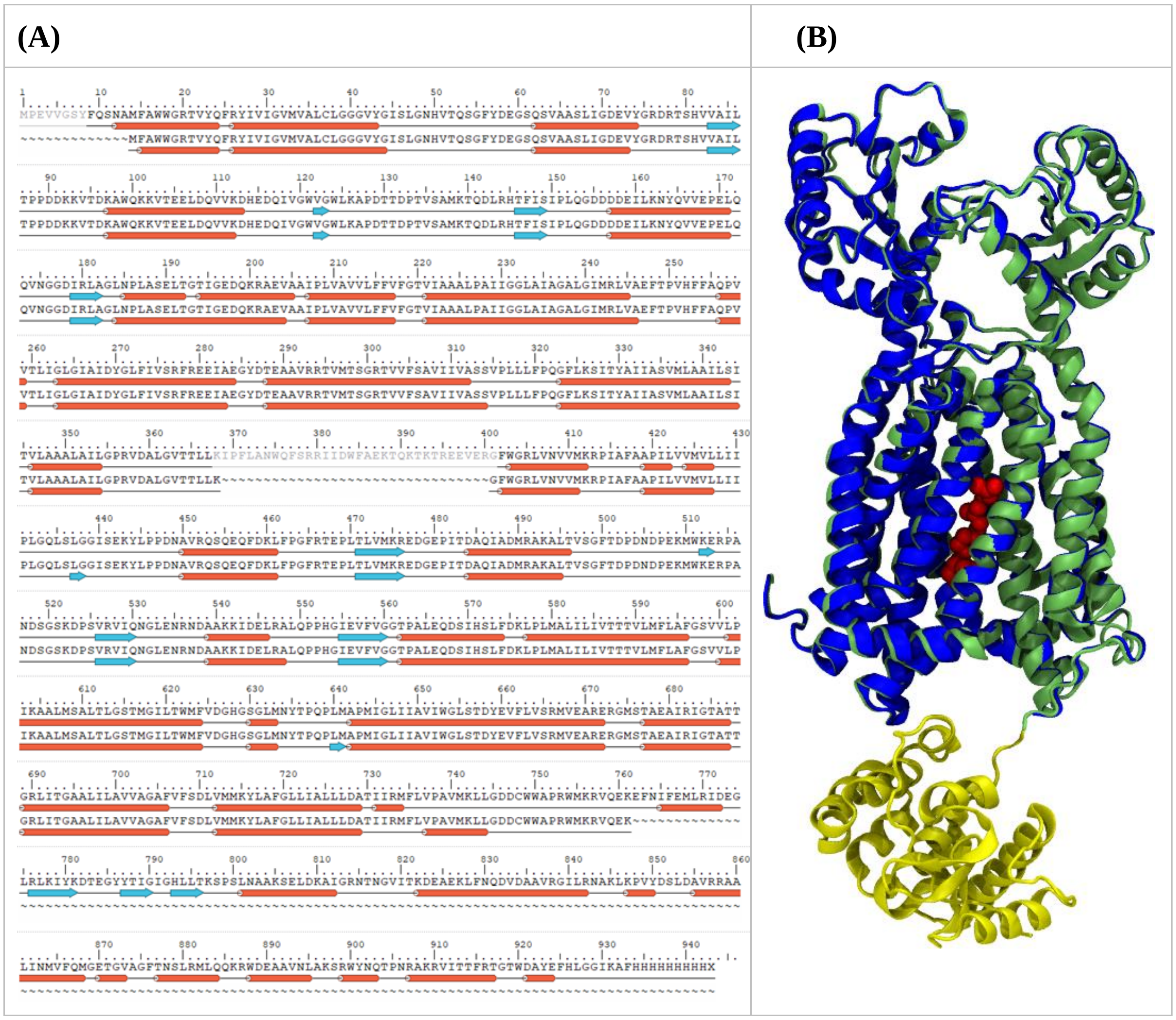

Figure S8 (A) Sequence alignment and (B) superimposition of the *M. smegmatis* MmpL3 complex crystal structure (PDB ID: 6AJG) (green) with the prepared homology model (blue). The top and bottom sequences in A) is the crystal structure and homology model, respectively. The T4 lysosome (yellow) in B) is treated as an artifact; it was included in the crystal structure but removed to generate the homology model.

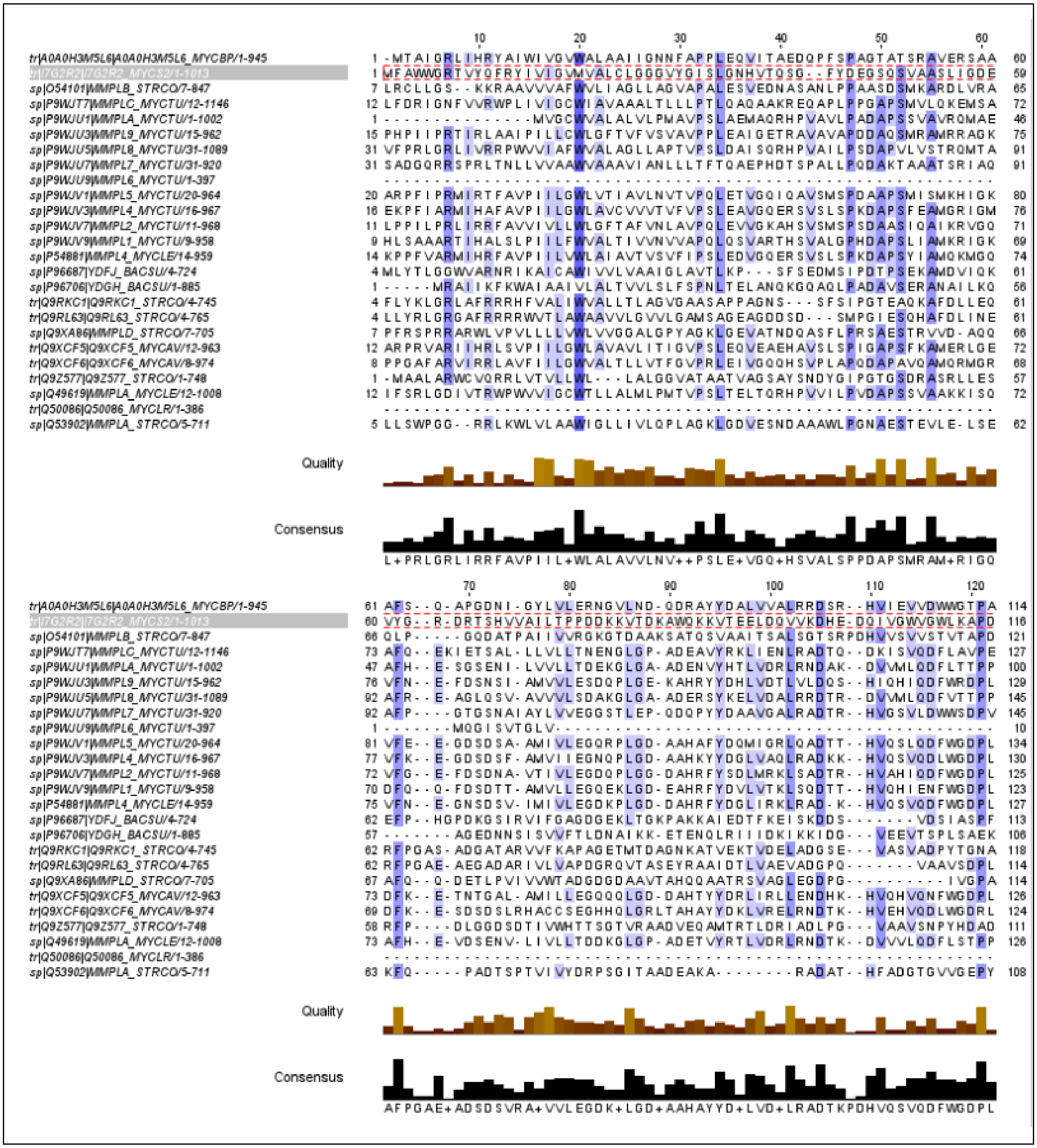
Quality
Consensus
L+PRLGRLIRRFAVPIIL+WLALAVVLNV++PSLE+VGQ+HSVALSPPDAPSMRAM+RIGQ
Quality
Consensus
AFPGAE+ADSDSVRA+VVLEGDK+LGD+AAHAYYD+LVD+LRADTKPDHVQSVQDFWGDPL

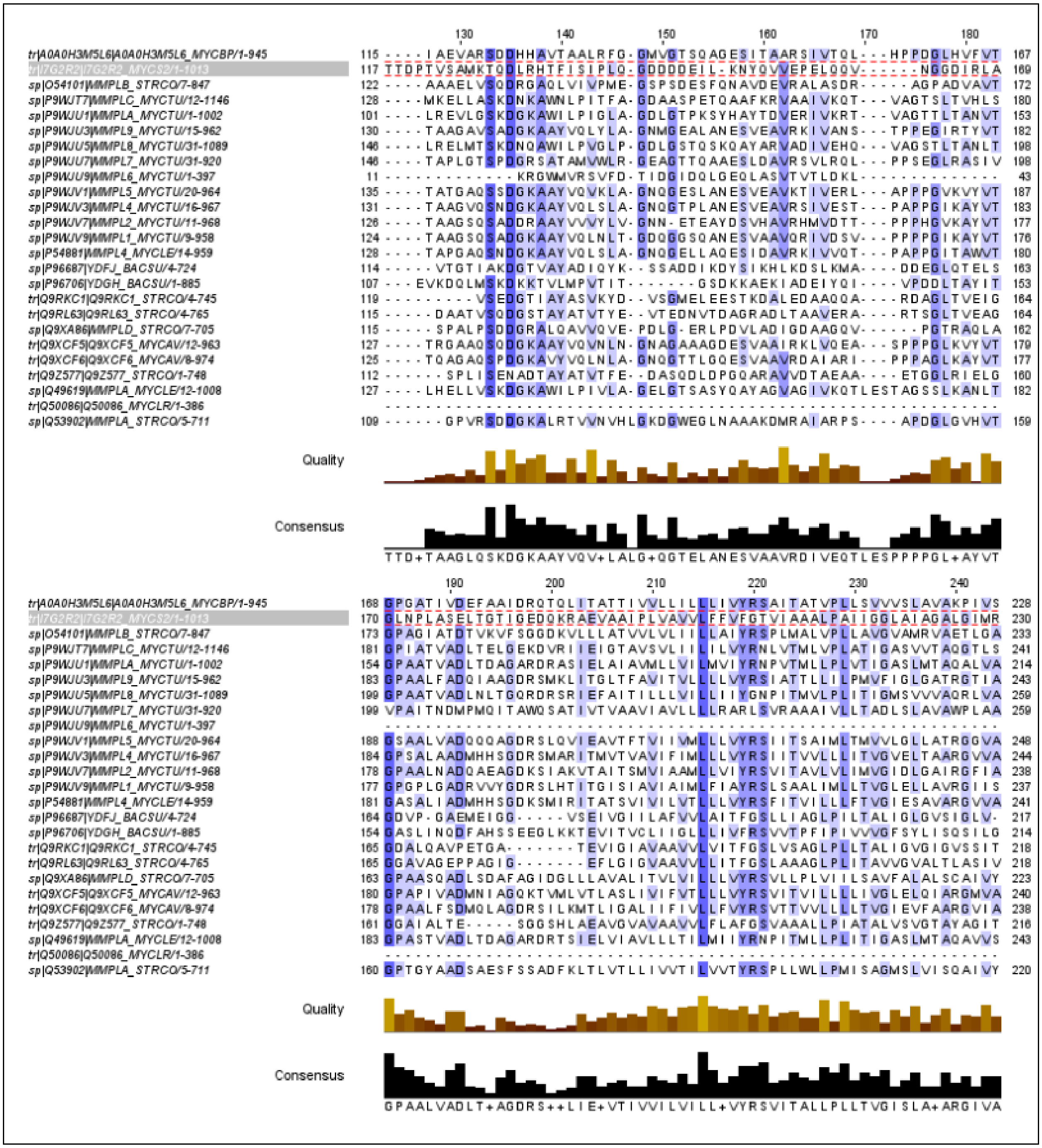

tr|A0A0H3M5L6|A0A0H3M5L6_MYCBP/1-945
tr|I7G2R2|I7G2R2_MYCS2/1-1013
sp|O54101|MMPLB_STRCO/7-847
sp|P9WJT7|MMPLC_MYCTU/12-1146
sp|P9WJU1|MMPLA_MYCTU/1-1002
sp|P9WJU3|MMPL9_MYCTU/15-962
sp|P9WJU5|MMPL8_MYCTU/31-1089
sp|P9WJU7|MMPL7_MYCTU/31-920
sp|P9WJU9|MMPL6_MYCTU/1-397
sp|P9WJV1|MMPL5_MYCTU/20-964
sp|P9WJV3|MMPL4_MYCTU/16-967
sp|P9WJV7|MMPL2_MYCTU/11-968
sp|P9WJV9|MMPL1_MYCTU/9-958
sp|P54881|MMPL4_MYCLE/14-959
sp|P96687|YDFJ_BACSU/4-724
sp|P96706|YDGH_BACSU/1-885
tr|Q9RKC1|Q9RKC1_STRCO/4-745
tr|Q9RL63|Q9RL63_STRCO/4-765
sp|Q9XA86|MMPLD_STRCO/7-705
tr|Q9XCF5|Q9XCF5_MYCAV/12-963
tr|Q9XCF6|Q9XCF6_MYCAV/8-974
tr|Q9Z577|Q9Z577_STRCO/1-748
sp|Q49619|MMPLA_MYCLE/12-1008
tr|Q50086|Q50086_MYCLR/1-386
sp|Q53902|MMPLA_STRCO/5-711
Quality
Consensus
TTD+TAAGLQSKDGKAAYVQV+LALG+QGTELANESVAAVRDIVEQTLESPPPPGL+AYVT
Quality
Consensus
GPAALVADLT+AGDRS++LIE+VTIVVILVILL+VYRSVITALLPLLTVGISLA+ARGIVA

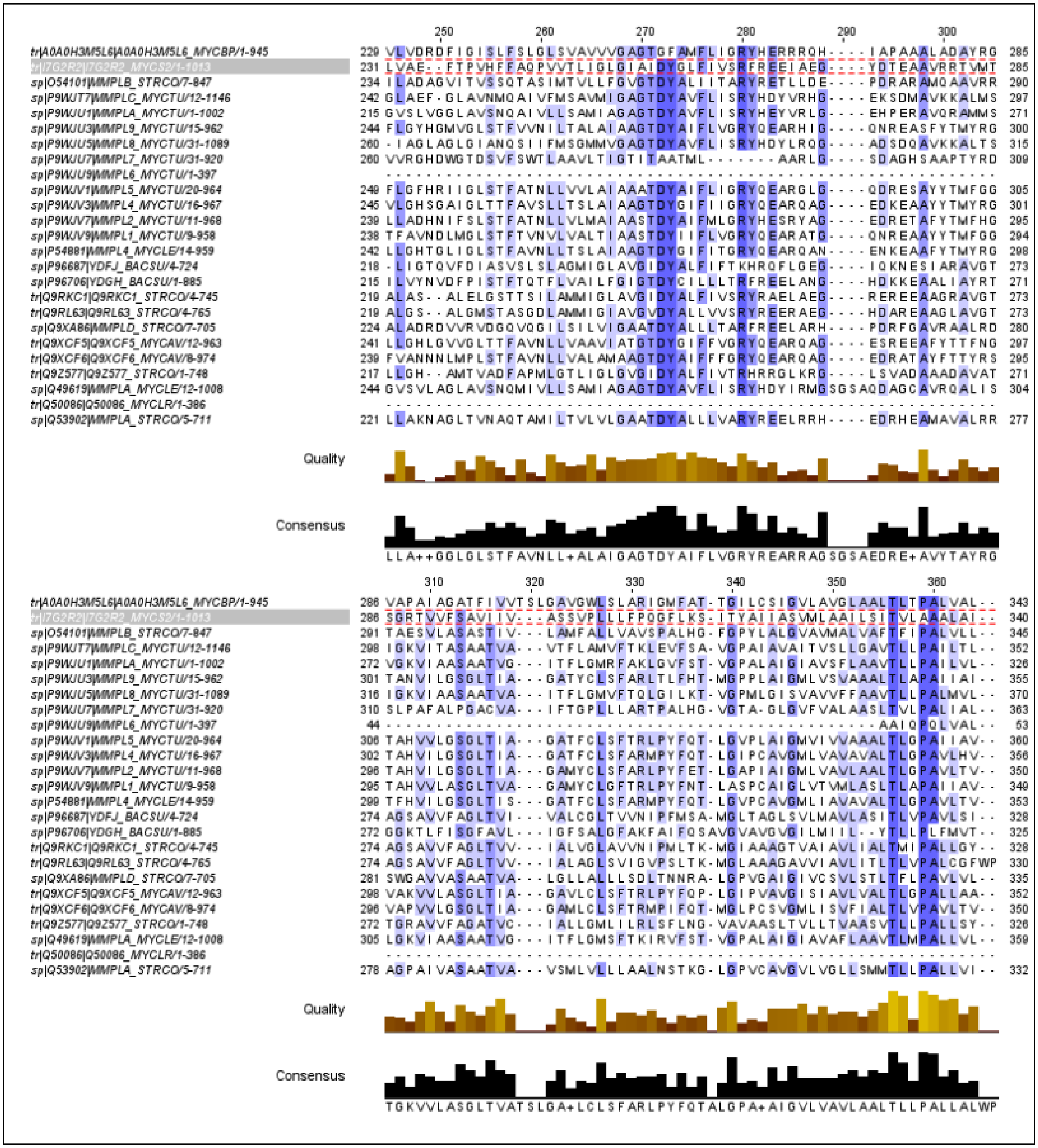

250 260 270 280 290 300
tr|A0A0H3M5L6|A0A0H3M5L6_MYCBP/1-945
tr|I7G2R2|I7G2R2_MYCS2/1-1013
sp|O54101|MMPLB_STRCO/7-847
sp|P9WJT7|MMPLC_MYCTU/12-1146
sp|P9WJU1|MMPLA_MYCTU/1-1002
sp|P9WJU3|MMPL9_MYCTU/15-962
sp|P9WJU5|MMPL8_MYCTU/31-1089
sp|P9WJU7|MMPL7_MYCTU/31-920
sp|P9WJU9|MMPL6_MYCTU/1-397
sp|P9WJV1|MMPL5_MYCTU/20-964
sp|P9WJV3|MMPL4_MYCTU/16-967
sp|P9WJV7|MMPL2_MYCTU/11-968
sp|P9WJV9|MMPL1_MYCTU/9-958
sp|P54881|MMPL4_MYCLE/14-959
sp|P96687|YDFJ_BACSU/4-724
sp|P96706|YDGH_BACSU/1-885
tr|Q9RKC1|Q9RKC1_STRCO/4-745
tr|Q9RL63|Q9RL63_STRCO/4-765
sp|Q9XA86|MMPLD_STRCO/7-705
tr|Q9XCF5|Q9XCF5_MYCAV/12-963
tr|Q9XCF6|Q9XCF6_MYCAV/8-974
tr|Q9Z577|Q9Z577_STRCO/1-748
sp|Q49619|MMPLA_MYCLE/12-1008
tr|Q50086|Q50086_MYCLR/1-386
sp|Q53902|MMPLA_STRCO/5-711
Quality
Consensus
LLA++GGLGLSTFAVNLL+ALAIGAGTDYAIFLVGRYREARRAGSGSAEDRE+AVYTAYRG
310 320 330 340 350 360
Quality
Consensus
TGKVVLASGLTVATSLGA+LCLSFARLPYFQTALGPA+AIGVLVAVLAALTLLPALLALWP

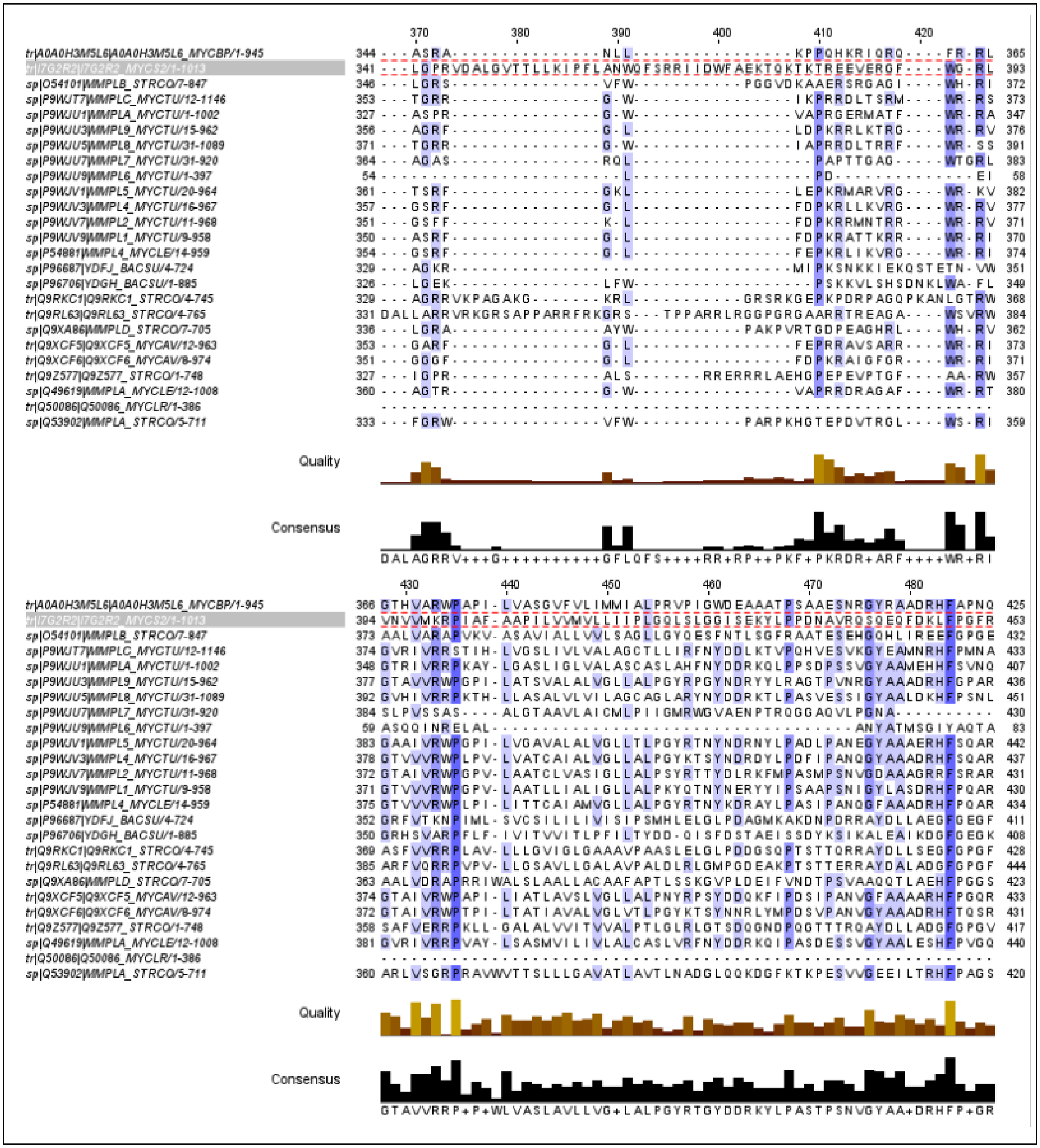

tr|A0A0H3M5L6|A0A0H3M5L6_MYCBP/1-945
tr|I7G2R2|I7G2R2_MYCS2/1-1013
sp|O54101|MMPLB_STRCO/7-847
sp|P9WJT7|MMPLC_MYCTU/12-1146
sp|P9WJU1|MMPLA_MYCTU/1-1002
sp|P9WJU3|MMPL9_MYCTU/15-962
sp|P9WJU5|MMPL8_MYCTU/31-1089
sp|P9WJU7|MMPL7_MYCTU/31-920
sp|P9WJU9|MMPL6_MYCTU/1-397
sp|P9WJV1|MMPL5_MYCTU/20-964
sp|P9WJV3|MMPL4_MYCTU/16-967
sp|P9WJV7|MMPL2_MYCTU/11-968
sp|P9WJV9|MMPL1_MYCTU/9-958
sp|P54881|MMPL4_MYCLE/14-959
sp|P96687|YDFJ_BACSU/4-724
sp|P96706|YDGH_BACSU/1-885
tr|Q9RKC1|Q9RKC1_STRCO/4-745
tr|Q9RL63|Q9RL63_STRCO/4-765
sp|Q9XA86|MMPLD_STRCO/7-705
tr|Q9XCF5|Q9XCF5_MYCAV/12-963
tr|Q9XCF6|Q9XCF6_MYCAV/8-974
tr|Q9Z577|Q9Z577_STRCO/1-748
sp|Q49619|MMPLA_MYCLE/12-1008
tr|Q50086|Q50086_MYCLR/1-386
sp|Q53902|MMPLA_STRCO/5-711
Quality
Consensus
DALAGRRV+++G++++++++++GFLQFS++++RR+RP++PKF+PKRDR+ARF++++WR+RI
Quality
Consensus
GTAVVRRP+P+WLVASLAVLLVG+LALPGYRTGYDDRKYLPASTPSNVGYAA+DRHFP+GR

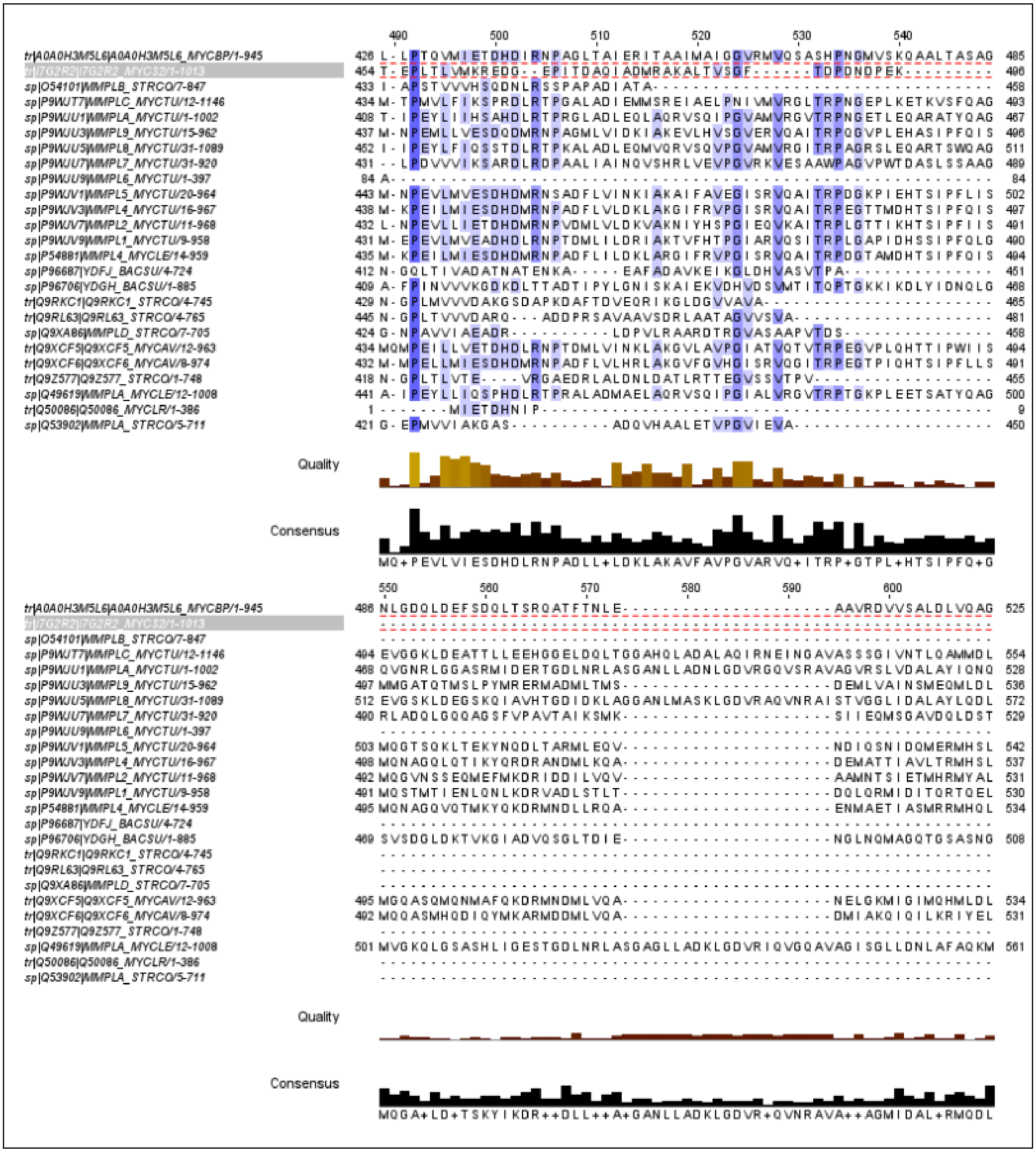
490 500 510 520 530 540
tr|A0A0H3M5L6|A0A0H3M5L6_MYCBP/1-945
tr|I7G2R2|I7G2R2_MYCS2/1-1013
sp|O54101|MMPLB_STRCO/7-847
sp|P9WJT7|MMPLC_MYCTU/12-1146
sp|P9WJU1|MMPLA_MYCTU/1-1002
sp|P9WJU3|MMPL9_MYCTU/15-962
sp|P9WJU5|MMPL8_MYCTU/31-1089
sp|P9WJU7|MMPL7_MYCTU/31-920
sp|P9WJU9|MMPL6_MYCTU/1-397
sp|P9WJV1|MMPL5_MYCTU/20-964
sp|P9WJV3|MMPL4_MYCTU/16-967
sp|P9WJV7|MMPL2_MYCTU/11-968
sp|P9WJV9|MMPL1_MYCTU/9-958
sp|P54881|MMPL4_MYCLE/14-959
sp|P96687|YDFJ_BACSU/4-724
sp|P96706|YDGH_BACSU/1-885
tr|Q9RKC1|Q9RKC1_STRCO/4-745
tr|Q9RL63|Q9RL63_STRCO/4-765
sp|Q9XA86|MMPLD_STRCO/7-705
tr|Q9XCF5|Q9XCF5_MYCAV/12-963
tr|Q9XCF6|Q9XCF6_MYCAV/8-974
tr|Q9Z577|Q9Z577_STRCO/1-748
sp|Q49619|MMPLA_MYCLE/12-1008
tr|Q50086|Q50086_MYCLR/1-386
sp|Q53902|MMPLA_STRCO/5-711
Quality
Consensus
MQ+PEVLVIESDHDLRNPADLL+LDKLAKAVFAVPGVARVQ+ITRP+GTPL+HTSIPFQ+G
550 560 570 580 590 600
Quality
Consensus
MQGA+LD+TSKYIKDR++DLL++A+GANLLADKLGDVR+QVNRAVA++AGMIDAL+RMQDL

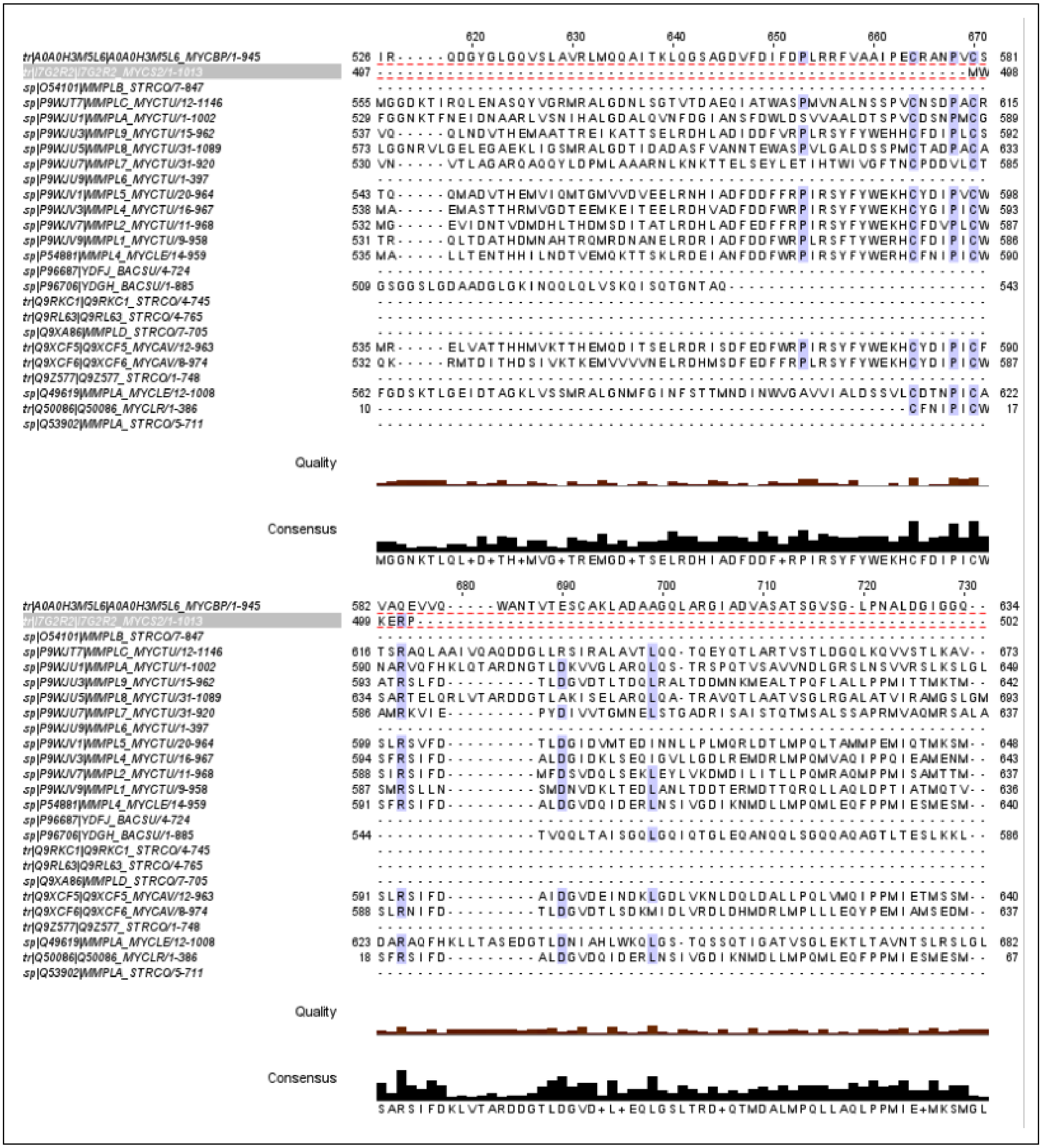
Quality
Consensus
MGGNKTLQL+D+TH+MVG+TREMGD+TSELRDHIADFDDF+RPIRSYFYWEKHCFDIPICW
Quality
Consensus
SARSIFDKLVTARDDGTLDGVD+L+EQLGSLTRD+QTMDALMPQLLAQLPPMIE+MKSMGL

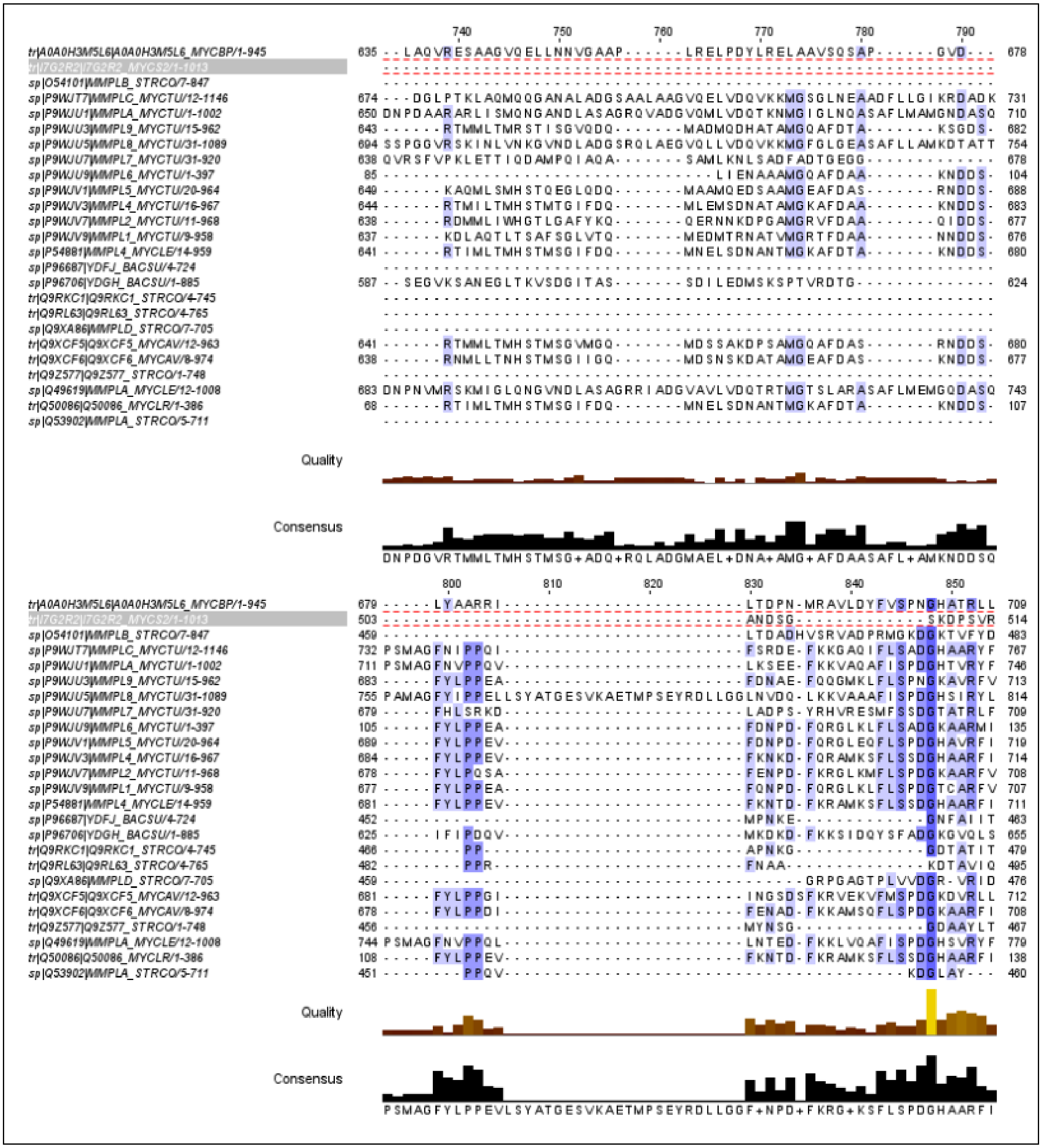

740
750
760
770
780
790
tr|A0A0H3M5L6|A0A0H3M5L6_MYCBP/1-945
sp|O54101|MMPLB_STRCO/7-847
sp|P9WJT7|MMPLC_MYCTU/12-1146
sp|P9WJU1|MMPLA_MYCTU/1-1002
sp|P9WJU3|MMPL9_MYCTU/15-962
sp|P9WJU5|MMPL8_MYCTU/31-1089
sp|P9WJU7|MMPL7_MYCTU/31-920
sp|P9WJU9|MMPL6_MYCTU/1-397
sp|P9WJV1|MMPL5_MYCTU/20-964
sp|P9WJV3|MMPL4_MYCTU/16-967
sp|P9WJV7|MMPL2_MYCTU/11-968
sp|P9WJV9|MMPL1_MYCTU/9-958
sp|P54881|MMPL4_MYCLE/14-959
sp|P96687|YDFJ_BACSU/4-724
sp|P96706|YDGH_BACSU/1-885
tr|Q9RKC1|Q9RKC1_STRCO/4-745
tr|Q9RL63|Q9RL63_STRCO/4-765
sp|Q9XA86|MMPLD_STRCO/7-705
tr|Q9XCF5|Q9XCF5_MYCAV/12-963
tr|Q9XCF6|Q9XCF6_MYCAV/8-974
tr|Q9Z577|Q9Z577_STRCO/1-748
sp|Q49619|MMPLA_MYCLE/12-1008
tr|Q50086|Q50086_MYCLR/1-386
sp|Q53902|MMPLA_STRCO/5-711
Quality
Consensus
DNPDGVRTMMLTMHSTMSG+ADQ+RQLADGMAEL+DNA+AMG+AFDAASAFL+AMKNDDSQ
800
810
820
830
840
850
Quality
Consensus
PSMAGFYLPPEVLSYATGESVKAETMPSEYRDLLGGF+NPD+FKRG+KSFLSPDGHAARFI

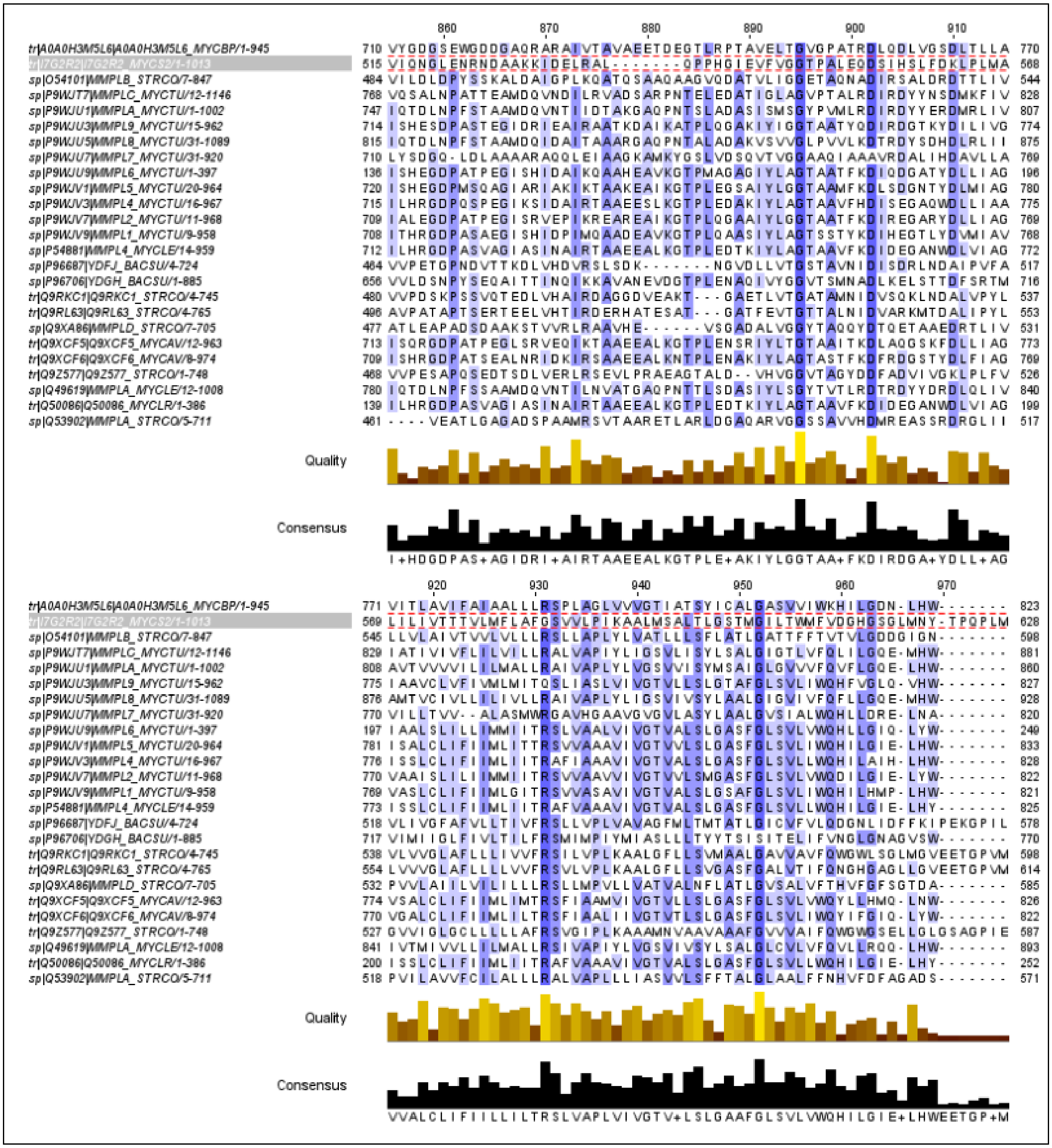

860
870
880
890
900
910
Quality
Consensus
920
930
940
950
960
970
Quality
Consensus

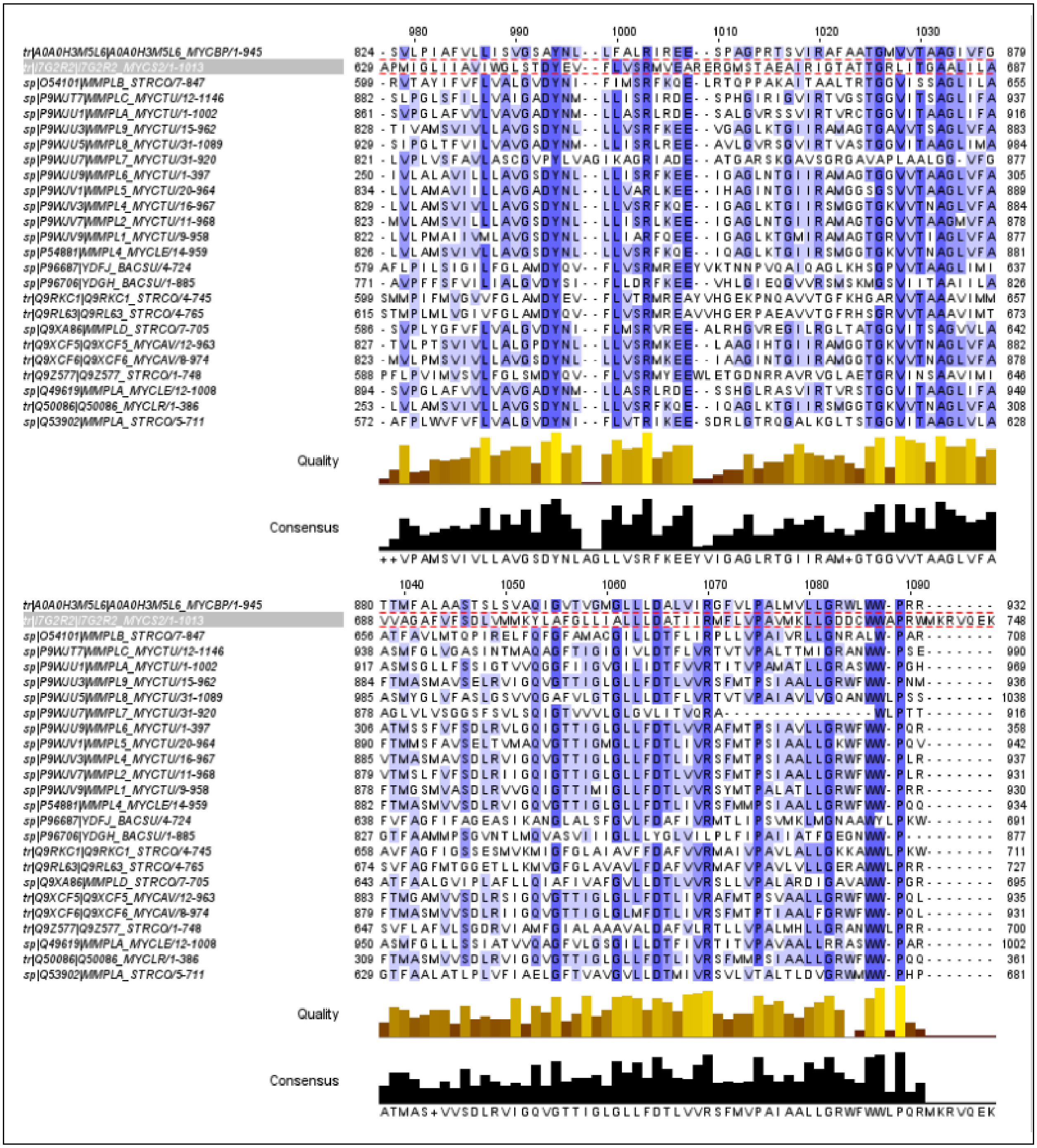

Figure S9. Multiple sequence alignment of MmpL family (PF.03176.15) obtained from pfam. The quality of conserved residues throughout the sequences are indicated with purple shading.

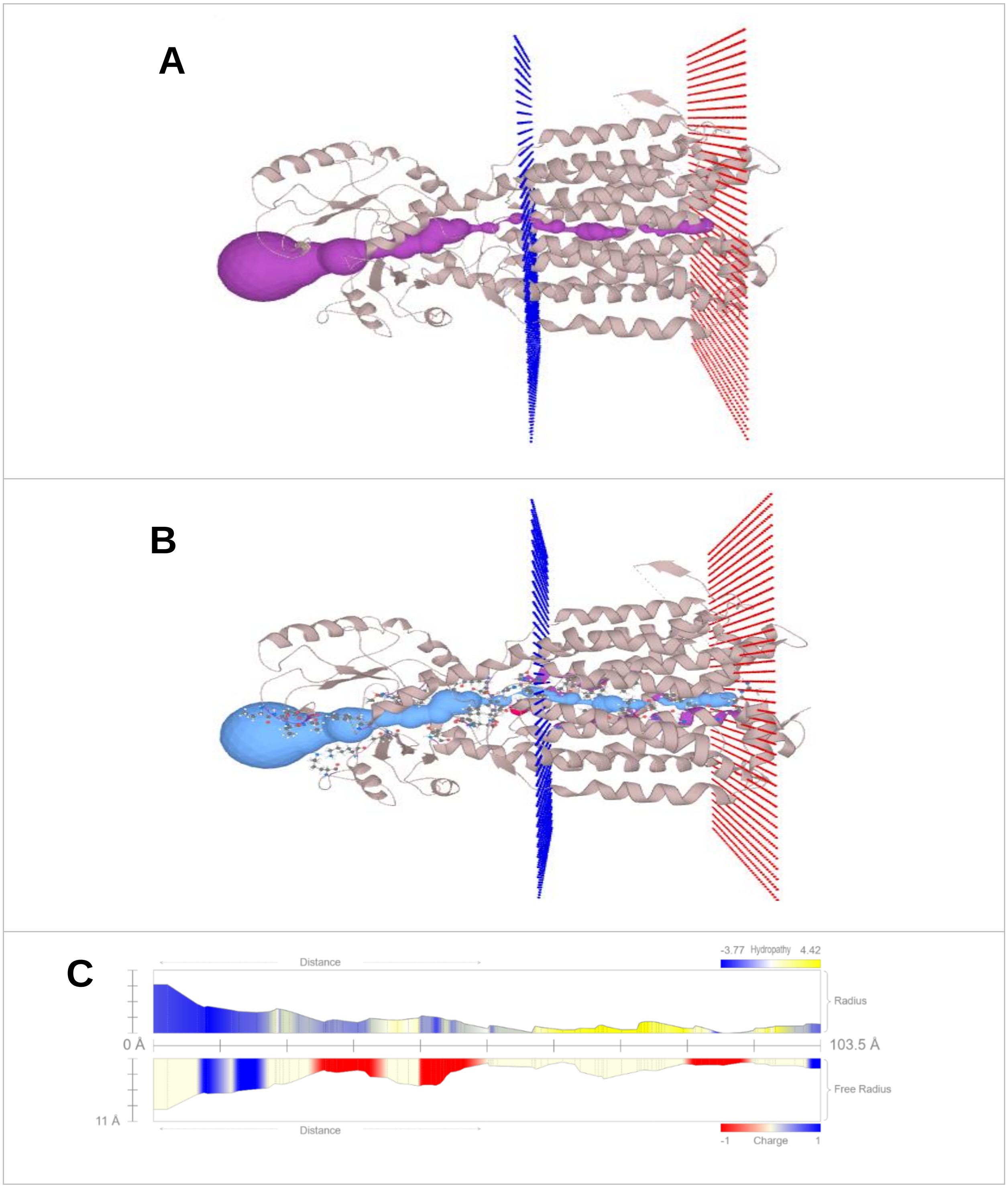

Figure S10. MOLEOnline analysis of the MmpL3 apo-form crystal structure (PDB ID:6AJF) showing the proton pathway in the N-terminal domain and the substrate transport pathway in the C-terminal domain. (**A**) MMPL3 Apo channel analysis showing overall possible channel through the protein leading through Tyr646 and Asp256. (**B**) MmpL3 apo-form with key residues showing proton and substrate pathways. (**C**) Channel properties of MmpL3 Apo-form showing hydrophobicity and radius on top and

charge and free radius on the bottom. Red and blue planes represent intracellular and periplasmic domains, respectively.

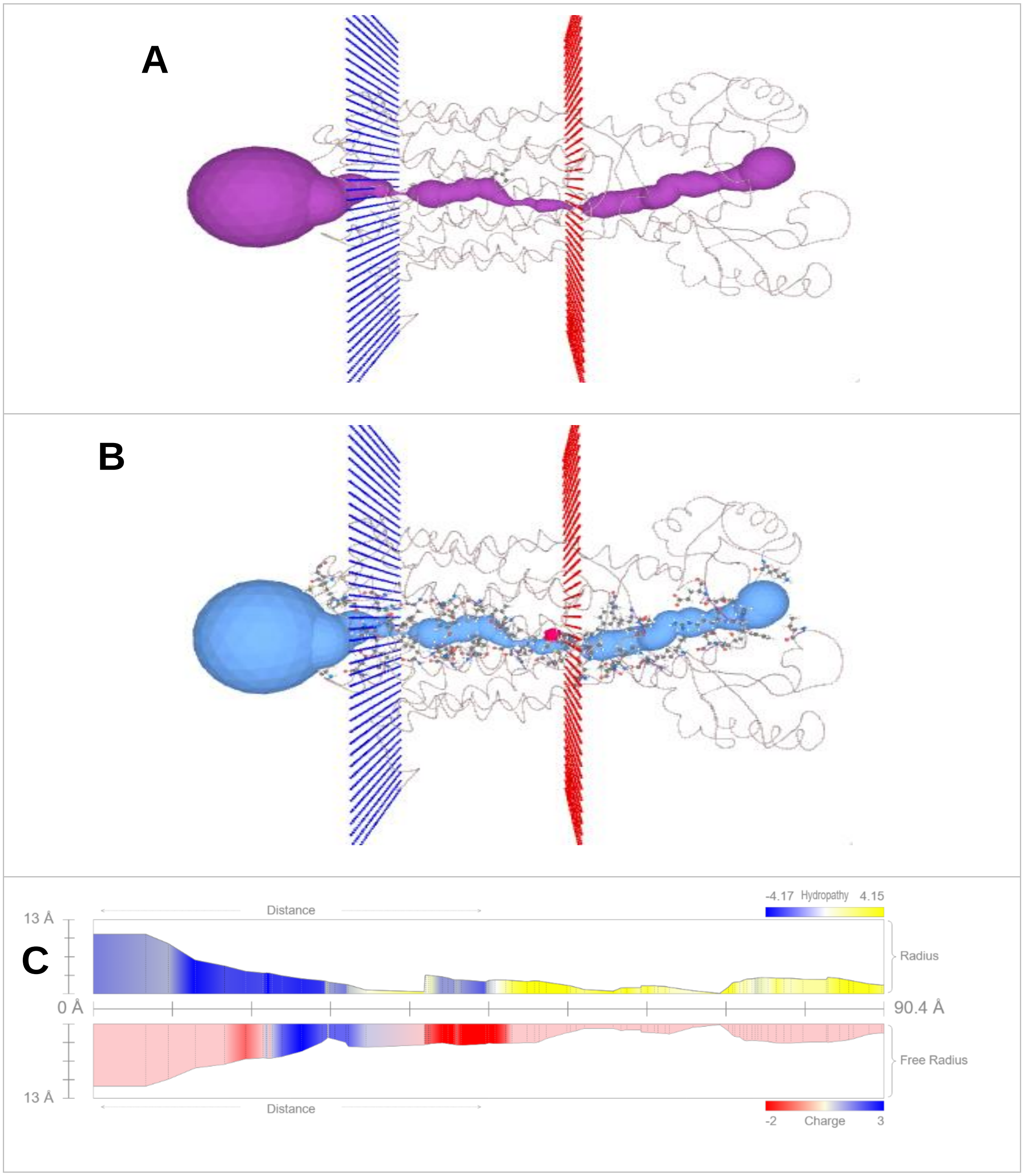

Figure S11. MOLEOnline analysis of the MmpL3 holo-form crystal structure (PDB ID: 6AJG). Inhibitor bound state will show inhibitor interaction at key residues. (**A**) MmpL3 holo-form showing the proton pathway with key residues F 649 and F 260. (**B**) MmpL3 holo-form showing the proton pathway through the pore with no key residues. (**C**) Channel properties of holo-form channel.

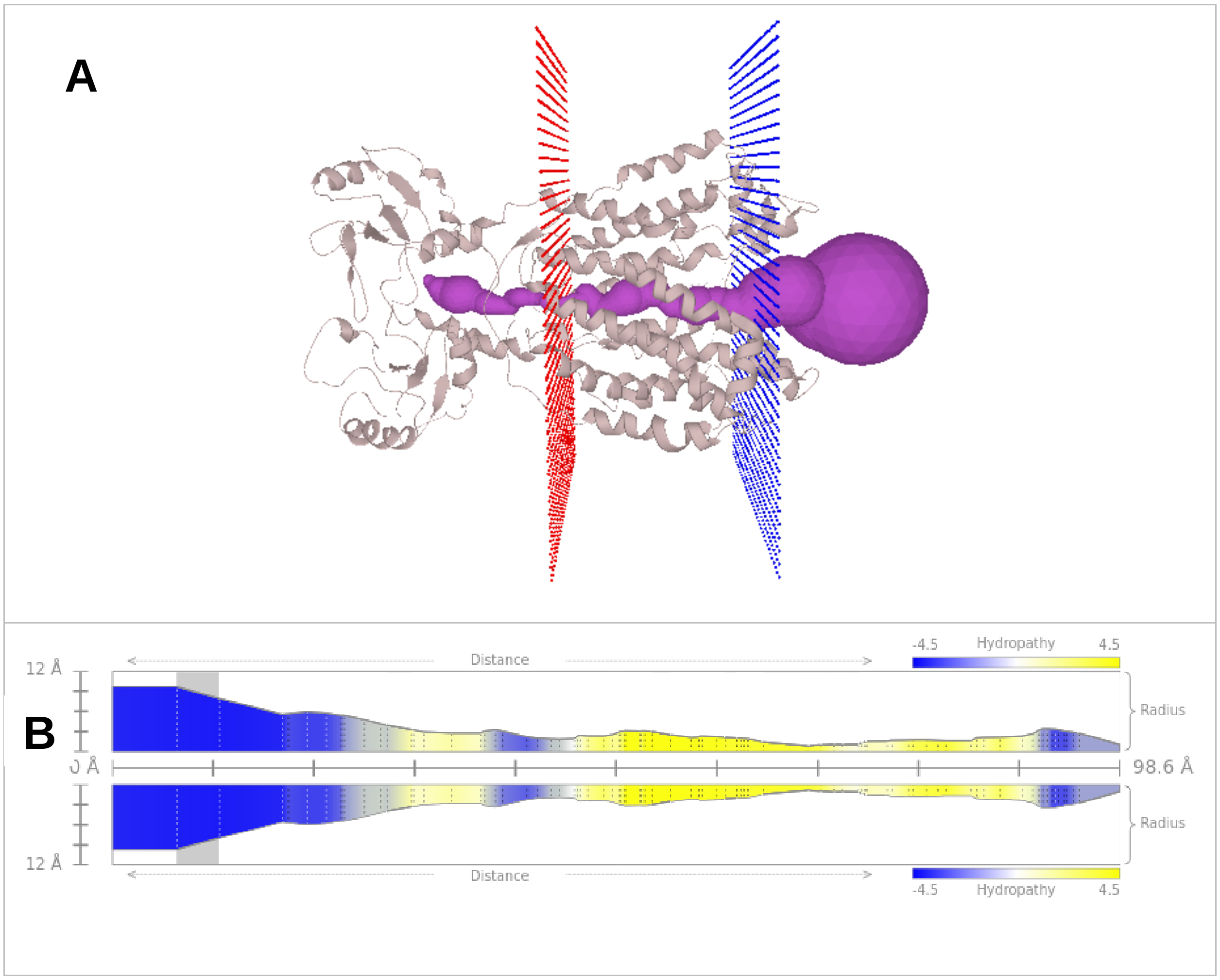

Figure S12. MOLEOnline analysis of the most abundant MmpL3 holo-form MD structure (from PDB ID: 6AJG). (**A**) MmpL3 holo-form showing the proton pathway through the pore with no key residues. (**B**) Channel properties of holo-form channel.

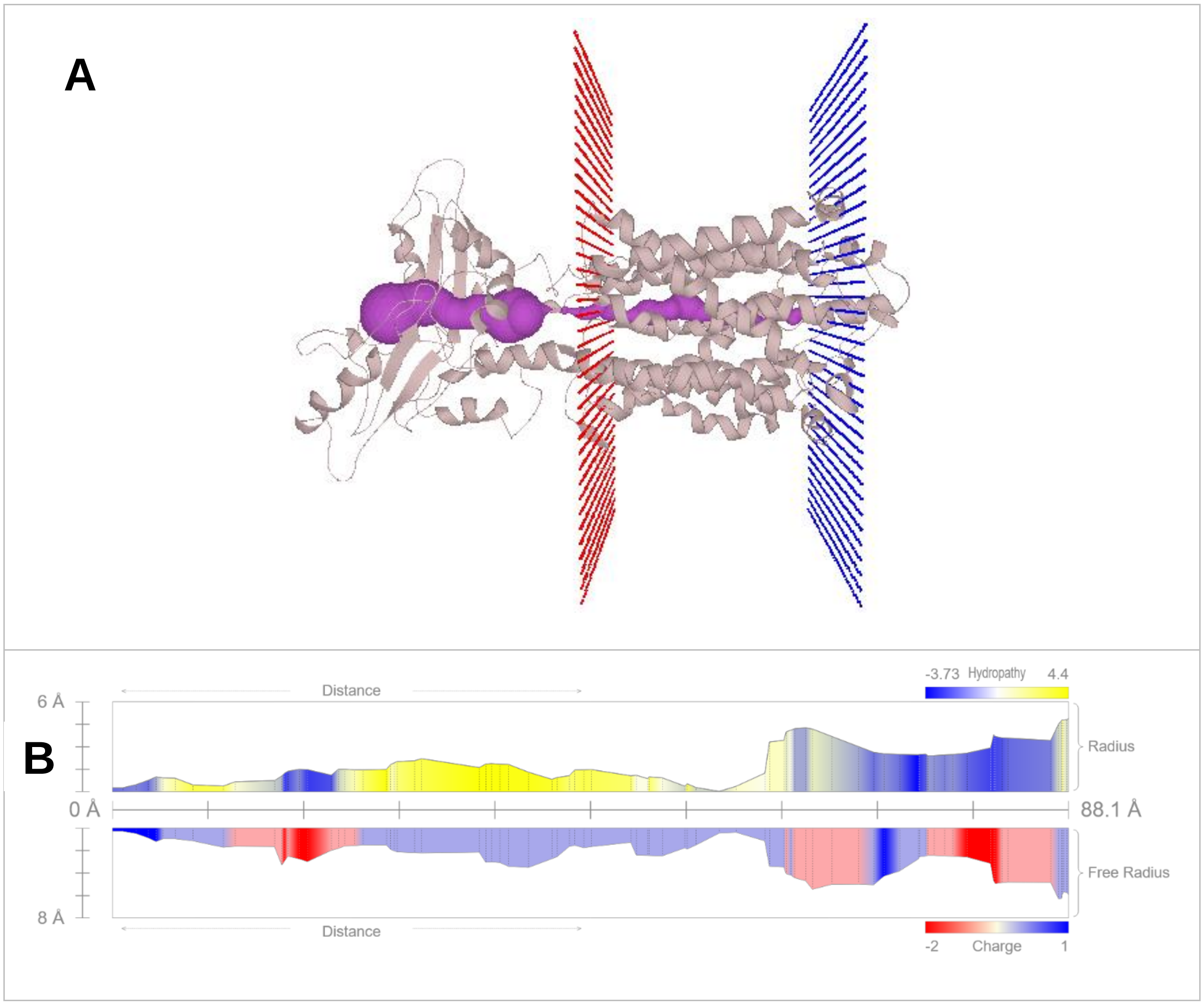

Figure S13. MOLEOnline analysis of the most abundant MmpL3 apo-form MD structure (from PDB ID: 6AJF). (**A**) MmpL3 apo-form showing the proton pathway through the pore with no key residues. (**B**) Channel properties of apo-form channel.

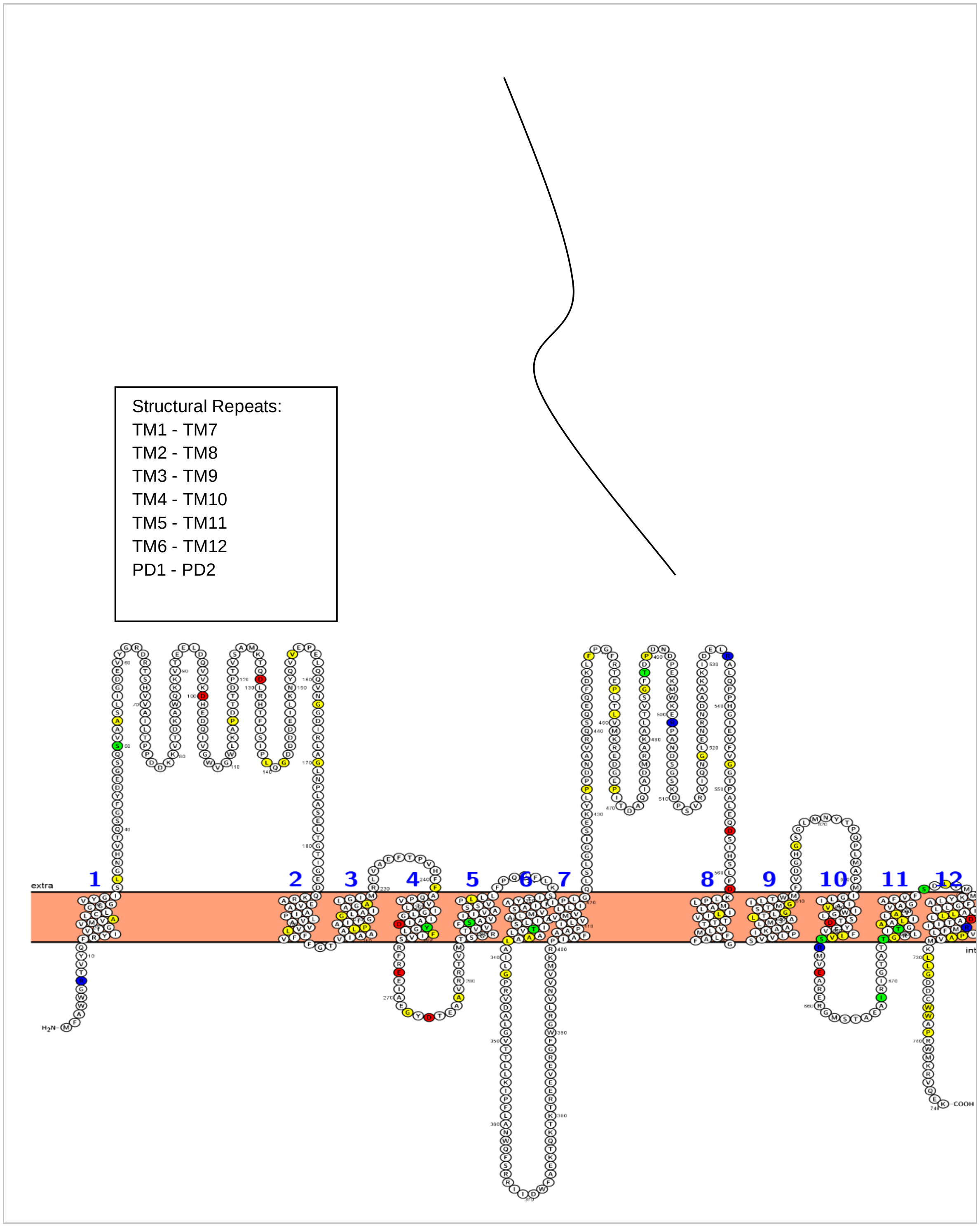

Figure S14. Snake plot of three-dimensional structure of MmpL3 with conserved residues displayed with a cutoff of 50% sequence identity. Residues are colored as acidic (red), basic (blue), polar (green) and non-polar (yellow).

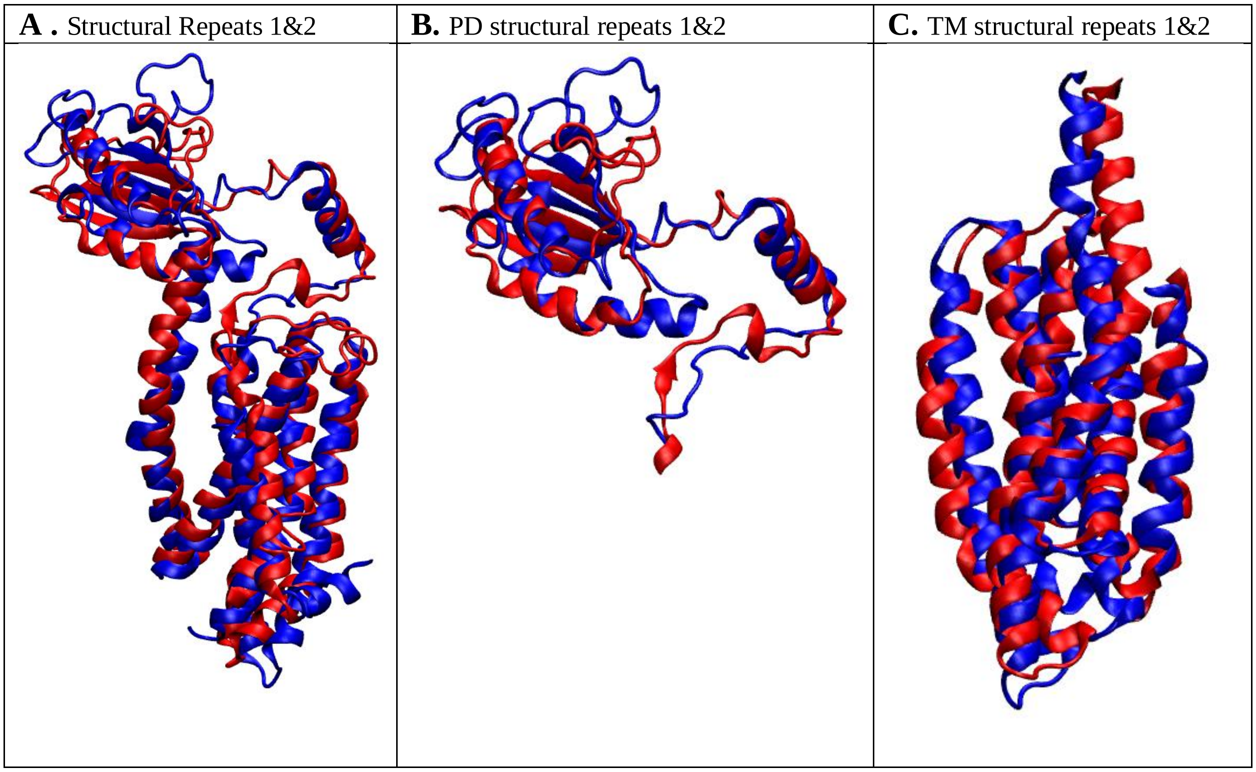

Figure S15 Structural repeat comparison. A. Overall prepared crystal structure split at residues 1-344(blue) and 388-730(red). B. Porter domain repeats split from PD1 residues 35-17(blue) and PD2 547-583 (red). C. Transmembrane domain repeats TMD1 residues (1-34, 170-344) (red) and TMD2 residues (388-420, 548-584) (red)

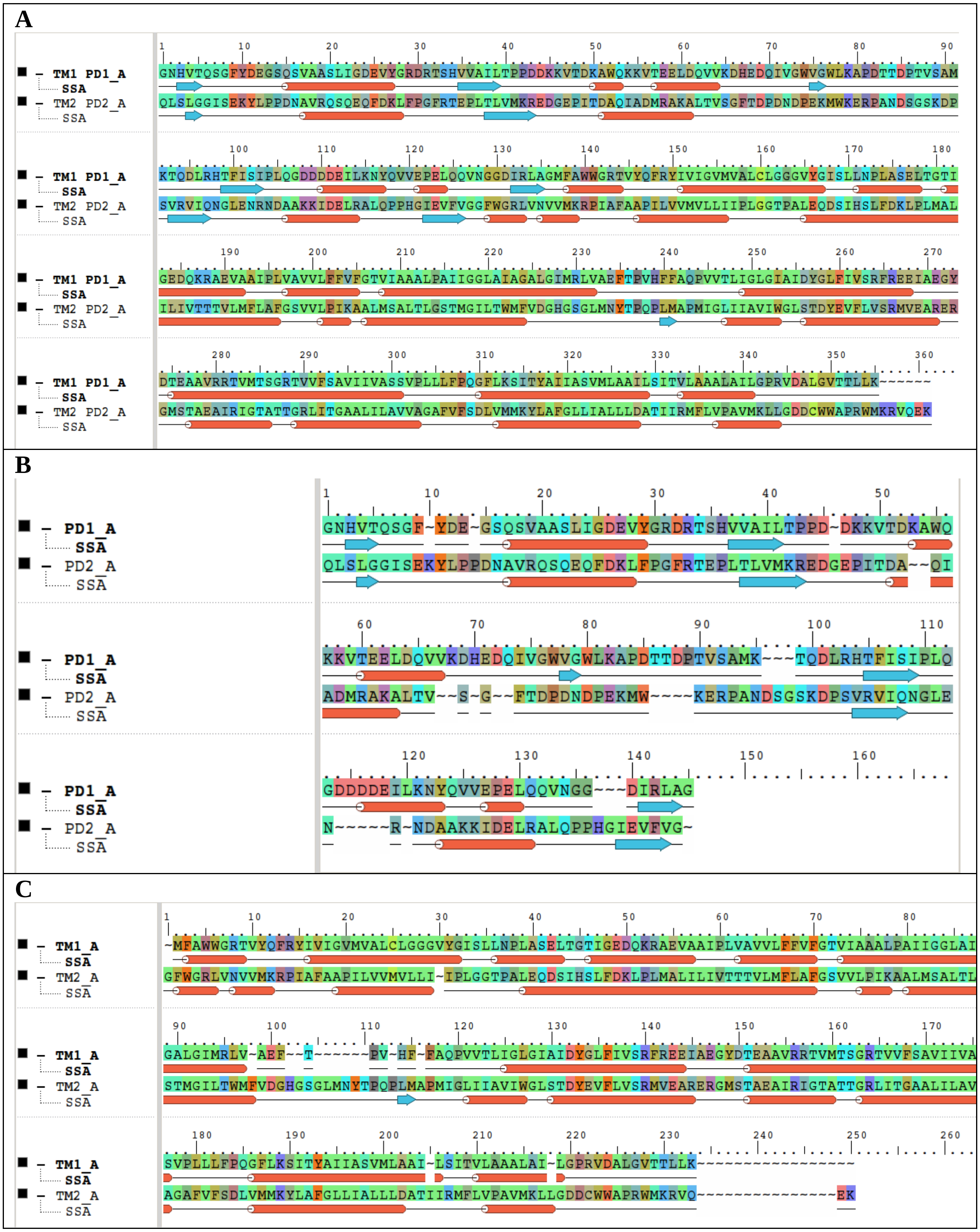

Figure S16 Sequence alignment of the two structural repeats. **A:** for overall repeats 1-344 and 388-730. **B**: Porter domain repeats, PD1 residues 35-17 and PD2 547-583. **C:** TM domain repeats TMD1 residues

(1-34, 170-344) and TMD2 residues (388-420, 548-584). Secondary structure for each repeat is shown under the sequence.

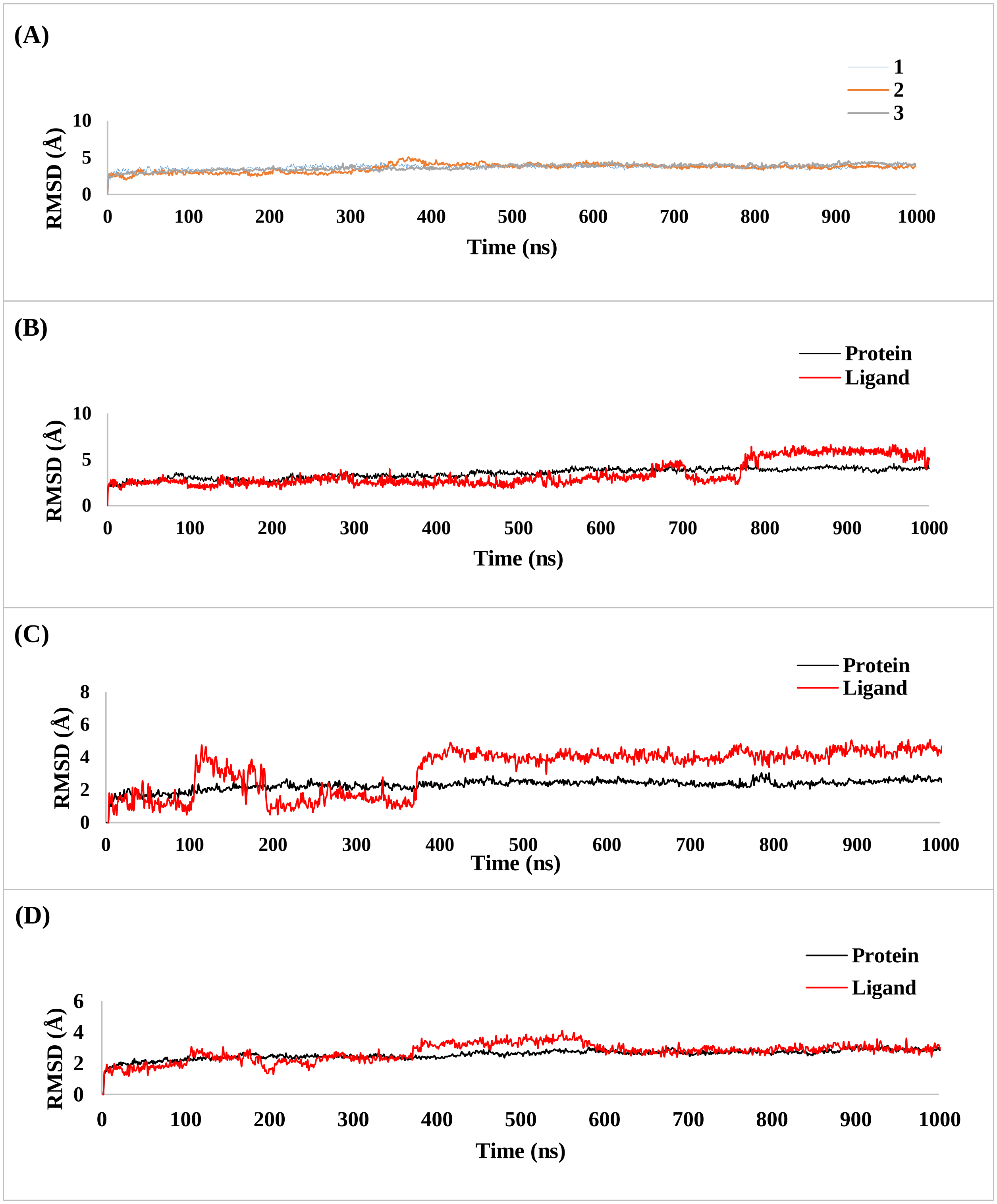

Figure S17. **(A)** Protein RMSD of the apo-form crystal structure (PDB ID: 6AJF) from each of the three MD simulations. **(B, C, & D)** Protein-ligand RMSD diagrams for SQ109-MmpL3 complexes (PDB ID: 6AJG) for each of the three MD simulations.

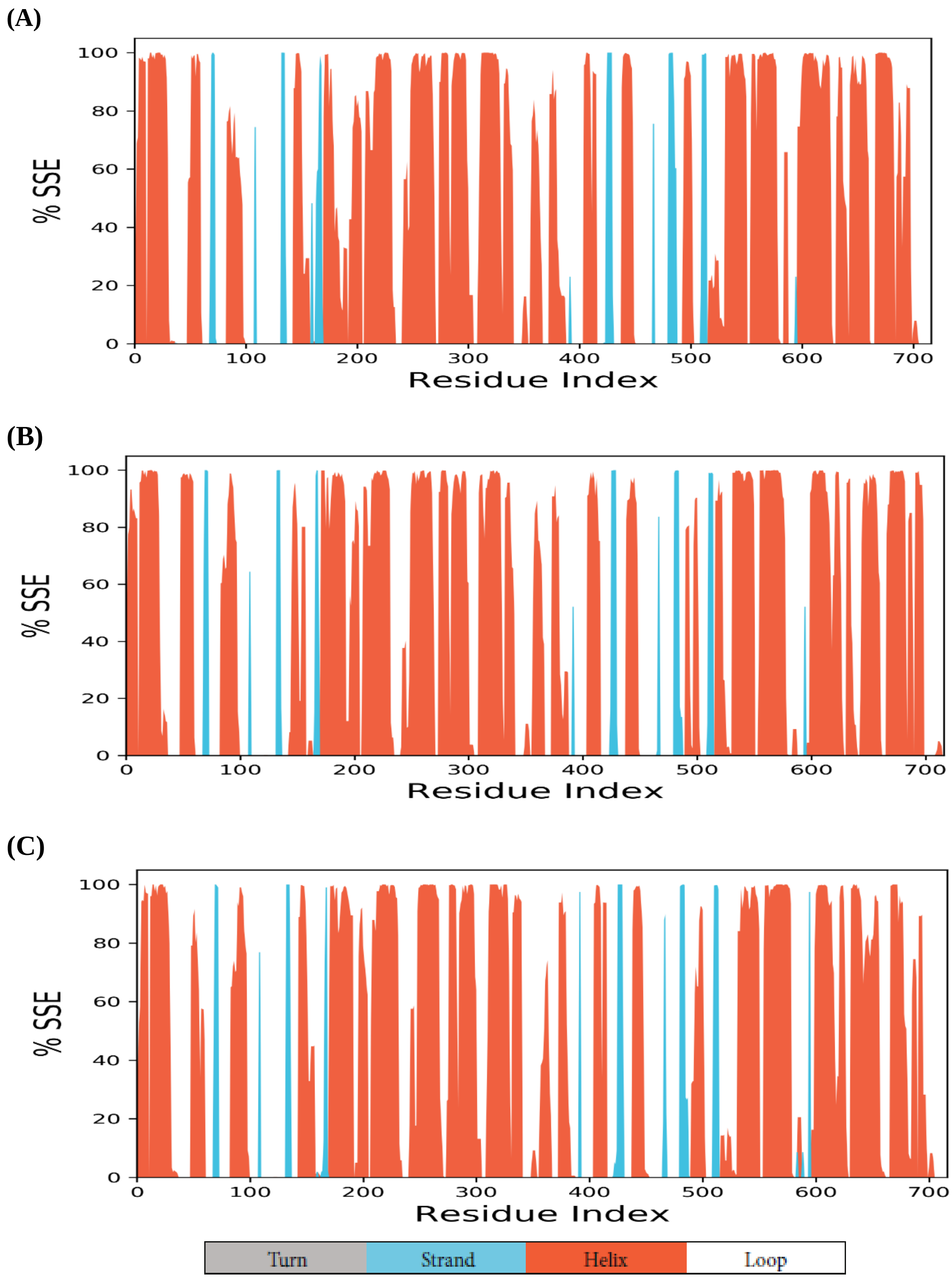

Figure S18. Protein secondary structure analysis of the individual apo-form MmpL3 trajectories one **(A),** two **(B)** and three **(C)** displaying the abundance of protein secondary structure elements.

(A)

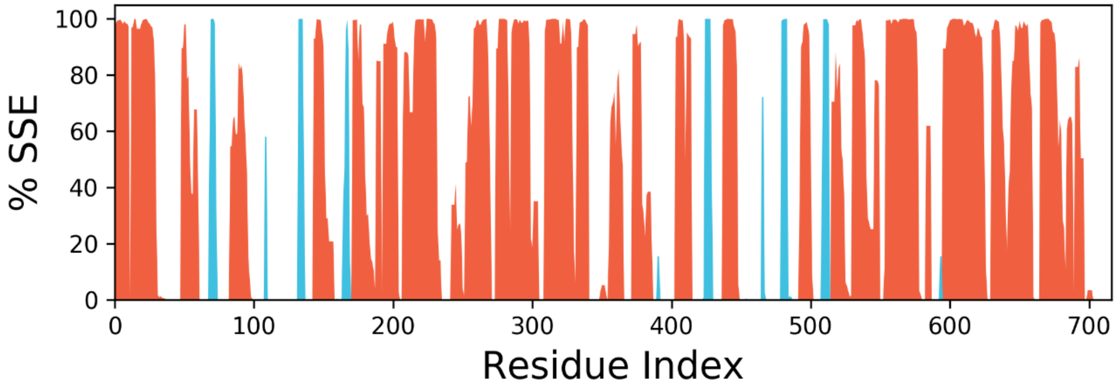

(B)

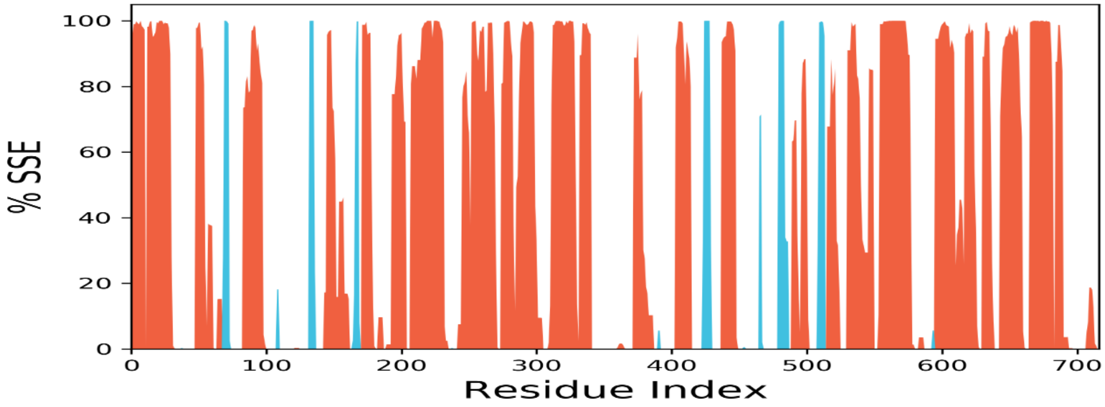

(C)

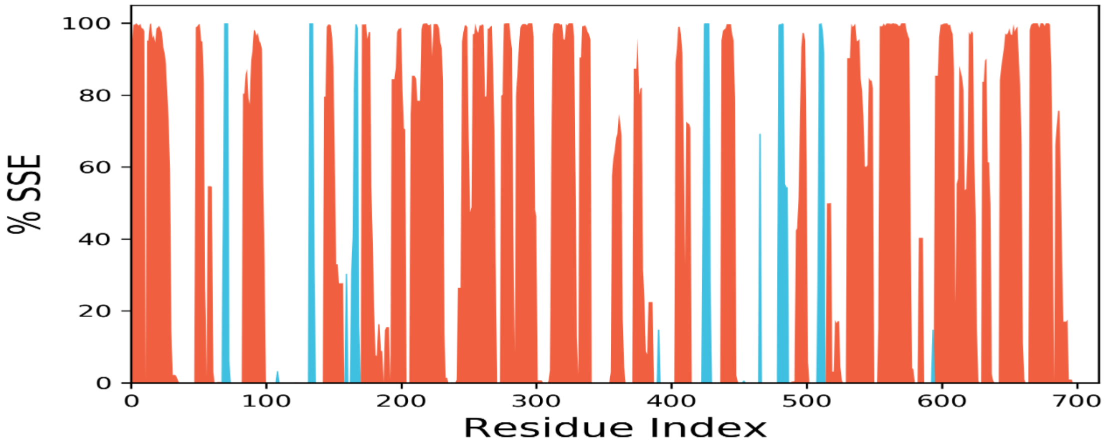

Figure S19. Protein secondary structure analysis of the individual holo-form MmpL3-SQ109 trajectories one **(A),** two **(B)** and three **(C)** displaying the abundance of protein secondary structure elements.

(A)

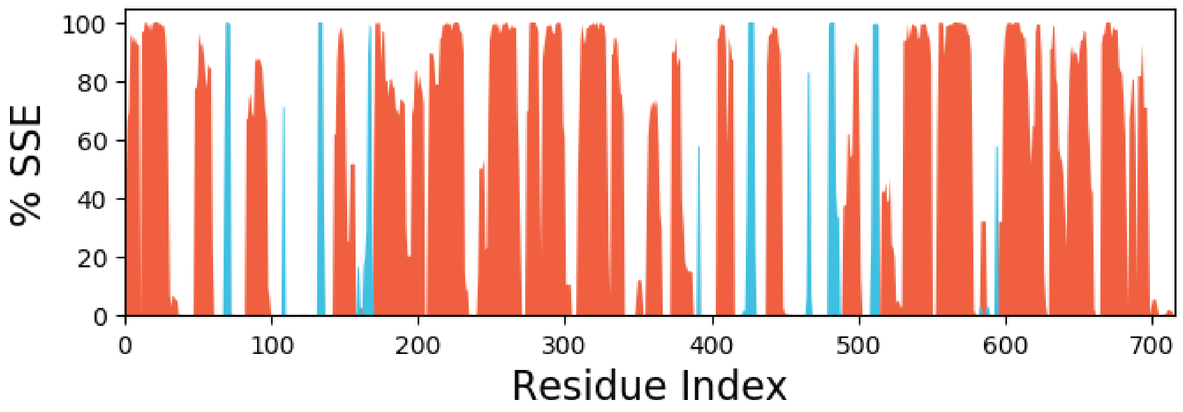

(B)

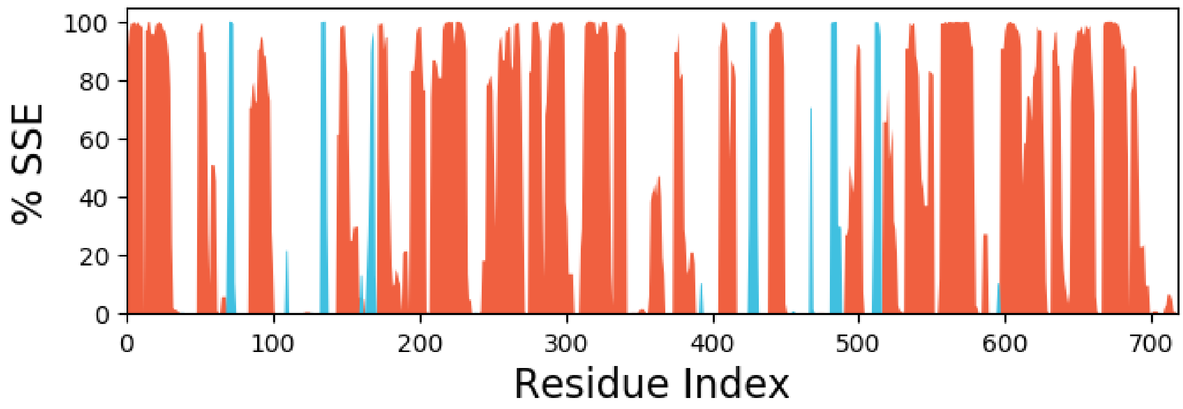

Figure S20. Secondary structure comparison of the combined trajectories of MmpL3 apo-form **(A)** and MmpL3-SQ109 holo-form **(B**) displaying the abundance of protein secondary structure elements.

A1	A2	A3	A4	A5
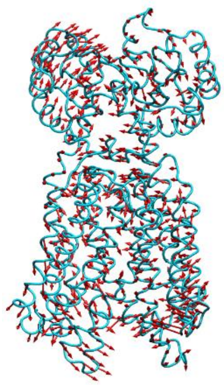	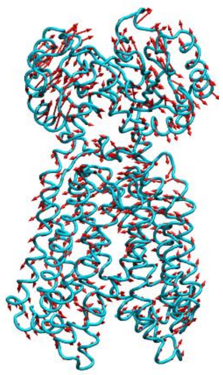	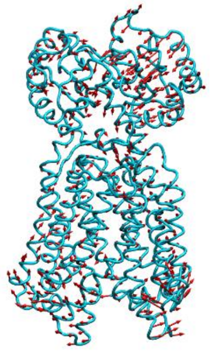	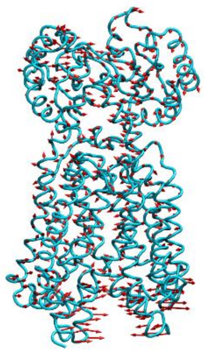	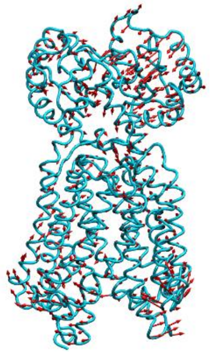
A6	A7	A8	A9	A10
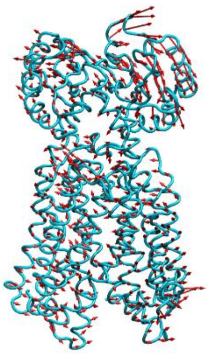	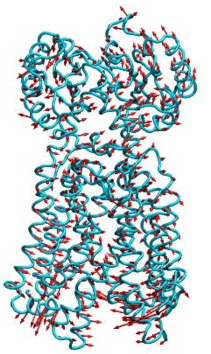	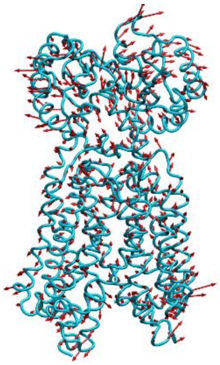	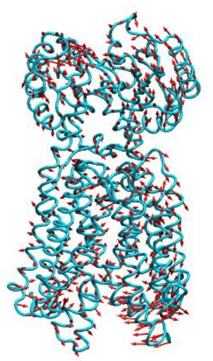	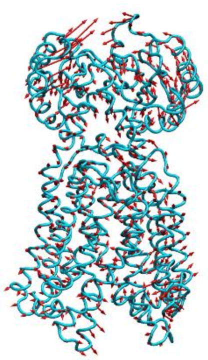

B1	B2	B3	B4	B5
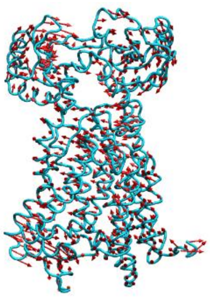	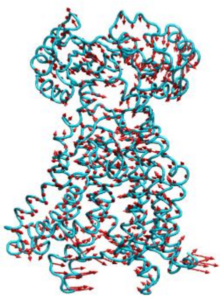	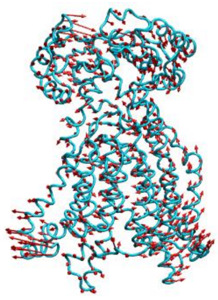	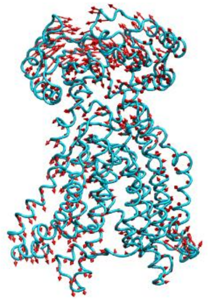	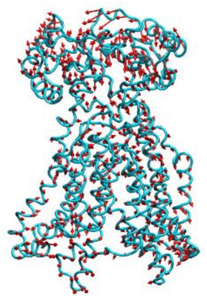
B6	B7	B8	B9	B10
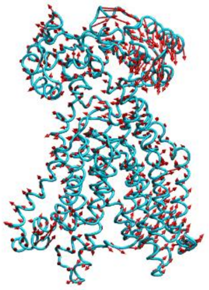	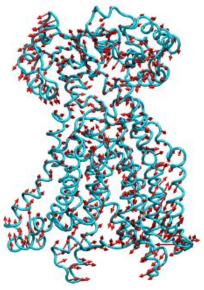	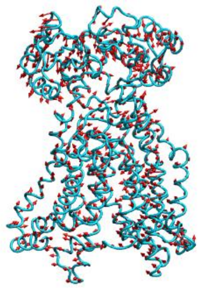	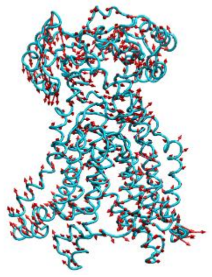	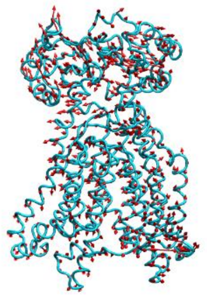

Figure S21.The top ten Normal Modes of the Apo trajectory (**A**) and Holo trajectory (**B**). Trajectory topology shown in cyan and normal mode movement depicted as red arrows. 10 normal mode analysis are depicted 1-10.

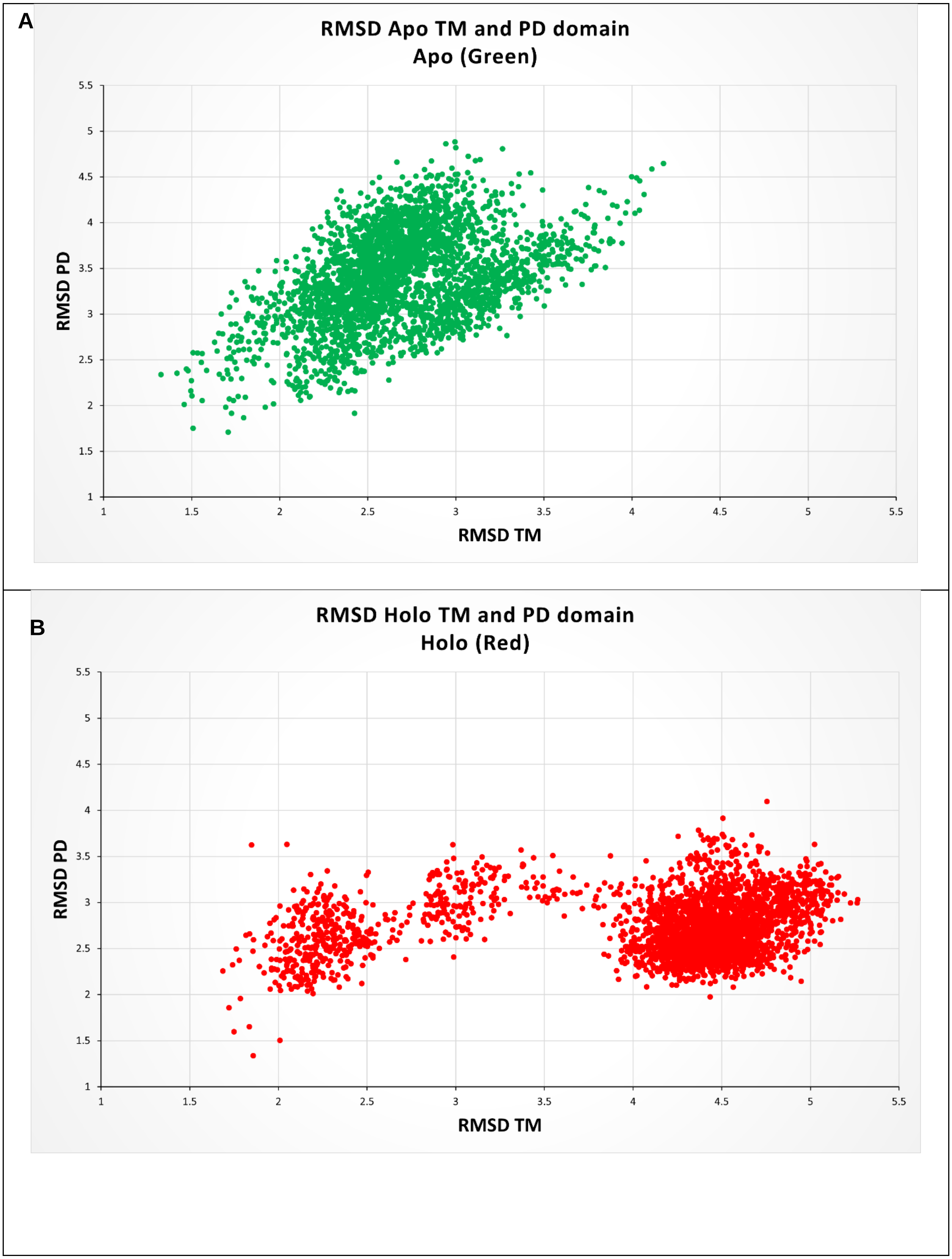
A
RMSD Apo TM and PD domain
Apo (Green)
RMSD PD
5.5
5
4.5
4
3.5
3
2.5
2
1.5
1
1.5
2
2.5
3
3.5
4
4.5
5
5.5
RMSD TM
B
RMSD Holo TM and PD domain
Holo (Red)
RMSD PD
RMSD TM

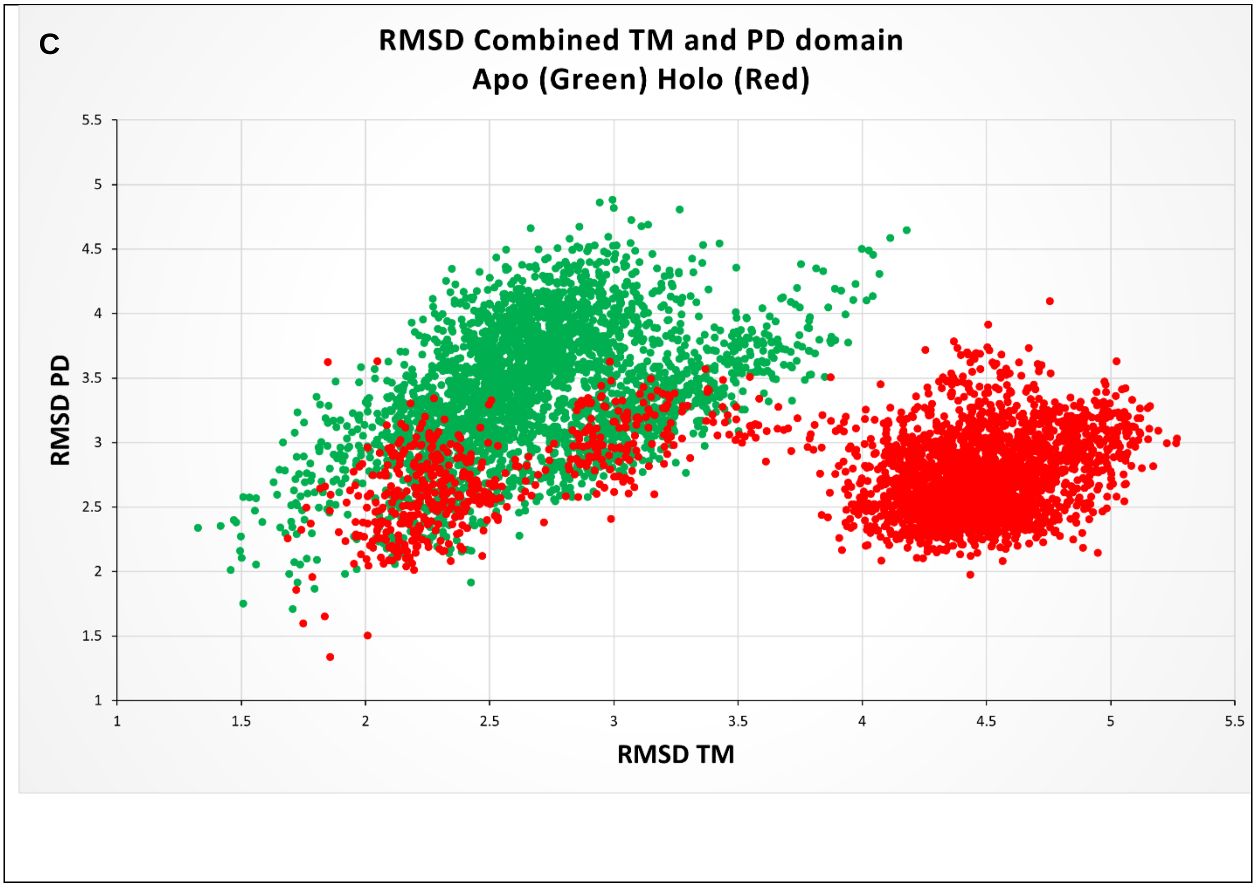

Figure S22. RMSD of trajectory snapshots of Apo (A)(Green), Holo(B)(Red), and combined (C). RMSD of Porter Domain (PD) was calculated through Desmond RMSD trajectory tool in Maestro. RMSD was calculated from the first frame of the apo(A) and holo(B) trajectories respectively. PD domain residues 35-169 and 421-547 were used to calculate PD RMSD. Transmembrane (TM) domain was calculated using residues 1-34, 170-420, and 548-730. Then RMSD for each frame of each structure was imported into excel where the RMSD for each frame was used in an XY scatter plot. PD domain RMSD was considered X axis and TM domain RMSD was considered Y axis.

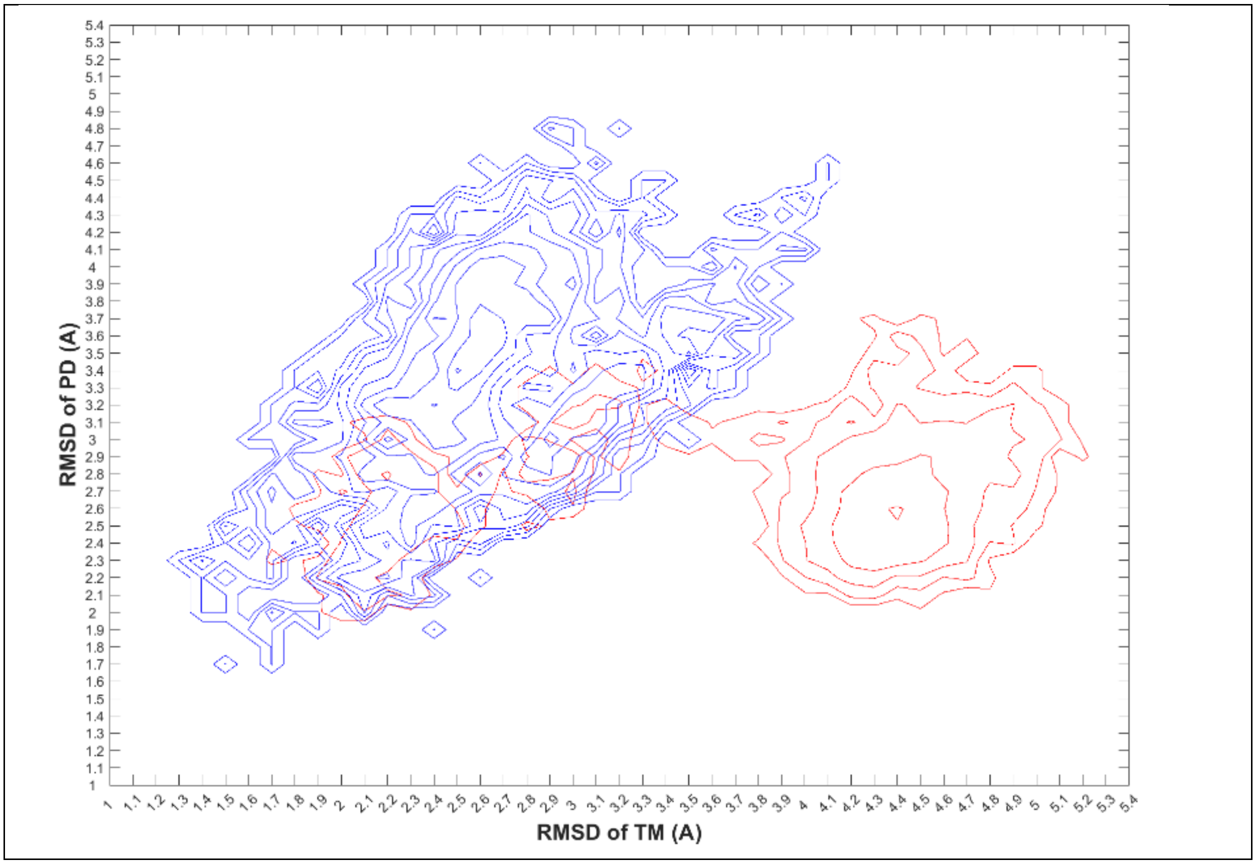

Figure S23 Combined Free Energy Landscape. Apo system is represented in blue lines and the holo system is represented in red lines.

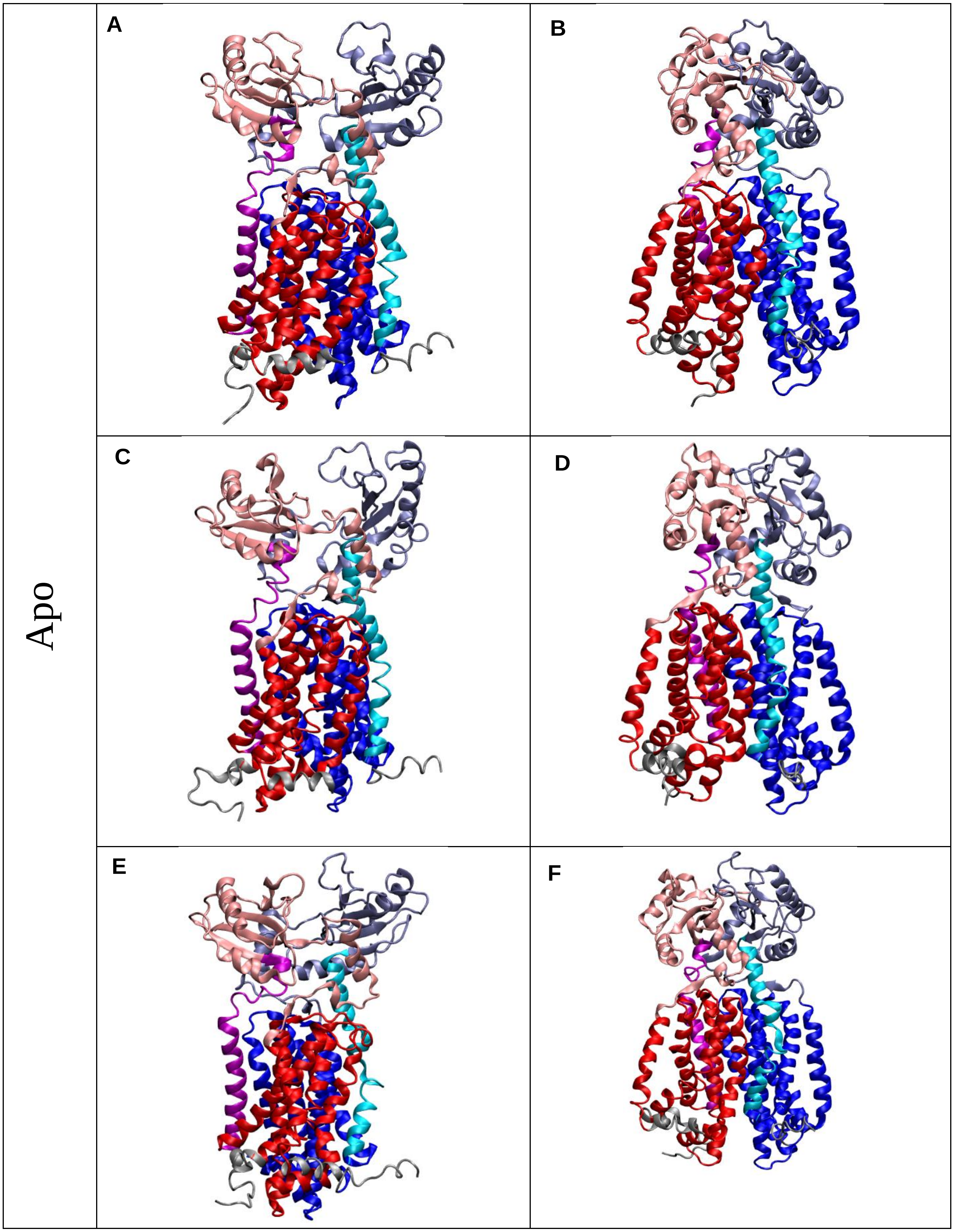
Apo
A
B
C
D
E
F

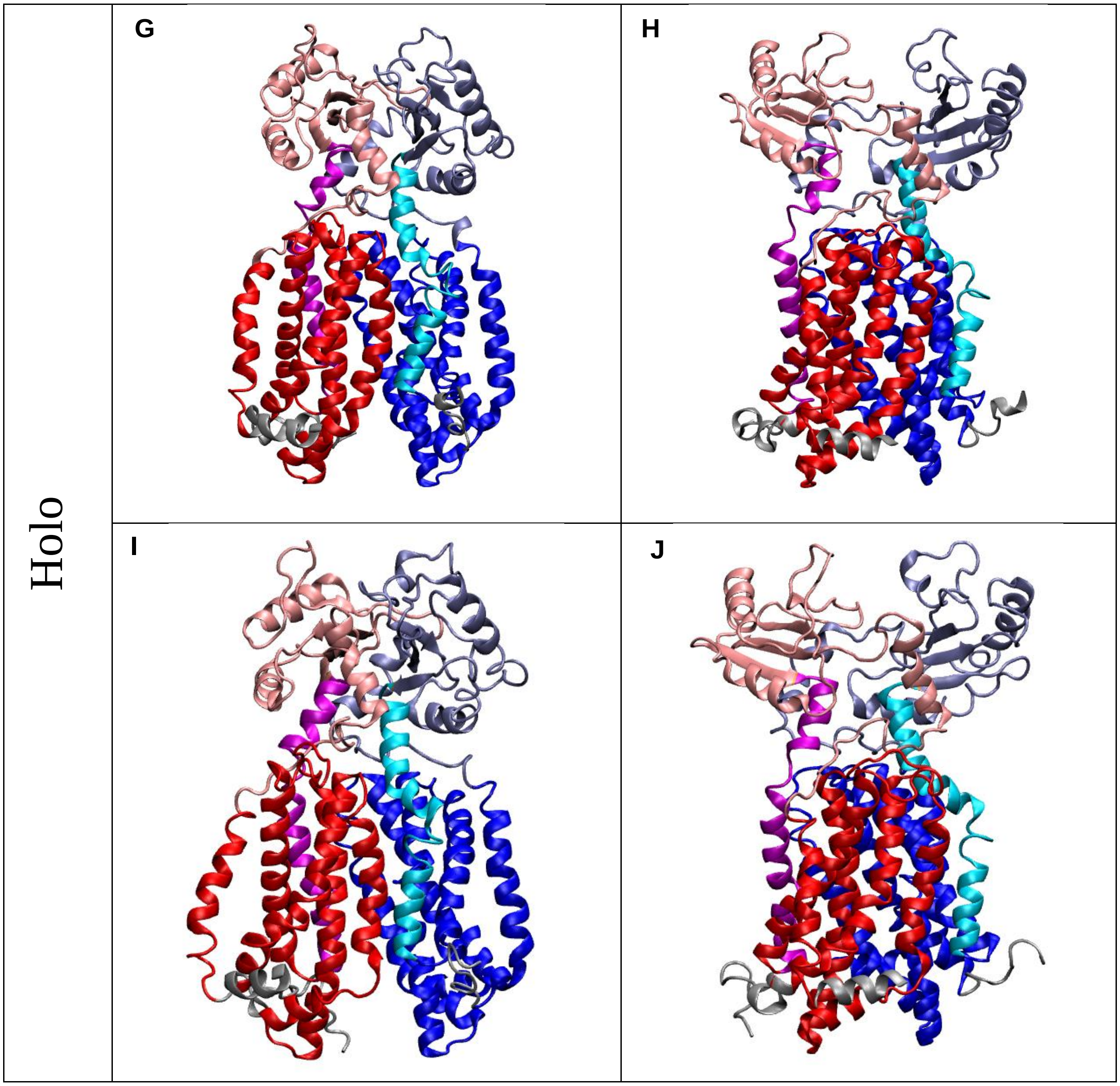
Holo
G
H
I
J

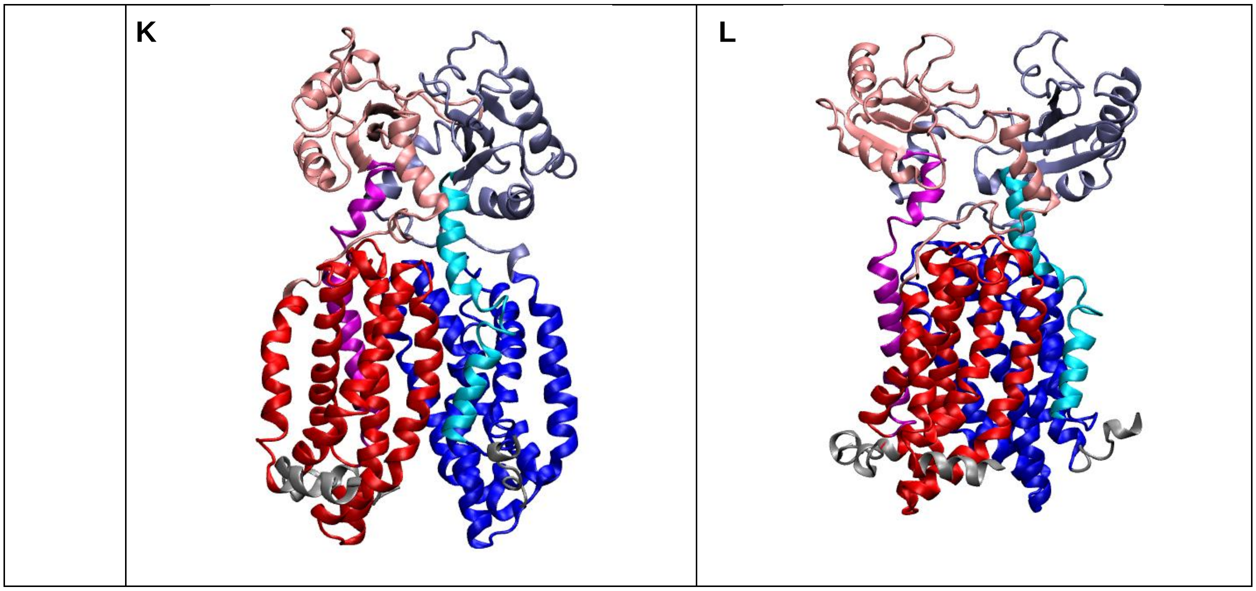

Figure S24 Free Energy Landscape Structures determined through RMSD of domains at lowest free energy valley. State 1 (2337) A,B TMD Closed PD open. State 2 TMD Open PD closed (2636) C,D. State 3 TM closed PD open (1872) E,F. Holo State 1 TM closed PD closed (295) G,H. Holo state 2 TM open PD closed (414) I,J and state 4 TM open PD closed (648) (K,L). Alternating structural view for key differences in TMD (left) and PD (right). Key structural elements highlighted as TMD1 (blue), TM2 (cyan), TMD2 (red) TM8 (magenta), PD1 (iceblue), PD2 (pink), a helices and C terminal connection cutoff (silver).

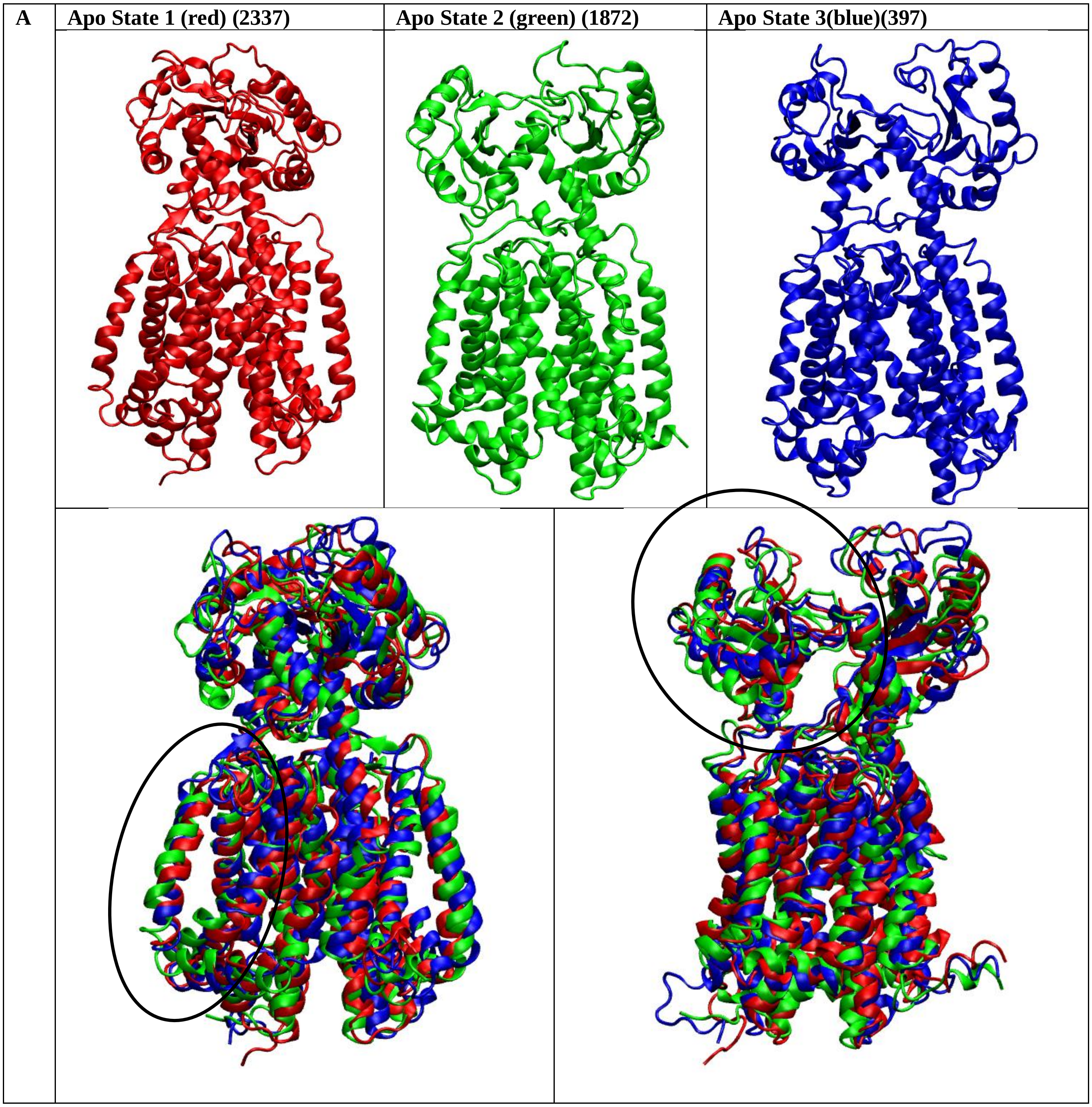
A
Apo State 1 (red) (2337)
Apo State 2 (green) (1872)
Apo State 3(blue)(397)

**B**	**Holo State 1 (magenta) Ligand:SQ109 (Orange)**	
	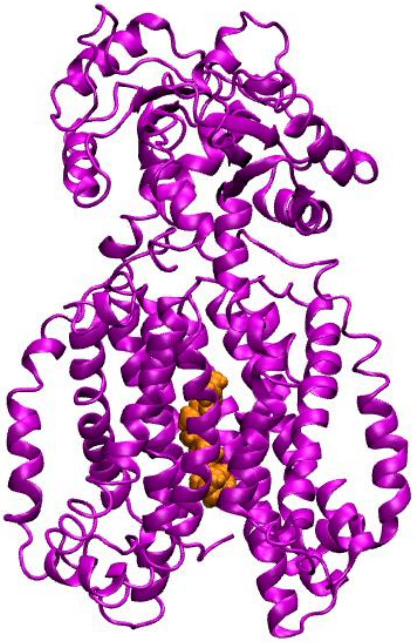	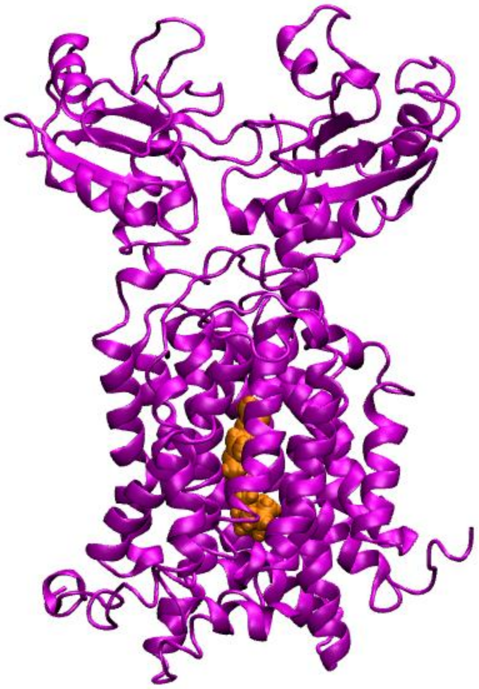
	**Holo State 2 (Orange) Ligand:SQ109 (green)**	
	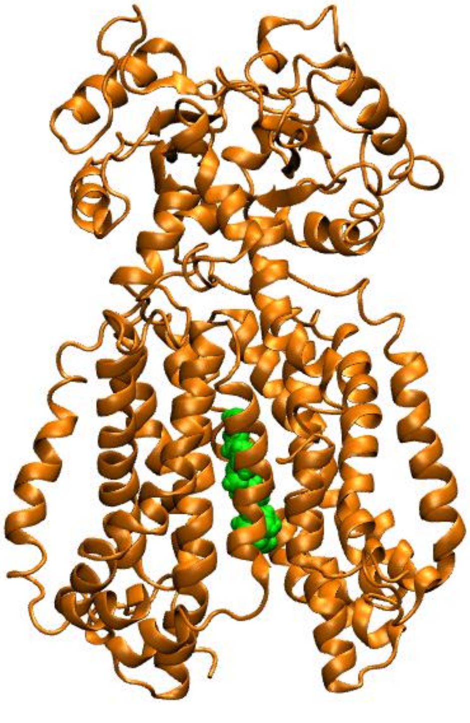	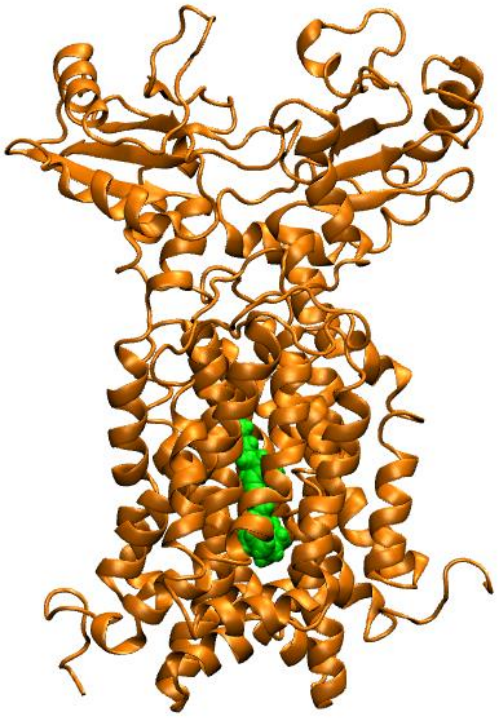
	**Holo State 4 (Cyan) Ligand:SQ109 (Orange)**	

	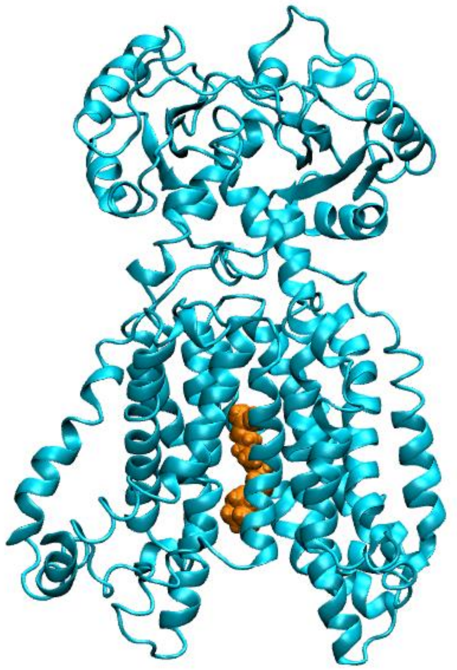	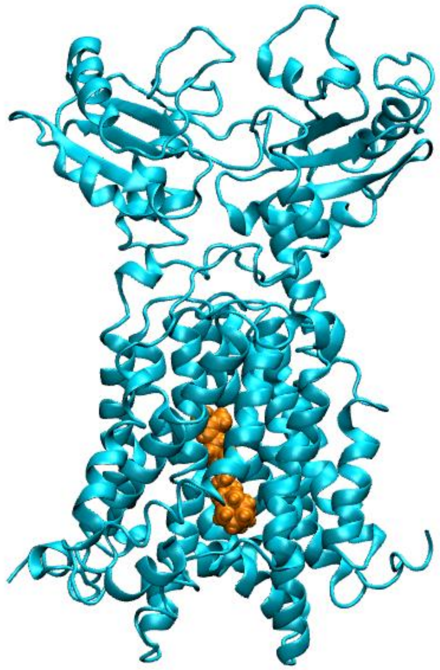
	**3 way comparison Holo states 1(Magenta), 2 (Orange), and 4 (Cyan)**	
	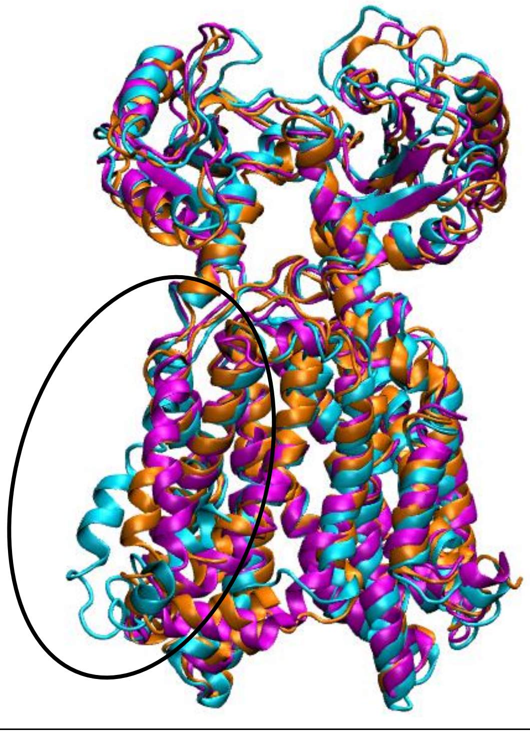	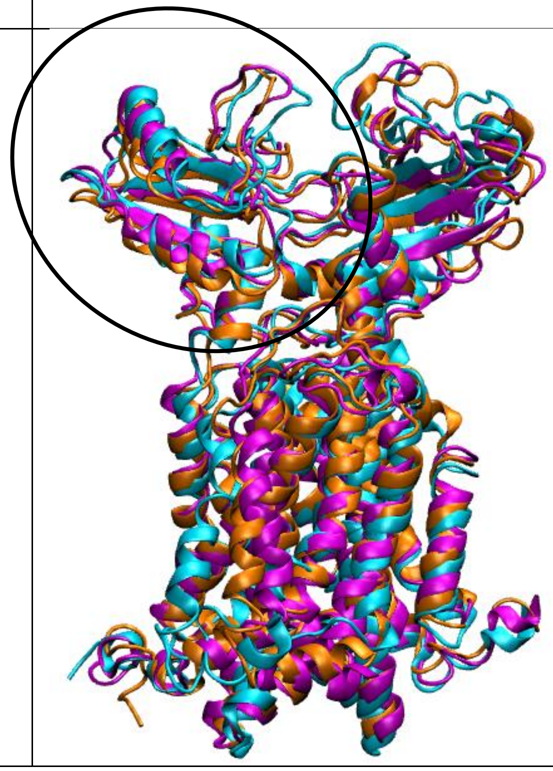

C

Apo State 1 (red) vs Apo State 2 (green)

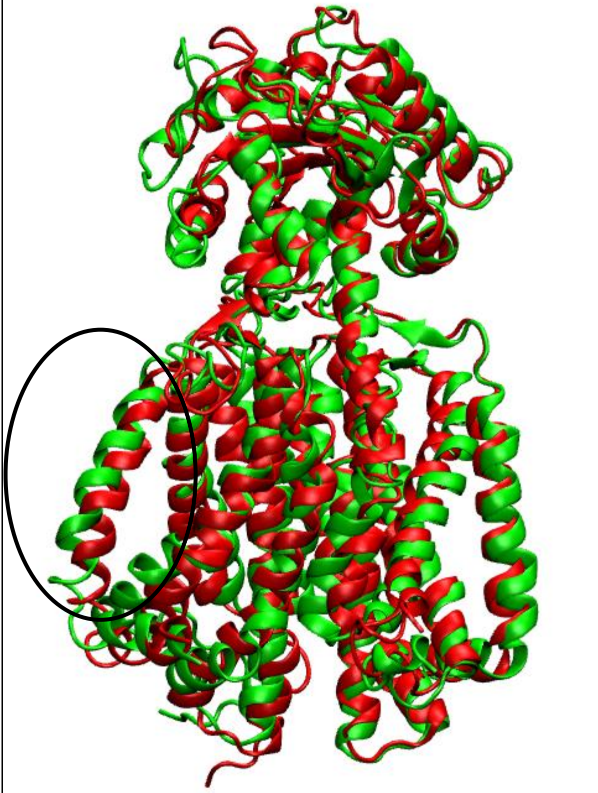

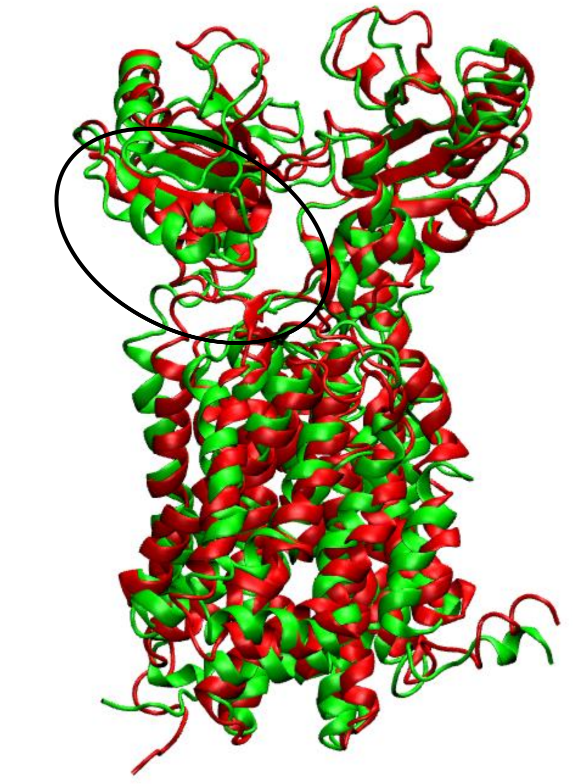

Apo State 1(red) vs Apo State 3(blue)

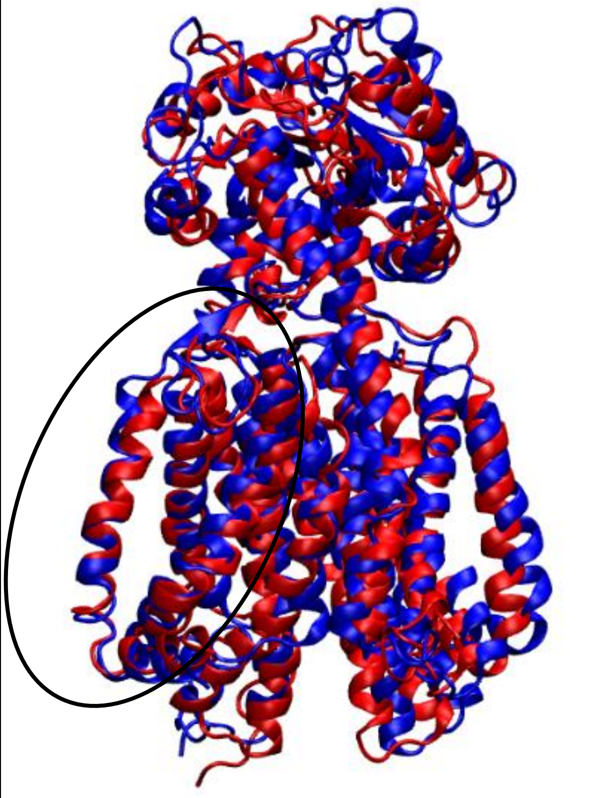

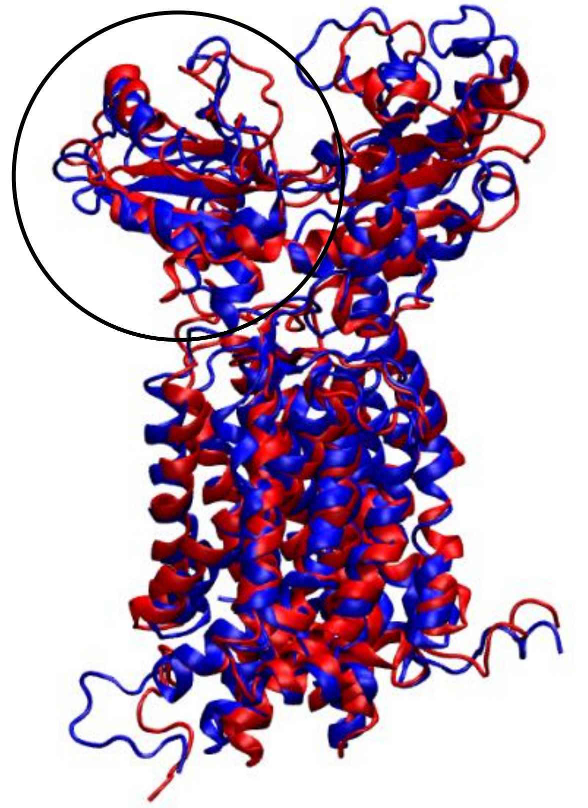

	Apo State 2(green) vs Apo State 3(blue)	
	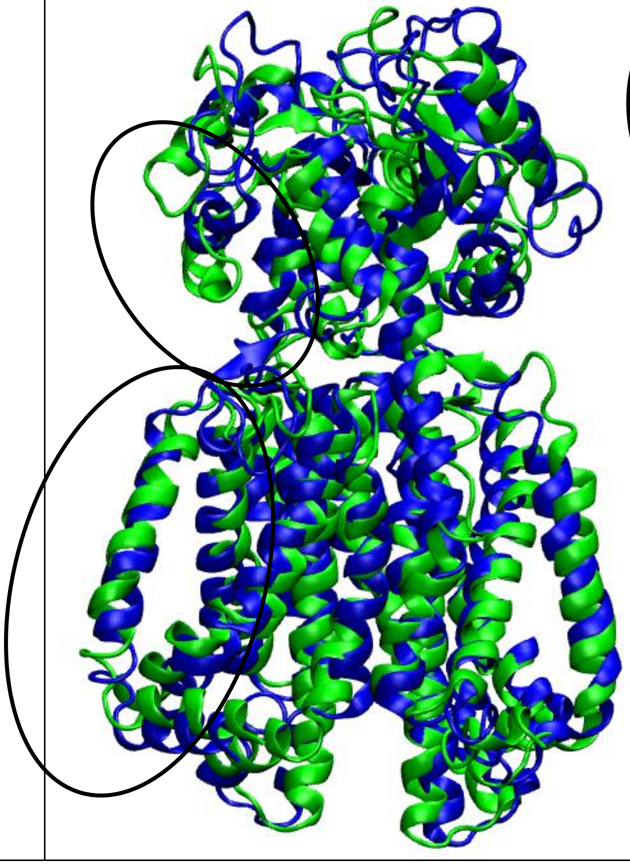	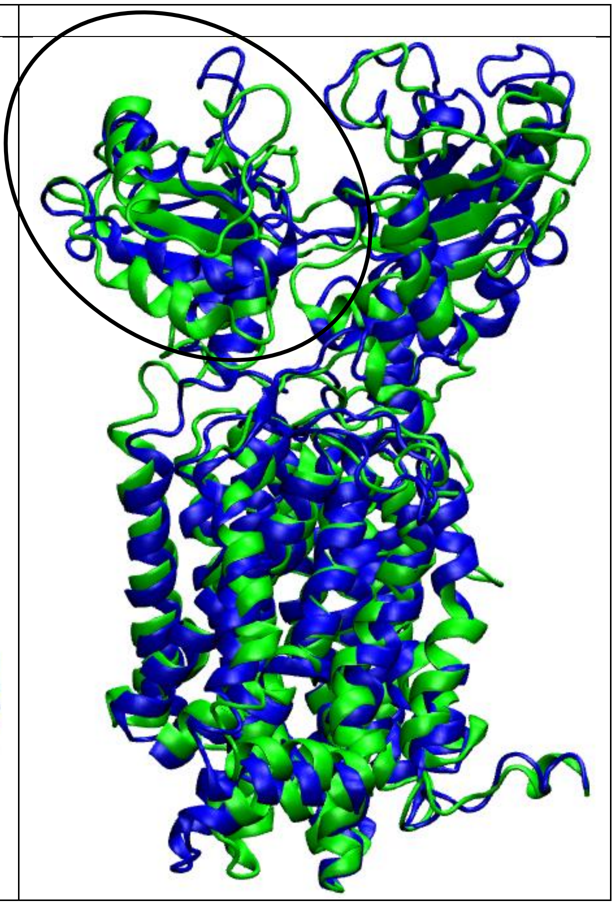

D	Apo State 1 (red) vs Holo state 1 (magenta)	
	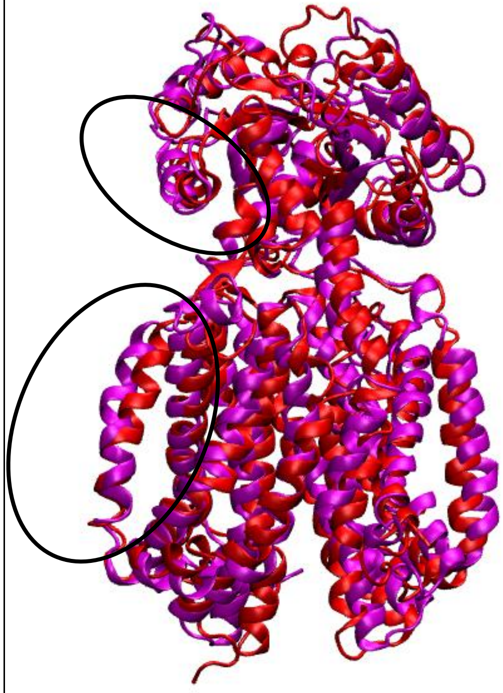	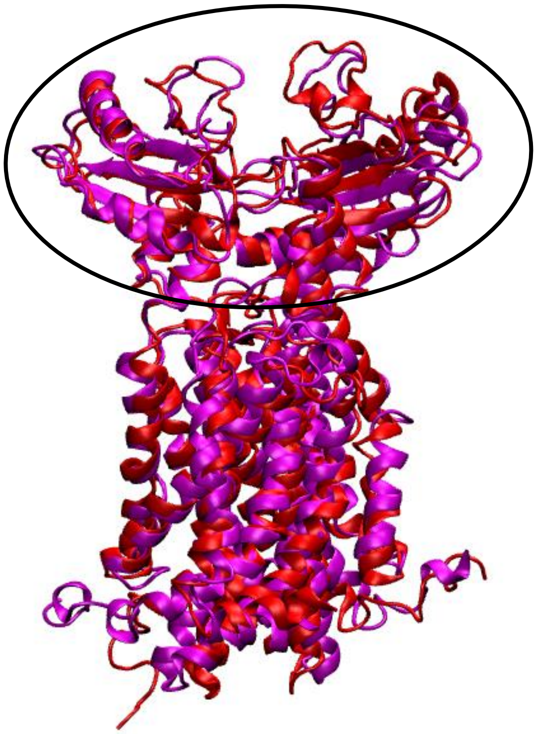
	**Apo State 2 vs Holo State 2**	
	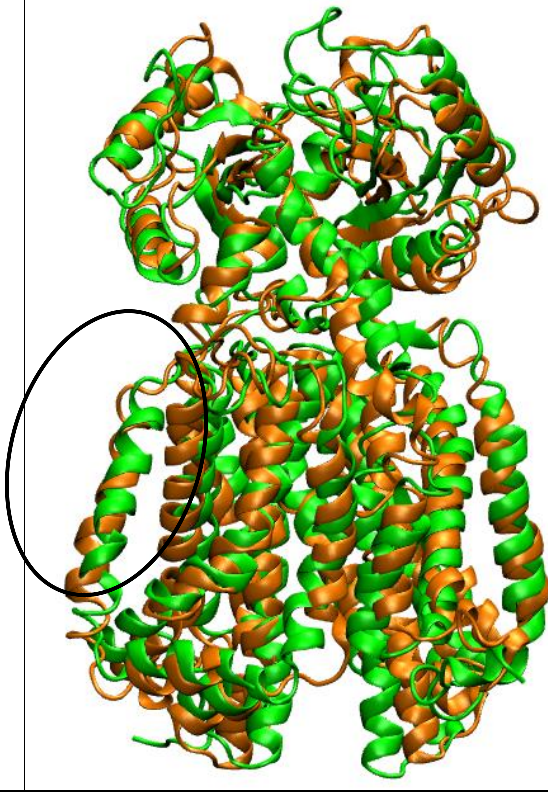	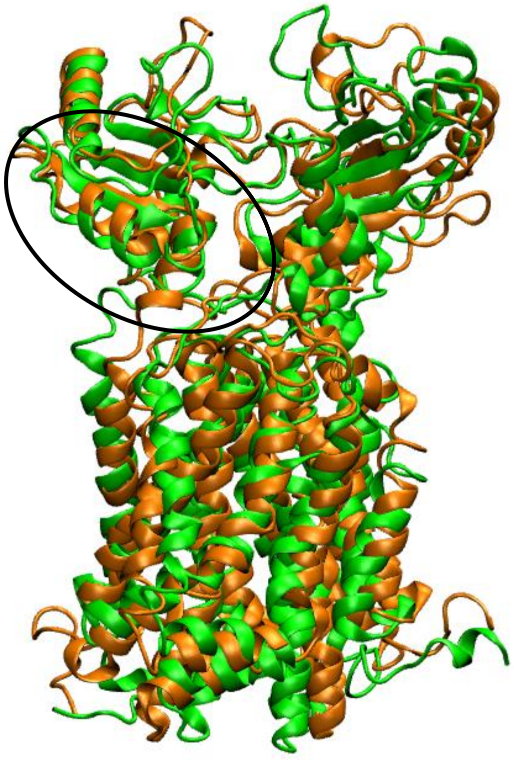

	**Apo State 2 (green) vs Holo State 4 (cyan)**	
	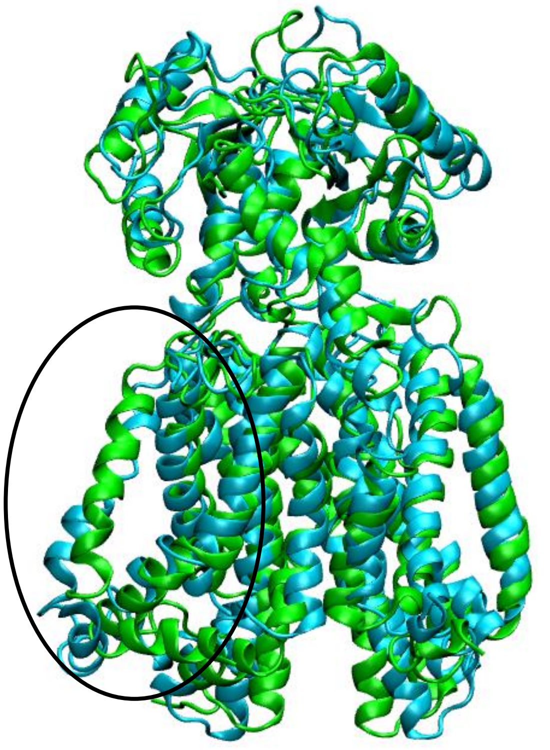	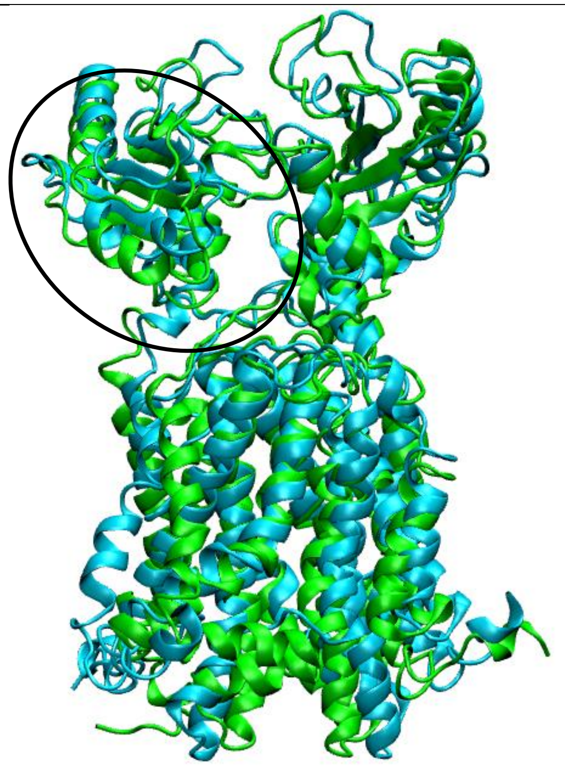
	**Apo State 3 (blue) vs Holo State 4 (cyan)**	
	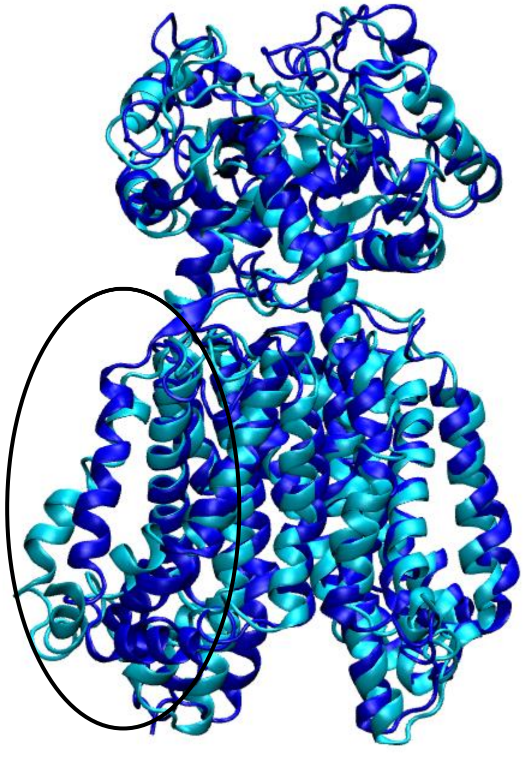	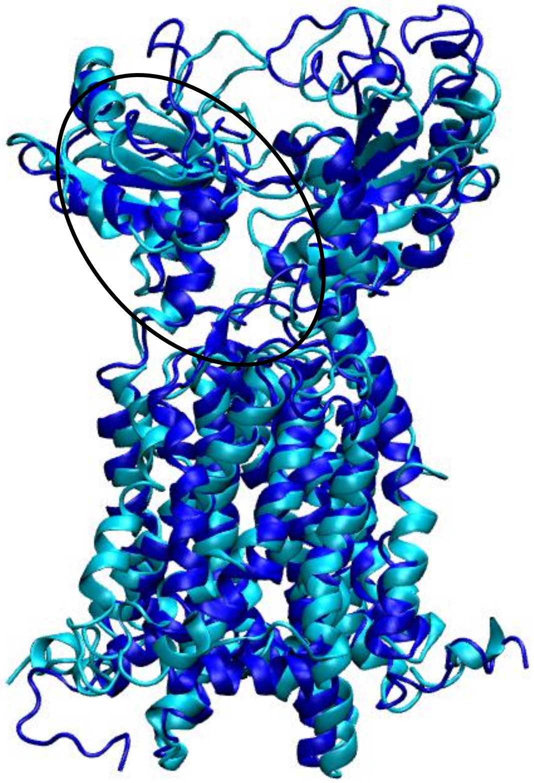

Figure S25 Free energy landscape Apo and Holo State Comparison. Apo States Determined through Free Energy Landscape. Circled Areas define key structural differences between open and closed TM and PD states define by TM7/TM8 movement and PD2 open and closing based on the TM helices movement. **(A)** State 1 Closed TM Closed PD(red), state 2 Open TM closed PD (green) and state 3 Closed tm and Open PD(blue). Holo States **(B)** State 1 Closed TM Closed PD (magenta) with SQ109 shown in VDW (orange), State 2 Open TM Closed PD (Orange) with SQ109 shown in VDW (Green), and Holo state 4 defines the new state with inhibitor bound showing very open TM domain and closed PD (Cyan) with Ligand SQ109 shown in VDW (orange). Apo State 3 way pairwise comparison **(C)** shows the different states between the apo system where TM domain will open and close allowing PD domain to open and close. State 1 and 2 show the opening of the TM domain and in state 3 the closing of the TM domain and opening of PD domain. In holo systems **(D)** both states TM domain remains in the open state causing PD domain to remain closed. Comparison of Apo vs Holo states **(E)** comparing Open TM Apo state 2 and Holo state 1 and Closed TM states Apo state 1&3 and Holo state 2

	Apo State 1	Holo State 1
PD domain	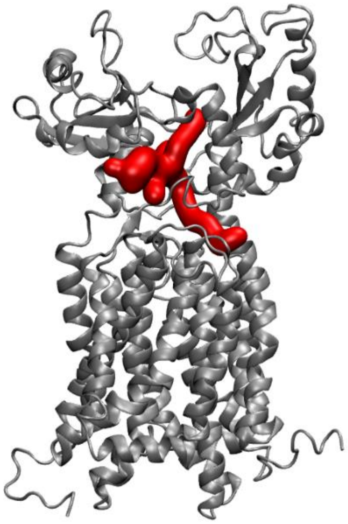	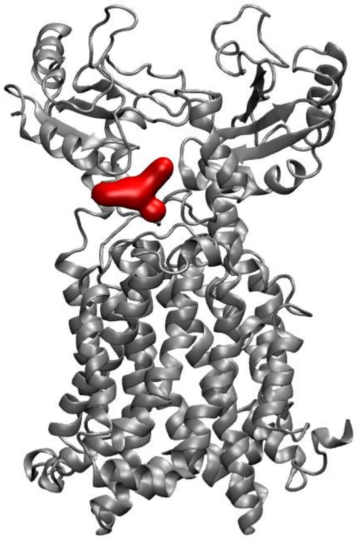
TM domain	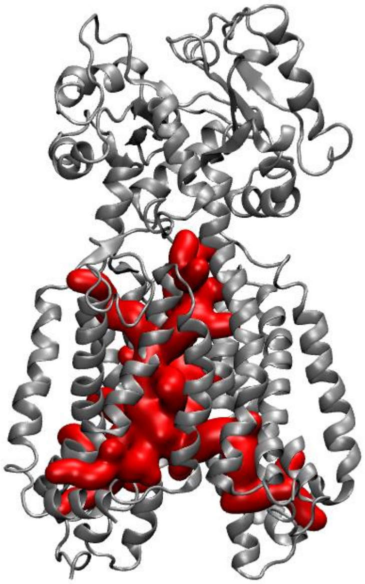	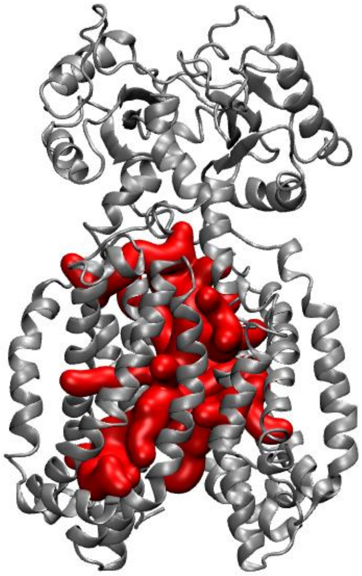

	Apo State 2	Holo State 2
PD domain	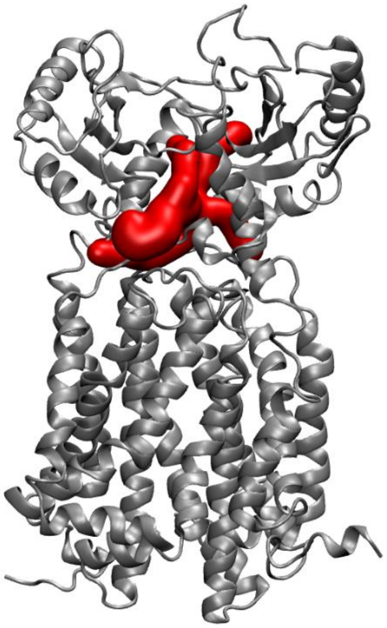	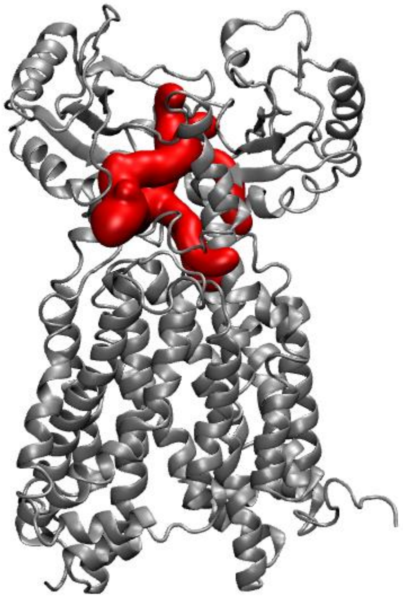
TM domain	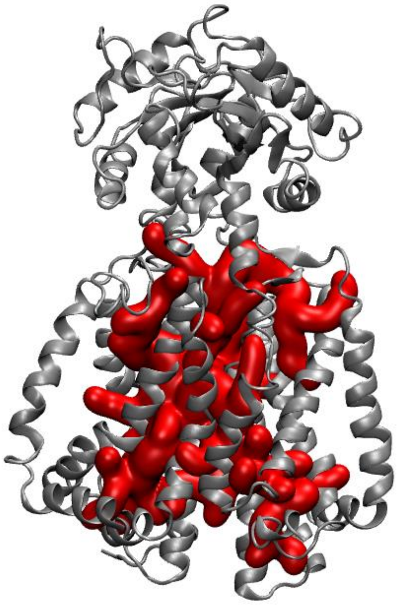	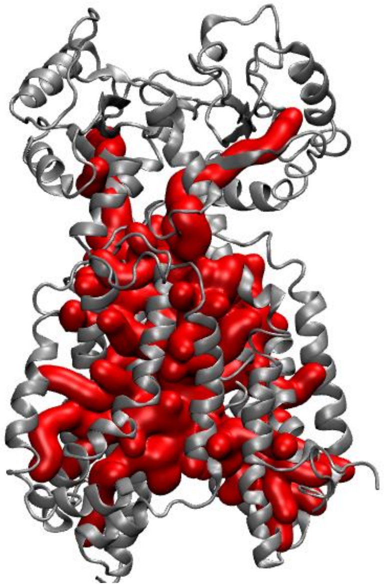

	Apo State 3	Holo State 4
PD domain	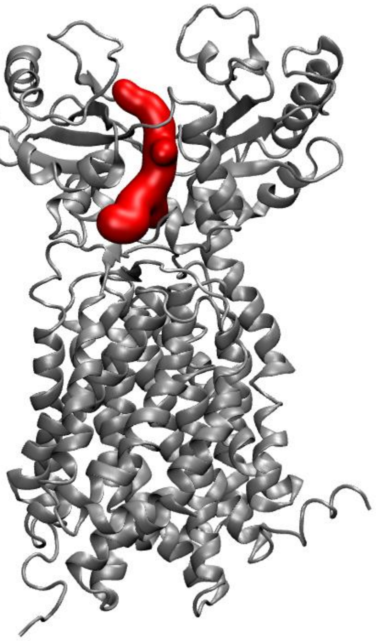	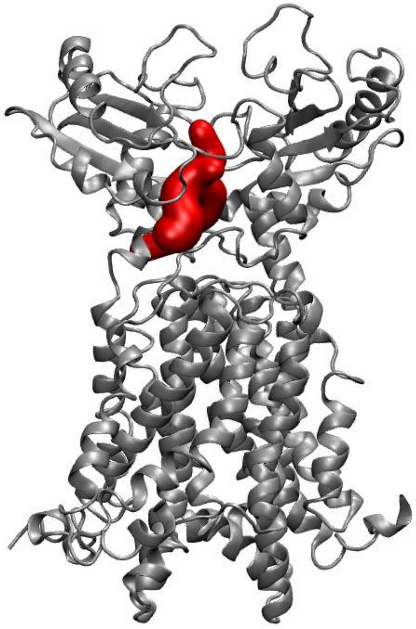
TM domain	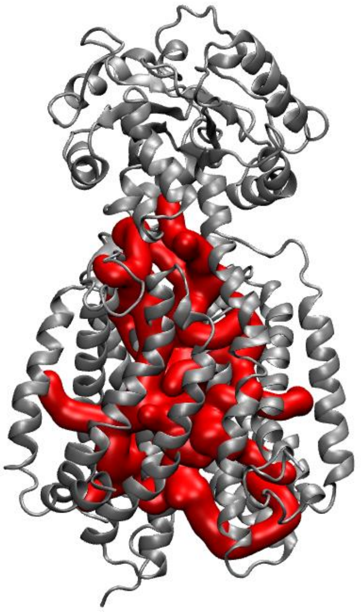	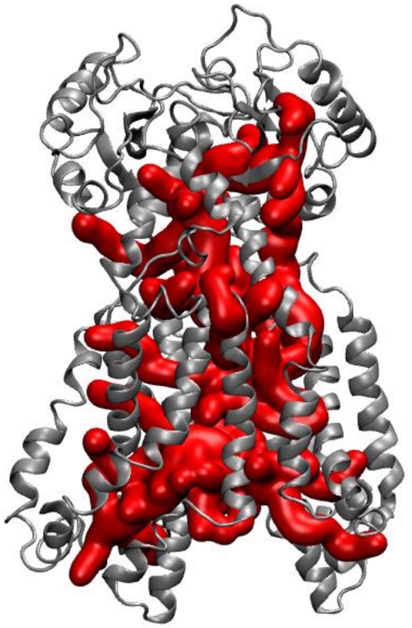

Figure 26 Caver analysis of all 4 states of Apo and Holo structures. Channels can be shown entering PD domain but not exiting the PD domain in any state. TM domain shows overall flooding of the channel. Pairwise comparisons of states shown for Apo and Holo systems 1:1, 2:2, and, 3:4.

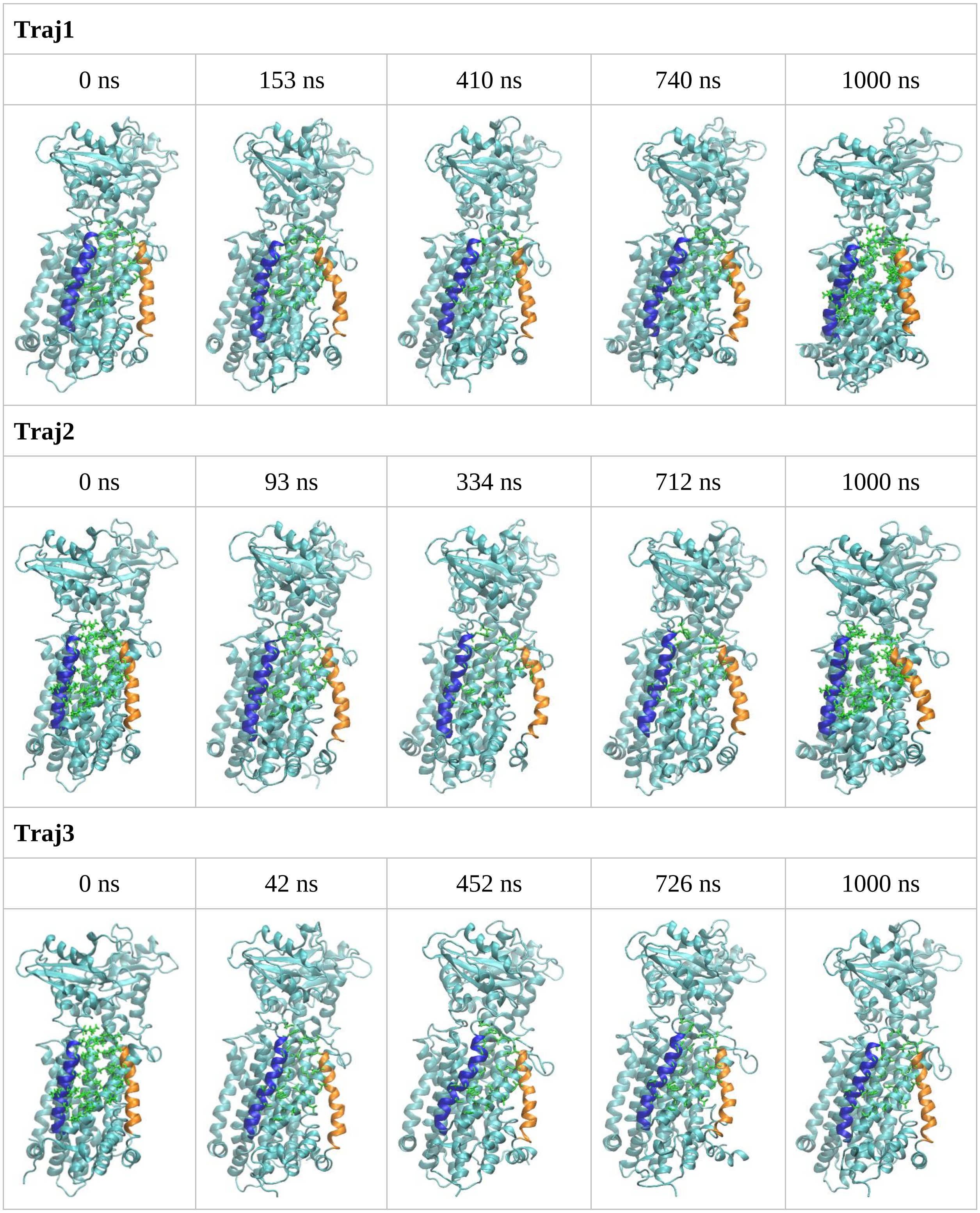

Figure S27. Dynamics of TM6 (orange, residues 405 to 422) and TM7 (blue, residues 561 to 583) of MmpL3 **a**po-form throughout each trajectory. Residues involved with initial TMM substrate binding are represented as green sticks.

**Traj1**				
0 ns	217 ns	455 ns	638 ns	1000 ns
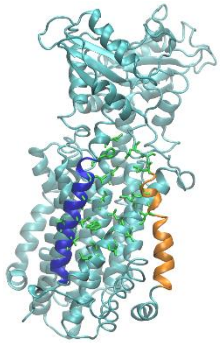	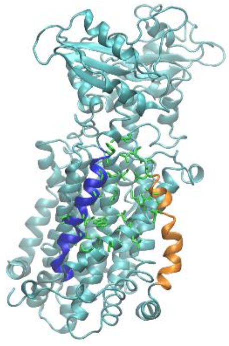	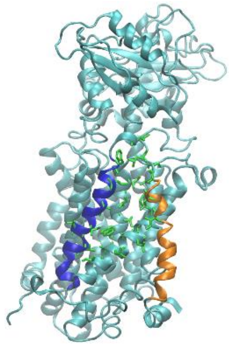	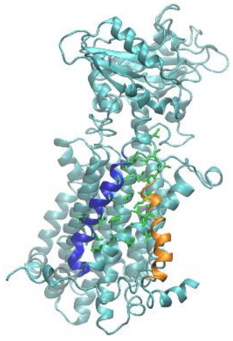	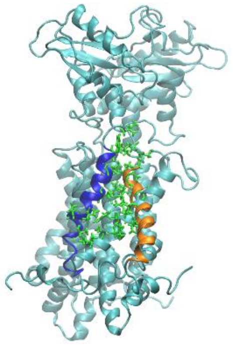
**Traj2**				
0 ns	274 ns	553 ns	887 ns	1000 ns
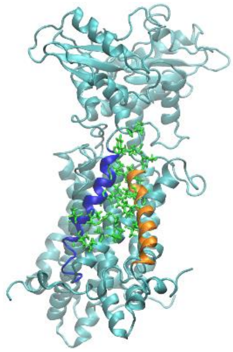	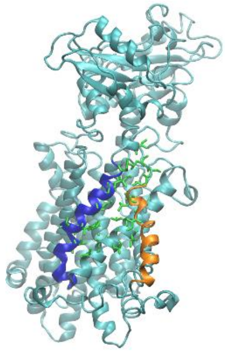	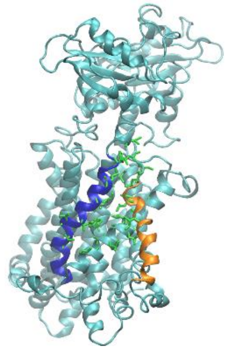	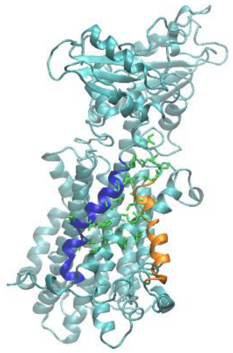	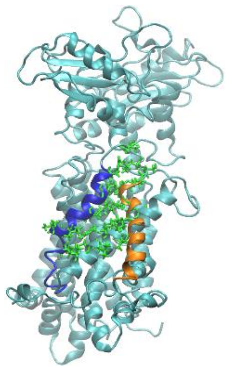
**Traj3**				
0 ns	244 ns	590 ns	856 ns	1000 ns
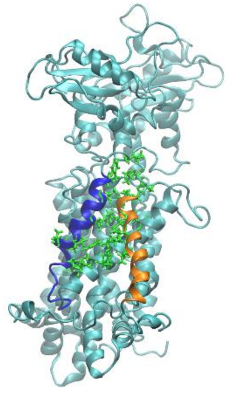	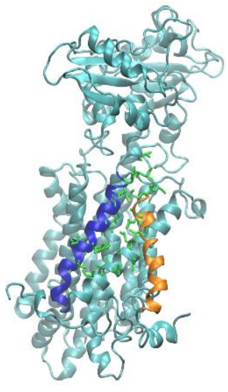	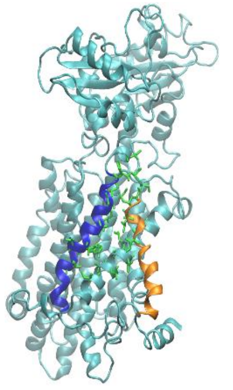	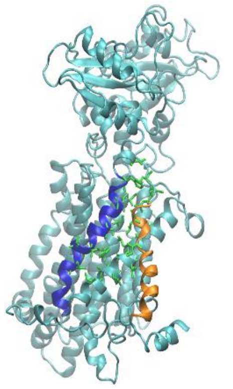	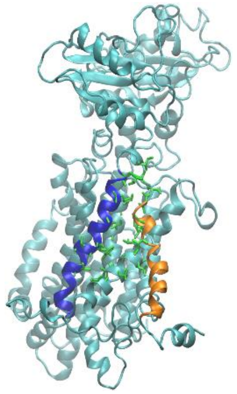

Figure S28. Dynamics of TM6 (orange, residues 405 to 422) and TM7 (blue, residues 561 to 583) of MmpL3 Holo-form throughout each trajectory. Residues involved with initial TMM substrate binding are represented as green sticks.

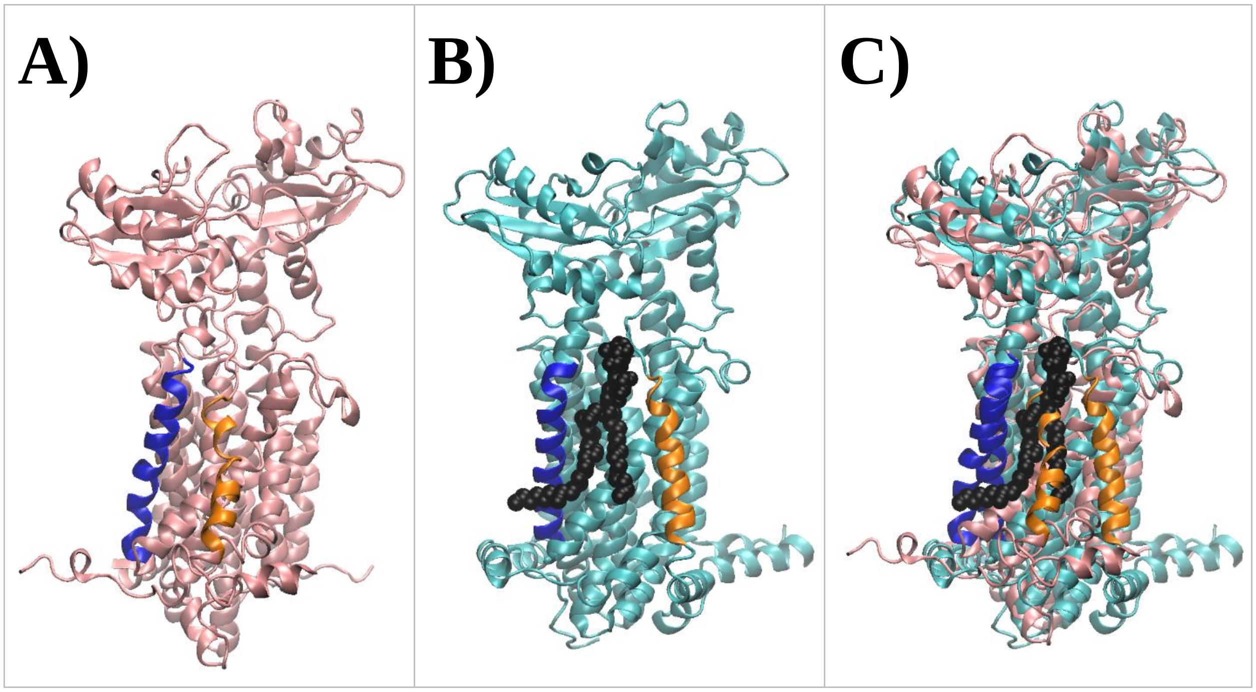

Figure S29. Conformations of TM7 (orange) and TM8 (blue) in MmpL3 holo-form structures. **A)** MD simulation structure (pink) containing SQ109 (not shown). **B)** Solved crystal structure (cyan) containing bound TMM (black) (PDB ID: 7N6B). **C)** Superimposition of the two holo structures.

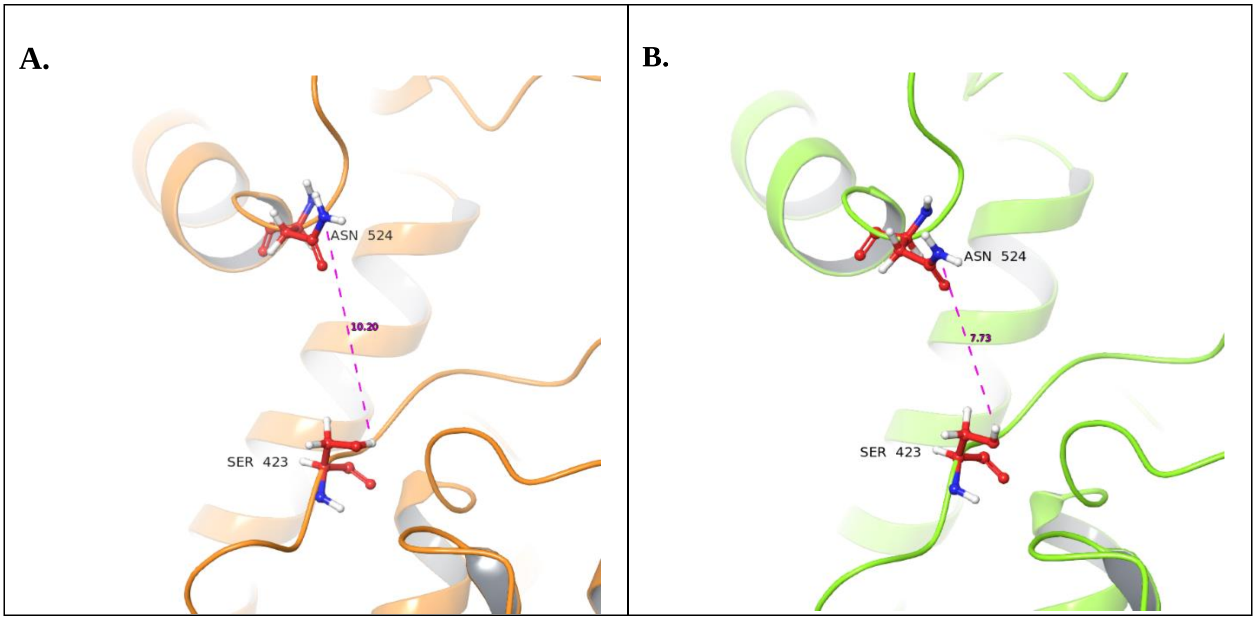

Figure S30. TMM Channel pore size formed by distance between gating residues S423 and Asn524 in the apo-form and holo-form structures. Channel is considered open at ~8 Å. This binding channel opens and closes to allow entry of TMM substrate to bind deeper into the porter domain.

APO
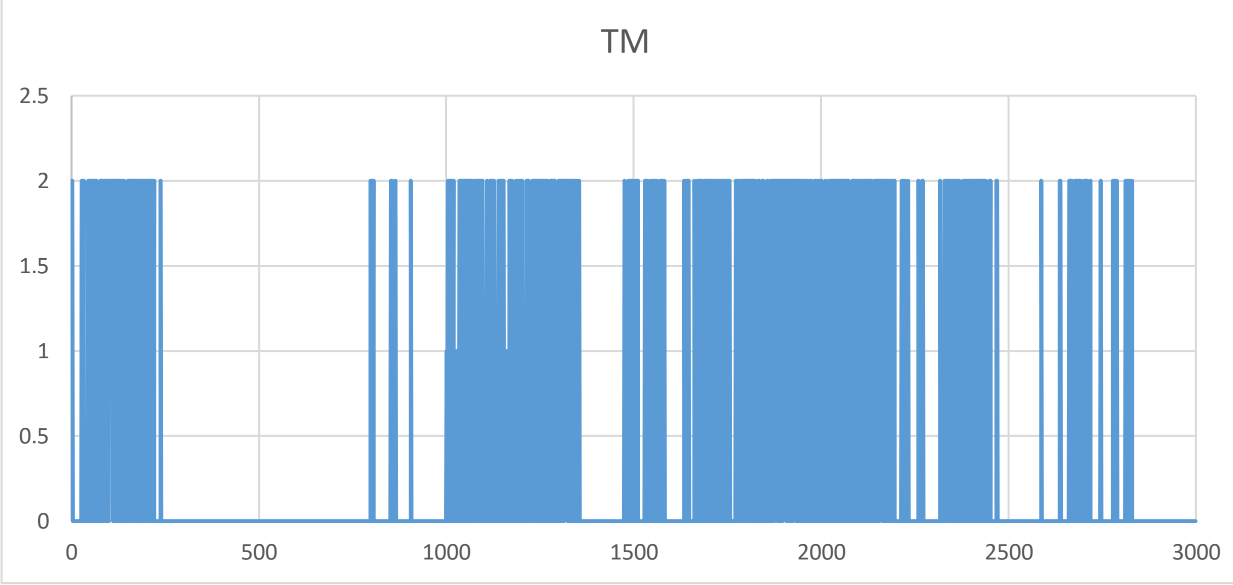
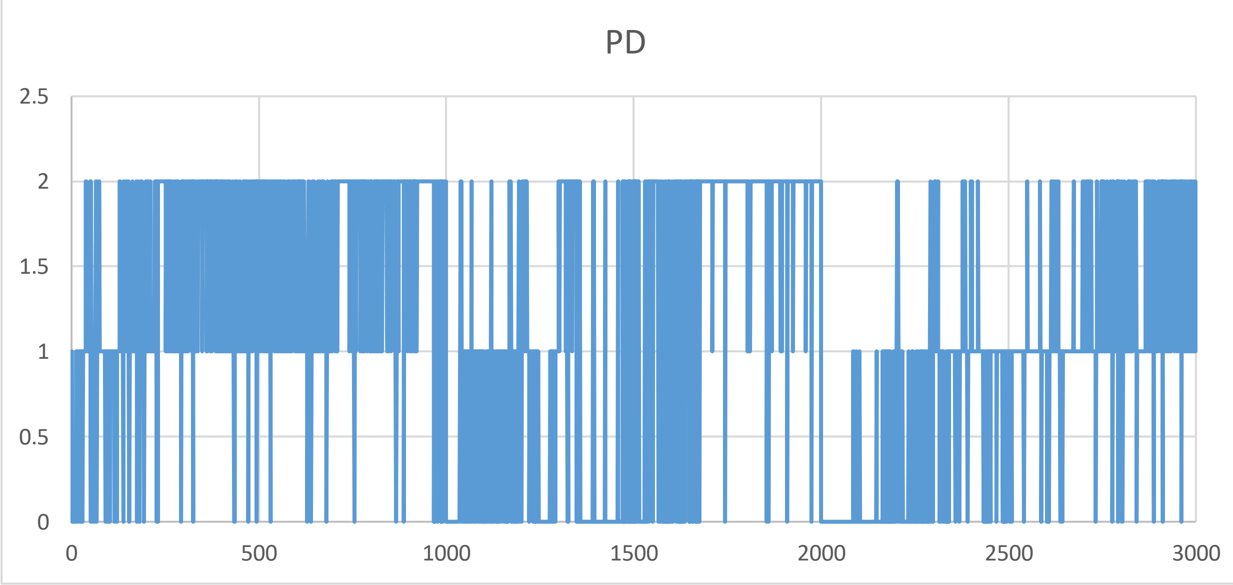

TM
2.5
2
1.5
1
0.5
0
0
500
1000
1500
2000
2500
3000

PD
2.5
2
1.5
1
0.5
0
0
500
1000
1500
2000
2500
3000

HOLO

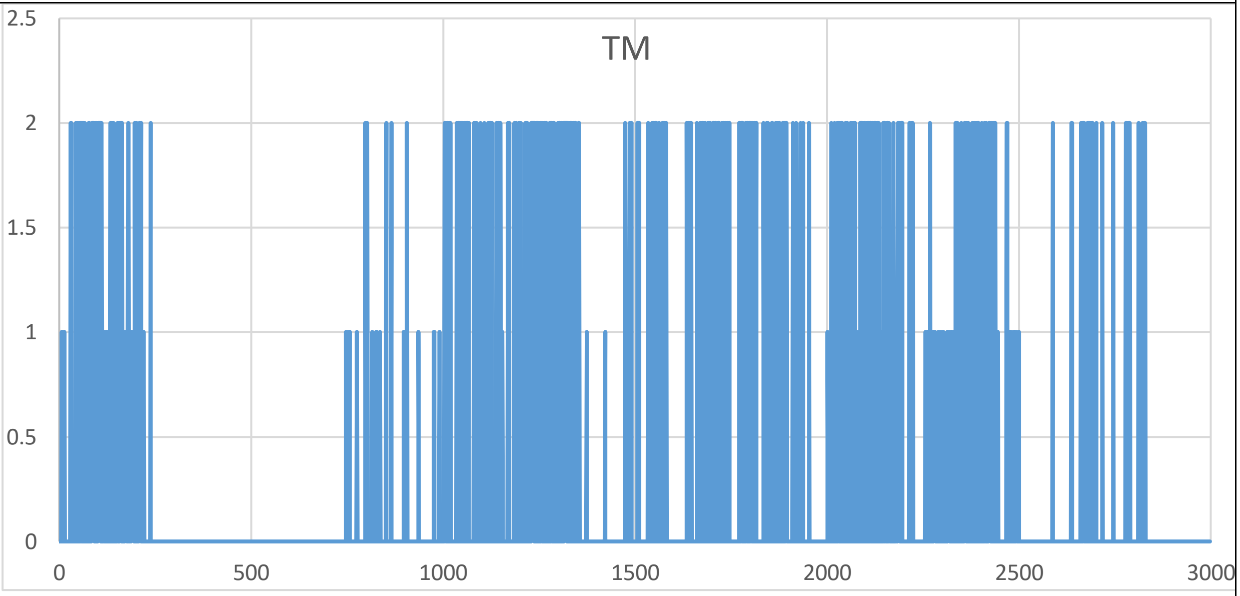

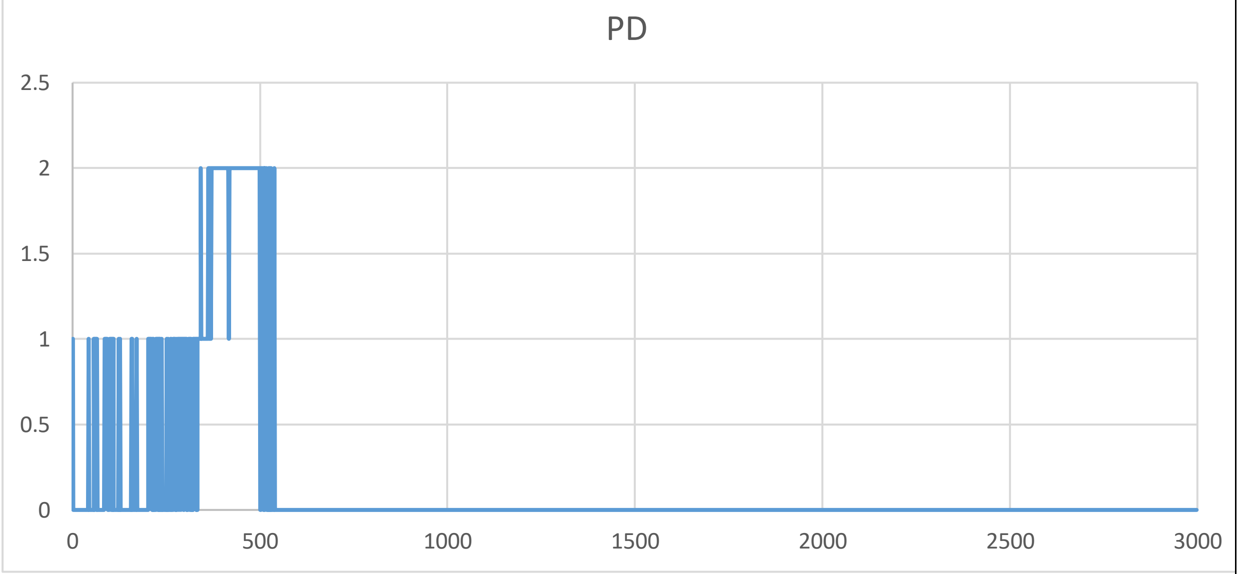

Apo States	TM state 1	TM state 2	TM state 3	PD state 1	PD state 2	PD state 3
State 1	55	0	0	1133	0	0
State 2	0	583	0	0	1138	0
State 3	0	0	25	0	0	156

Holo State	TM state 1	TM state 2	TM state 4	PD state 1	PD state 2	PD state 4
state 1	169	0	0	133	0	0
state 2	0	2158	0	0	152	0
state 4	0	0	383	0	0	363

Figure S31 Kinetic pathway analysis. Analysis of 4 states of MMPL3 in Apo and Holo System using RMSD data of Free Energy Landscape. Parameters for states were used in relation to our energy state structures. State 1: TMD:1.9-2.3A PD 1.5-2.7. State 2 TMD2.4-3.1A PD 2.71-3.3A. State 3: TMD 2.3-2.39 PD 3.4-4.5. State 4: TMD 3.11-5.55A PD 1.5-2.7AThe apo system goes through the range of 3 states with brief overlapping in states 1 and 3 for the TM domain and 1 and 2 for the PD domain which is expected as they share similar RMSD values. Holo system briefly shows possible state 4 in the first 500s of simulation for PD but then remains in a locked state 1 conformation for the rest of the simulation while the TM domain moves across the 3 states mainly converging in between state 2 and 3.

(A) Front	(B) Extracellular

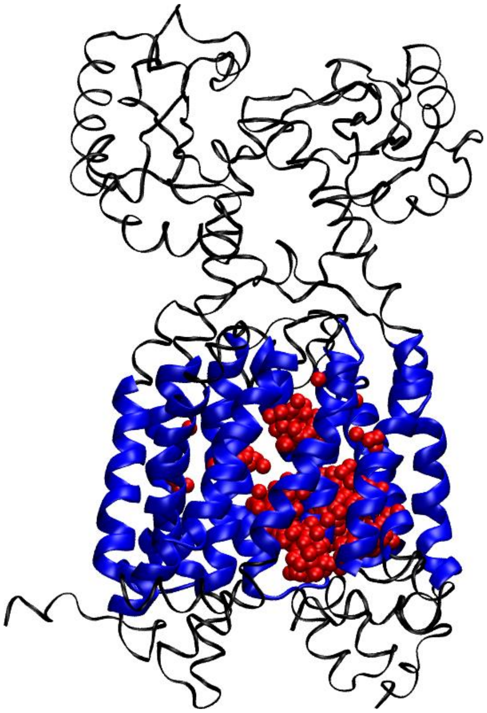

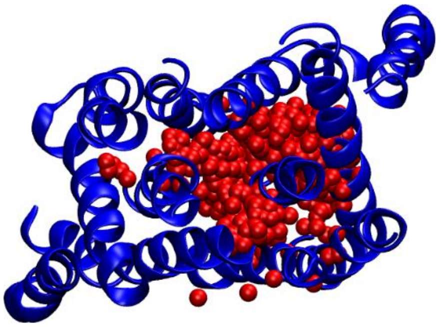

(C) Intracellular

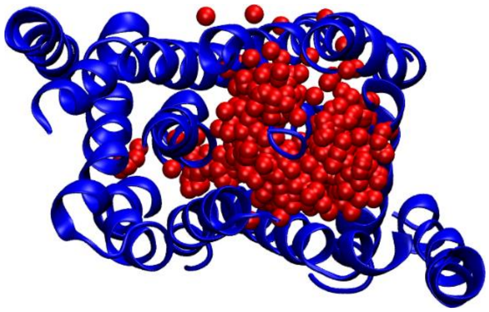

(D) Front	(E) Extracellular

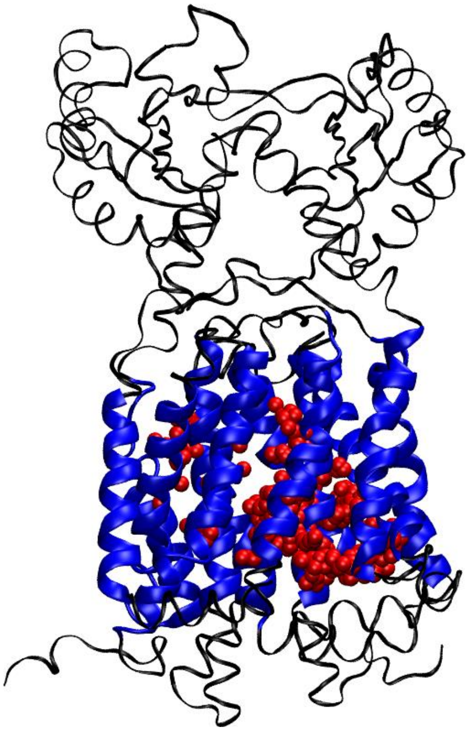

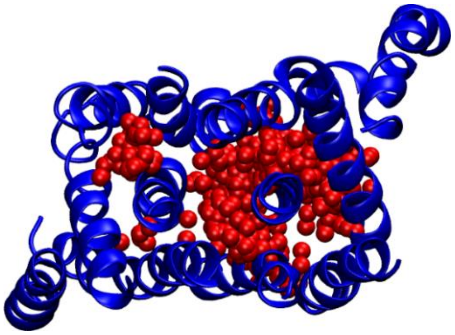

(F) Intracellular

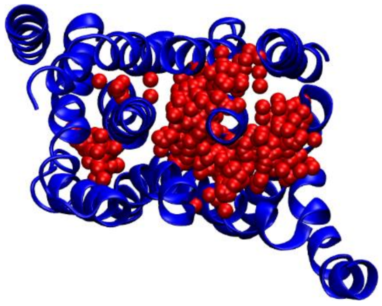

(G) Front	(H) Extracellular

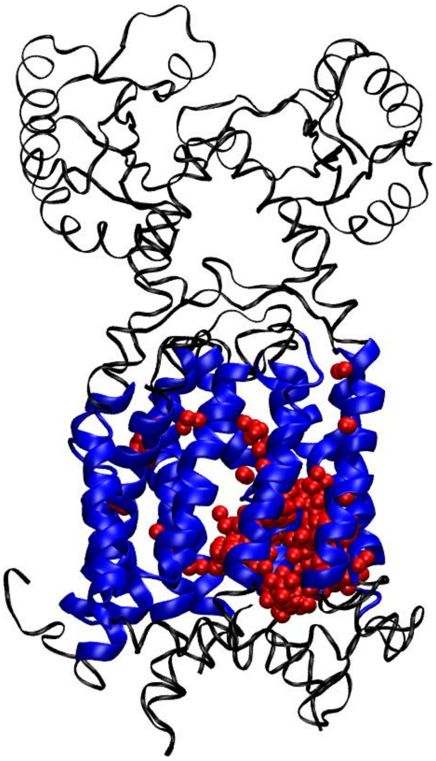

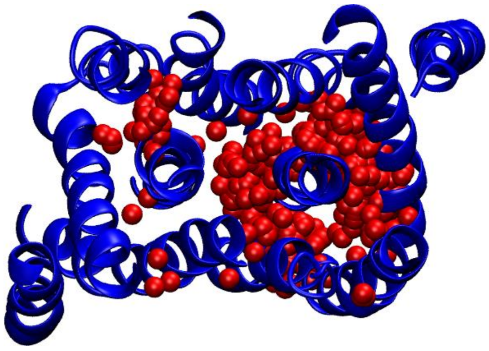

(I) Intracellular

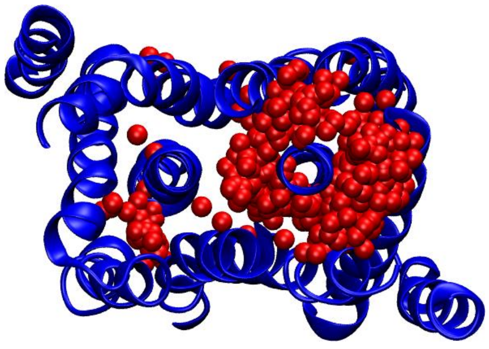

Figure S32. Distribution of all water molecules (red) within the transmembrane throughout entire trajectories of the apo-form from (**A, B, C**) simulation one, (**D, E, F**) two, and (**G, H, I**) three.

**(A) Front**	**(B) Top**
	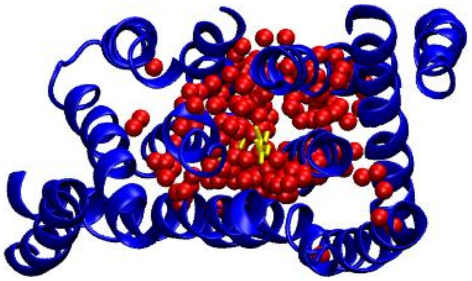
	**(C) Bottom**
	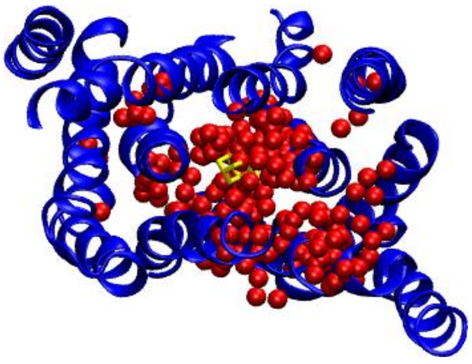

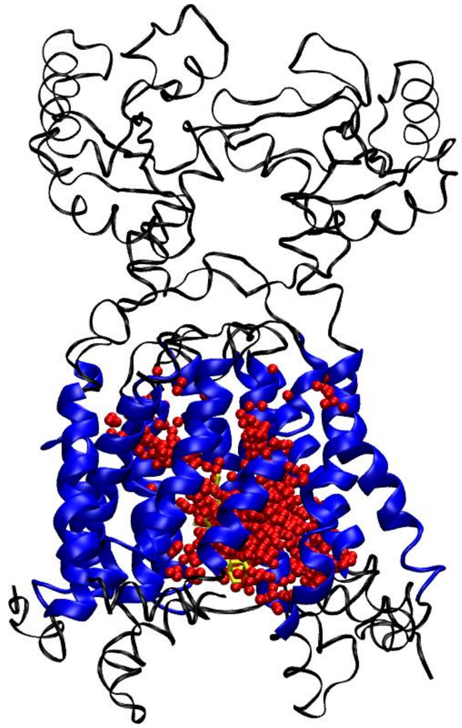

(D) Front

(E) Top

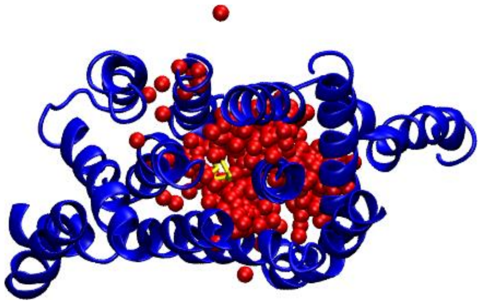

(F) Bottom

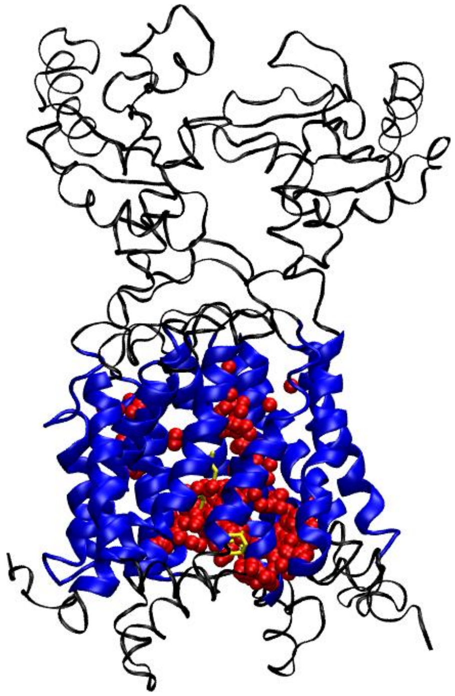

(G) Front

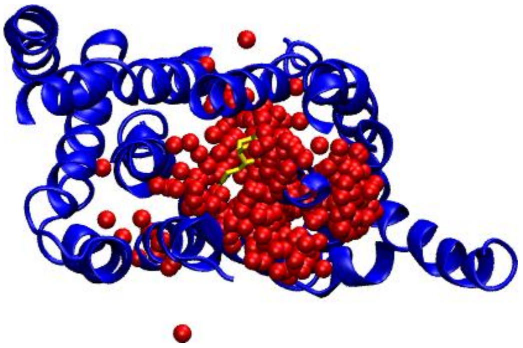

(H) Top

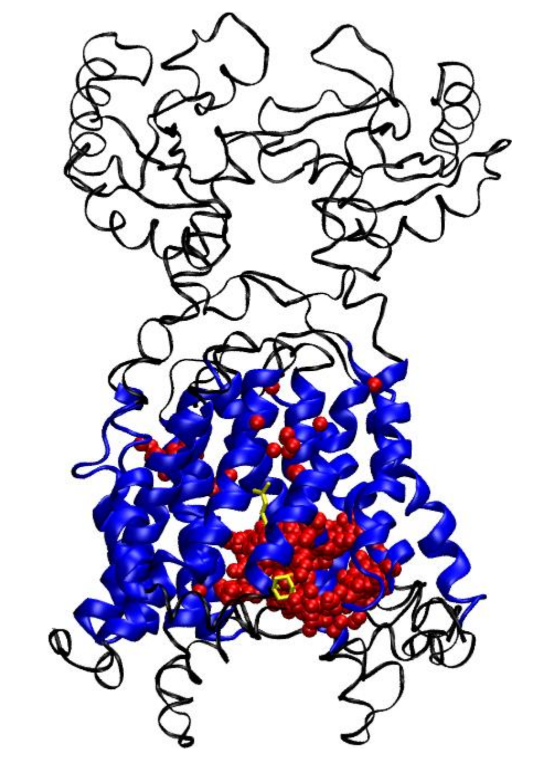

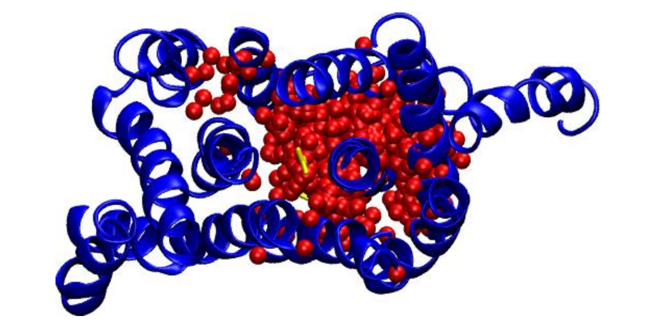

(I) Bottom

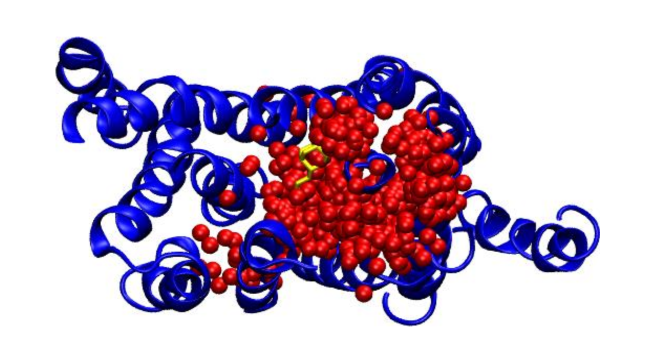

Figure S33. Distribution of all water molecules (red) within the transmembrane throughout entire trajectories of the holo-form from (**A, B, C**) simulation one, (**D, E, F**) two, and (**G, H, I**) three.

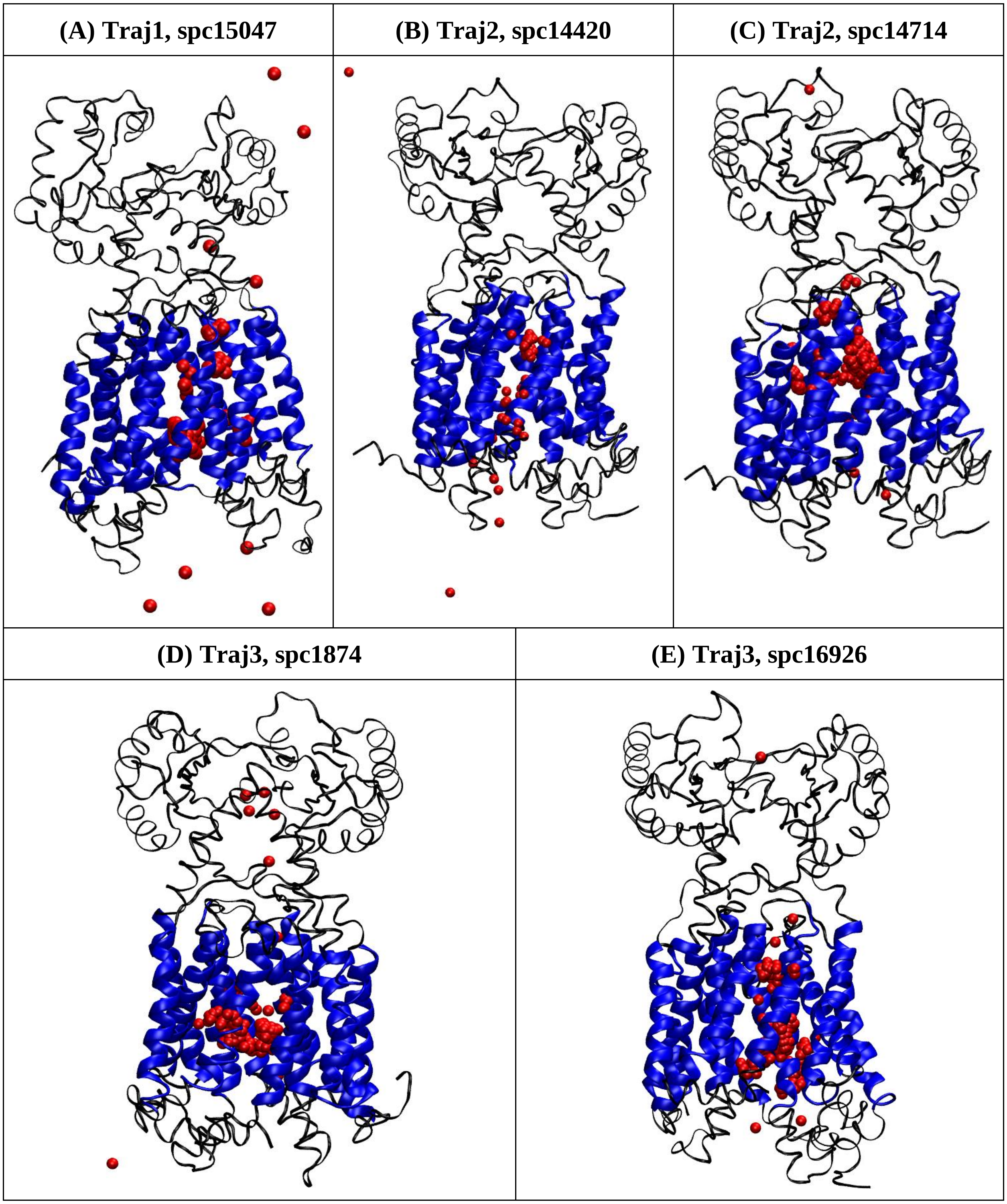

Figure S34. Representative water molecules (red) passing from the extracellular space, through MmpL3 channel and exiting out into the intracellular space from the apo-form trajectories.

(A)

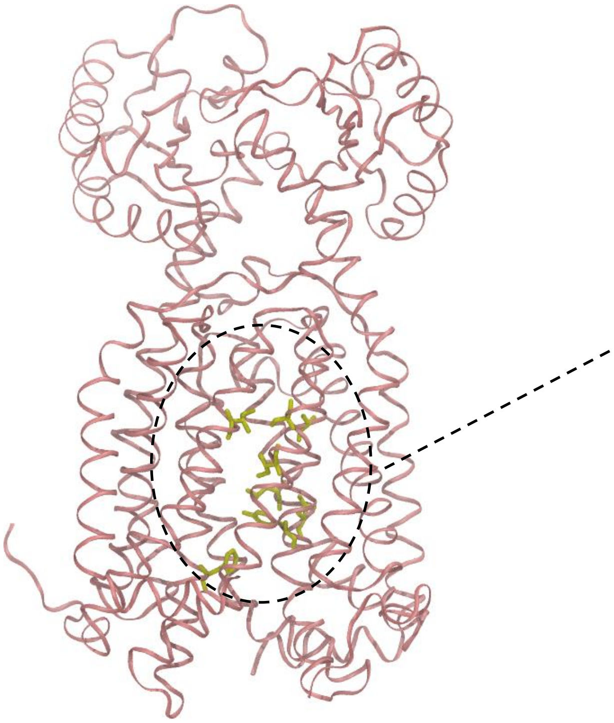

(B)

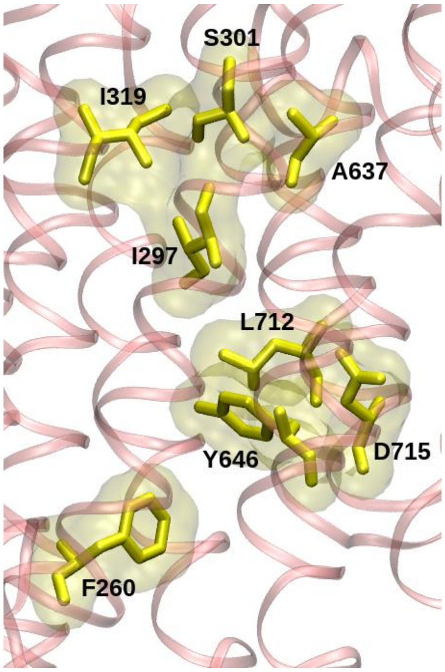

(C)

(D)

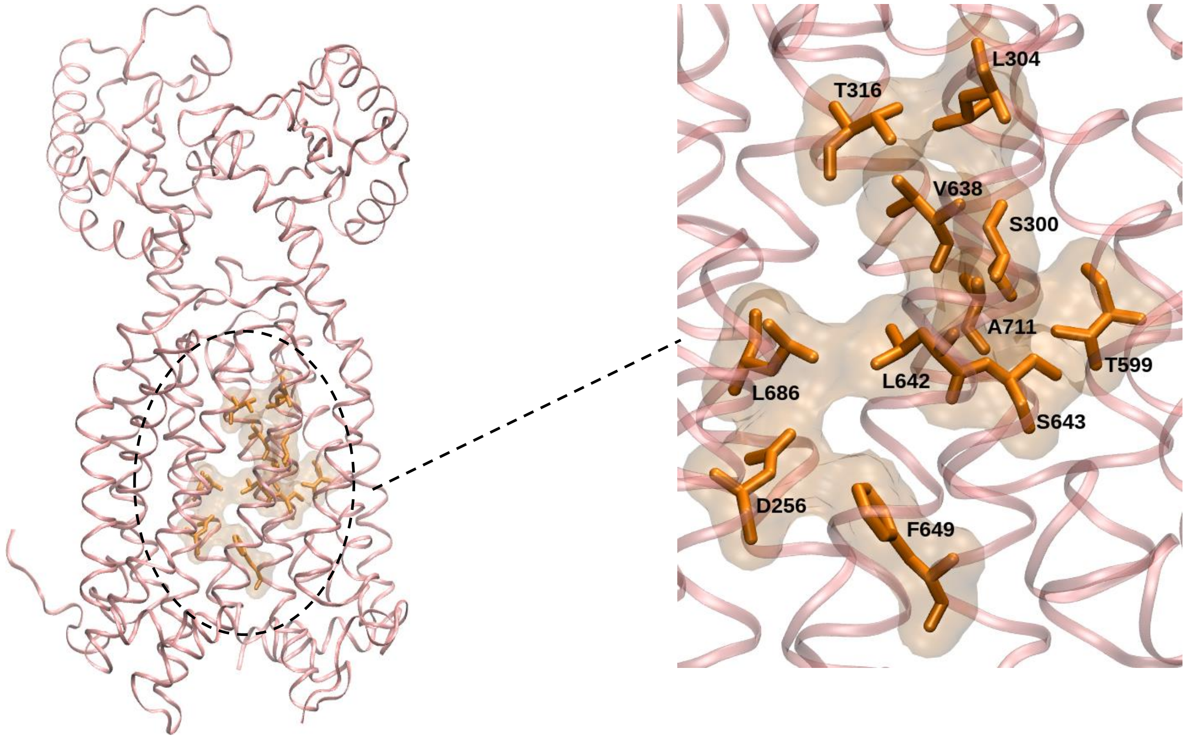

(E)

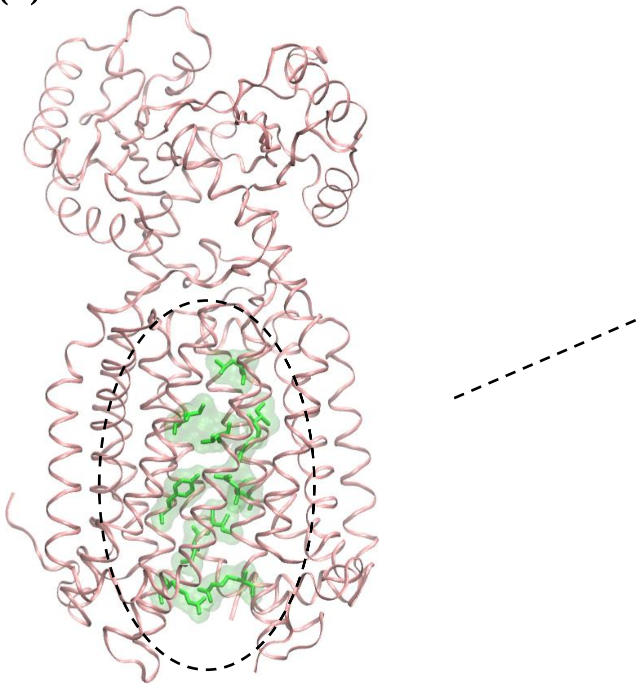

(F)

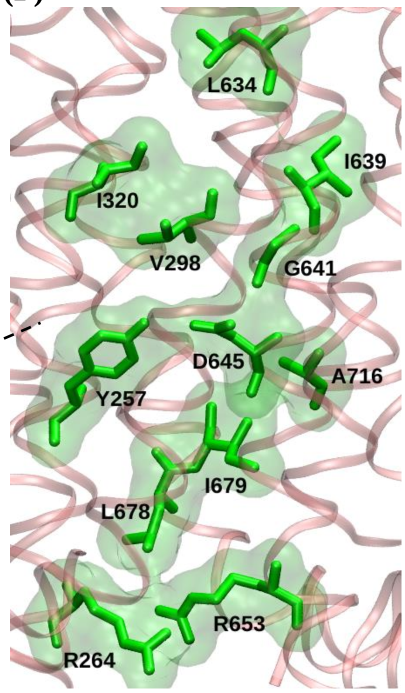

Figure S35. MmpL3 water-binding residues that occurred in all all five (**A & B**), four (**C & D**) or three (**E & F**) of the water passage events. See **Tables S3-S7**.

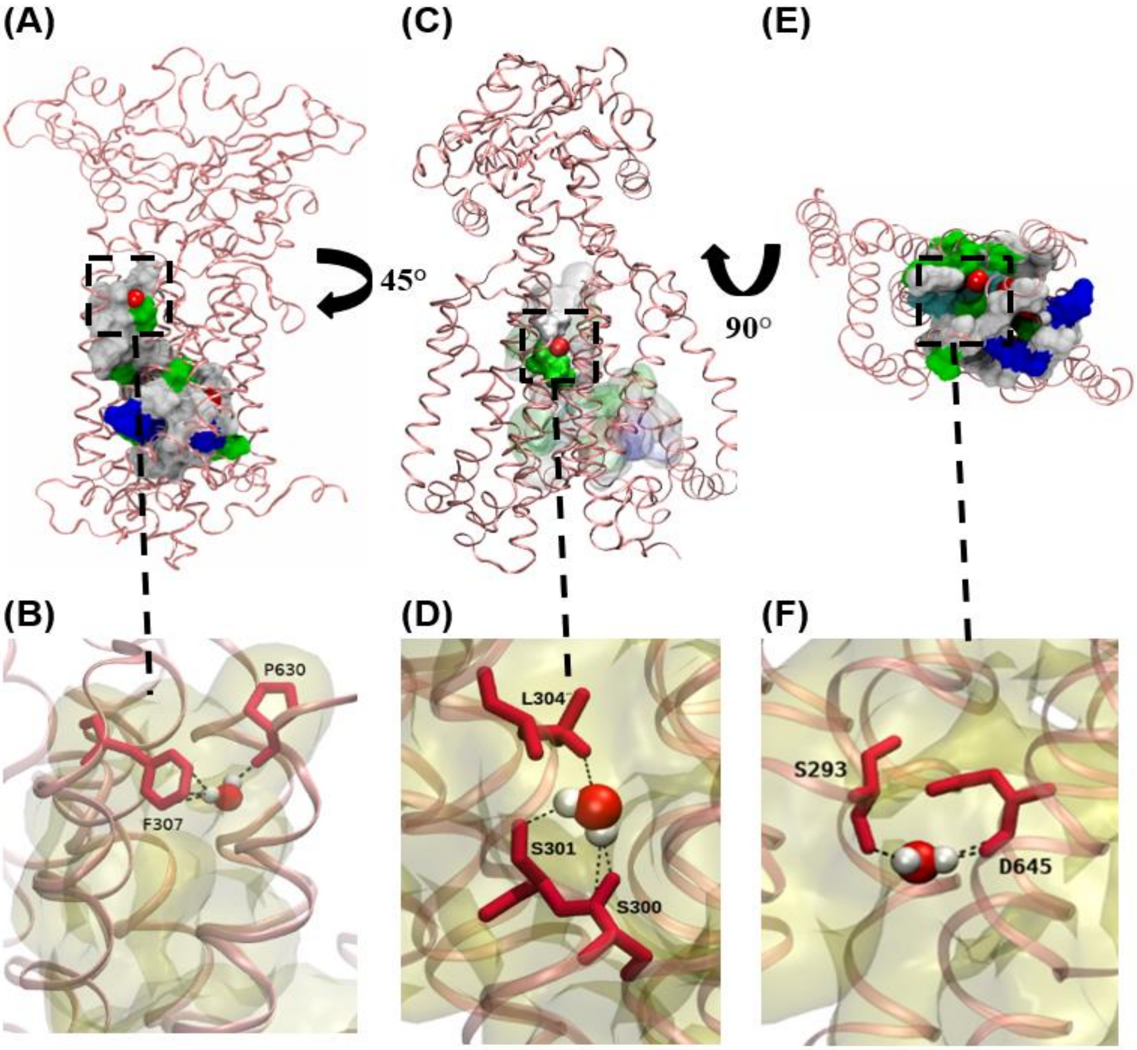

Figure S36. (**A**) Water-binding residues throughout simulation **1** of the apo form (PDB ID: 6AJF) to wat species 15047 from **(B & C)** the point of entry to (**D & E**) the point of exit. Residues are colored as acidi (red), basic (blue), polar (green), and non-polar (grey).

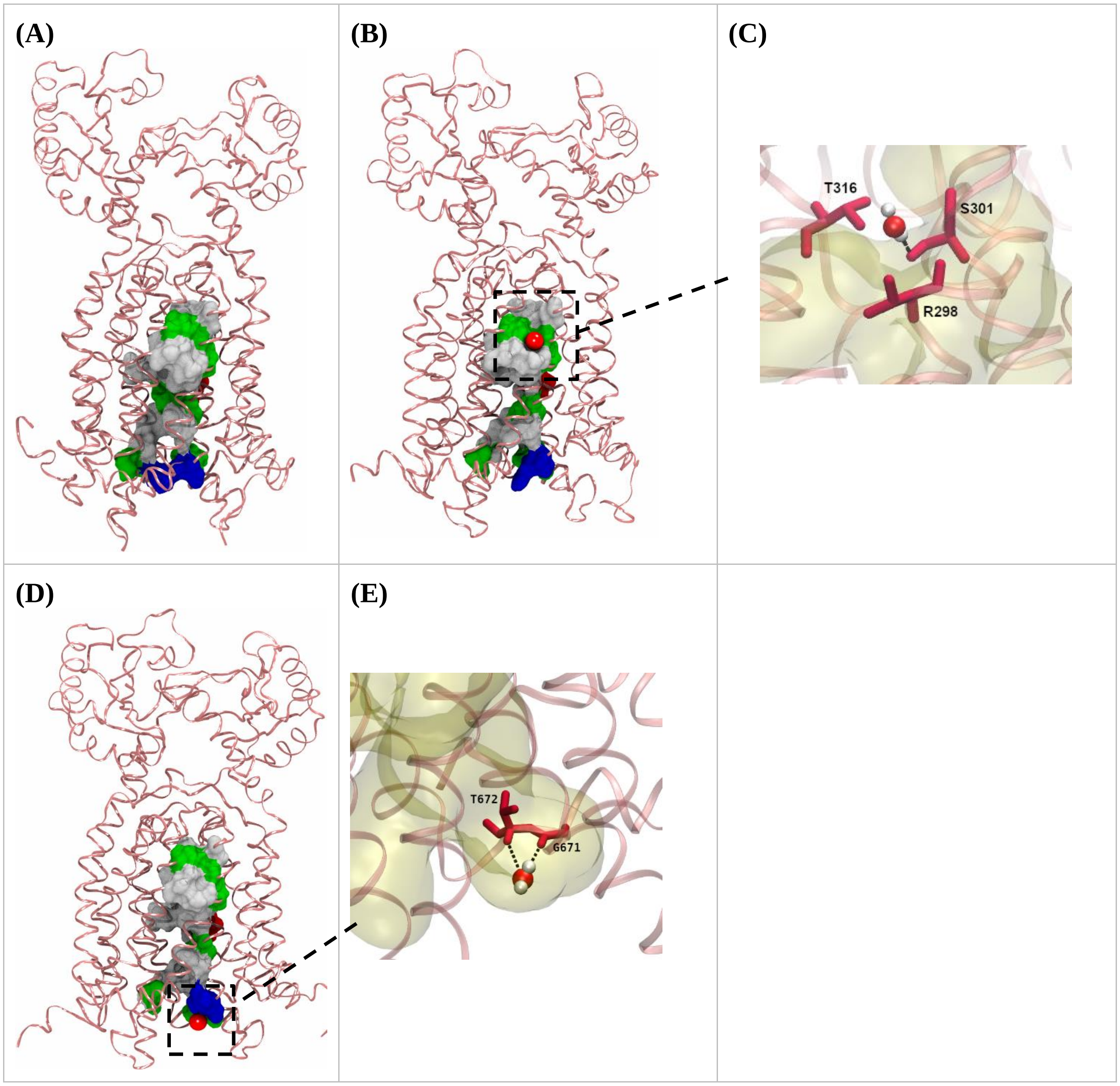

Figure S37. (**A**) Water-binding residues throughout simulation 2 of the apo-form (PDB ID: 6AJF) of water species 14420 from (**B & C**) the point of entry to (**D & E**) the point of exit. Residues are colored as (red), basic (blue), polar (green), and non-polar (grey).

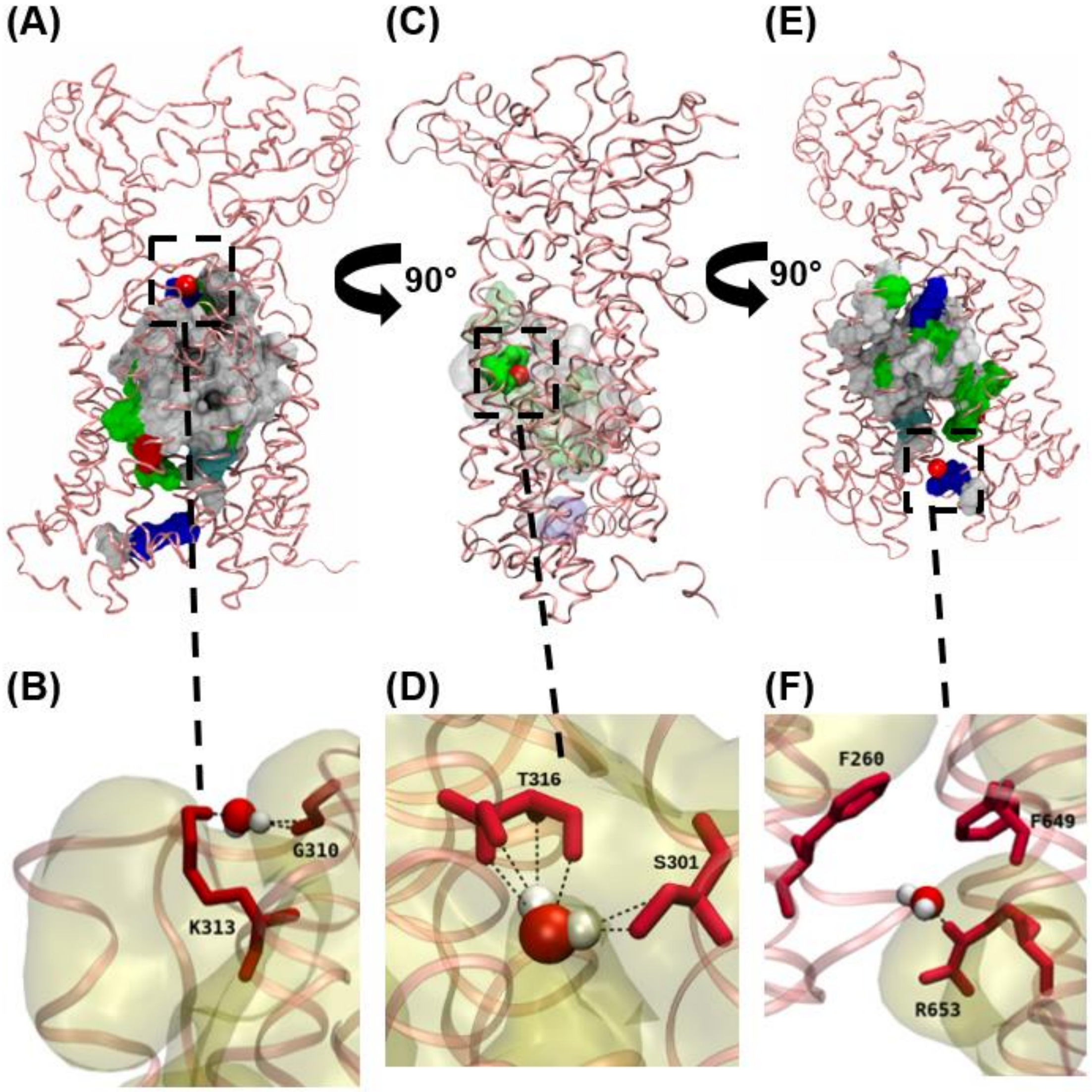

Figure S38. (**A**) Water-binding residues throughout simulation 2 of the apo form (PDB ID: 6AJF) of water species 14714 from (**B & C**) the point of entry to (**D & E**) the point of exit. Residues are colored as acidic (red), basic (blue), polar (green), and non-polar (grey).

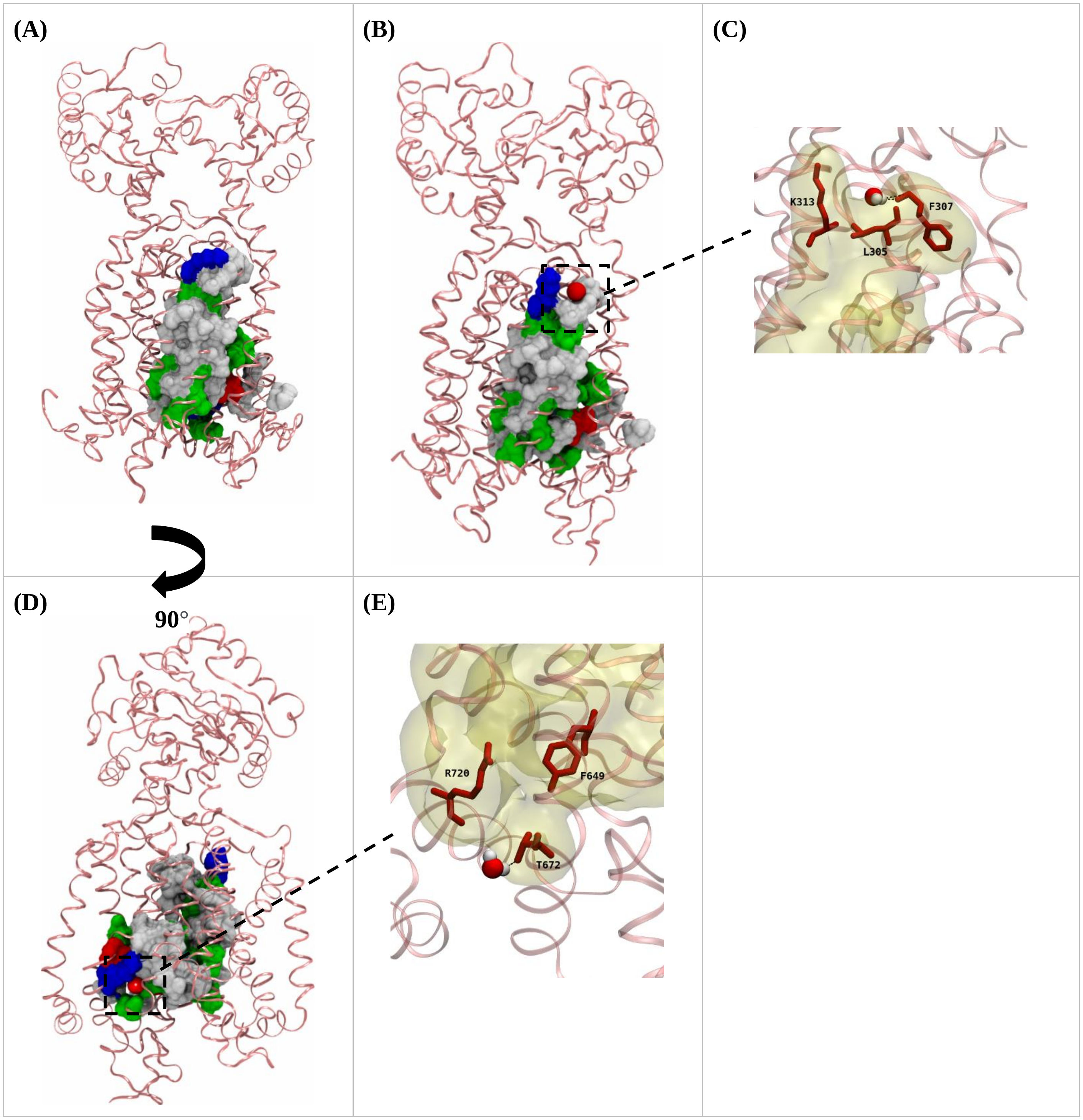

Figure S39. (**A**) Water-binding residues throughout simulation 3 of the apo-form (PDB ID: 6AJF) of water species 16926 from (**B & C**) the point of entry to (**D & E**) the point of exit. Residues are colored as (red), basic (blue), polar (green), and non-polar (grey).

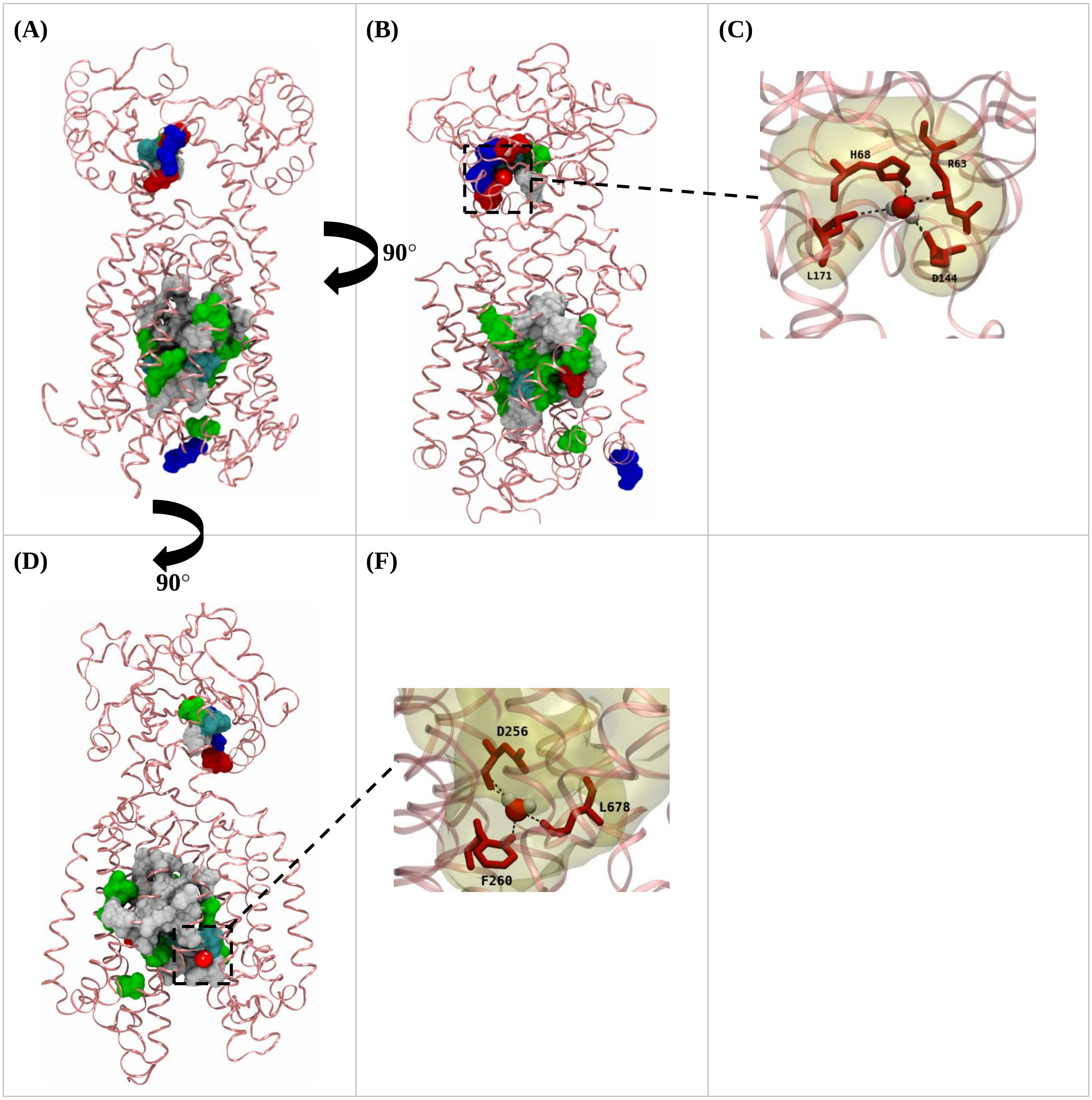

Figure S40. (**A**) Water-binding residues throughout simulation 3 of the apo-form (PDB ID: 6AJF) of water species 1874 from (**B & C**) the point of entry to (**D & E**) the point of exit. Residues are colored as (red), basic (blue), polar (green), and non-polar (grey).

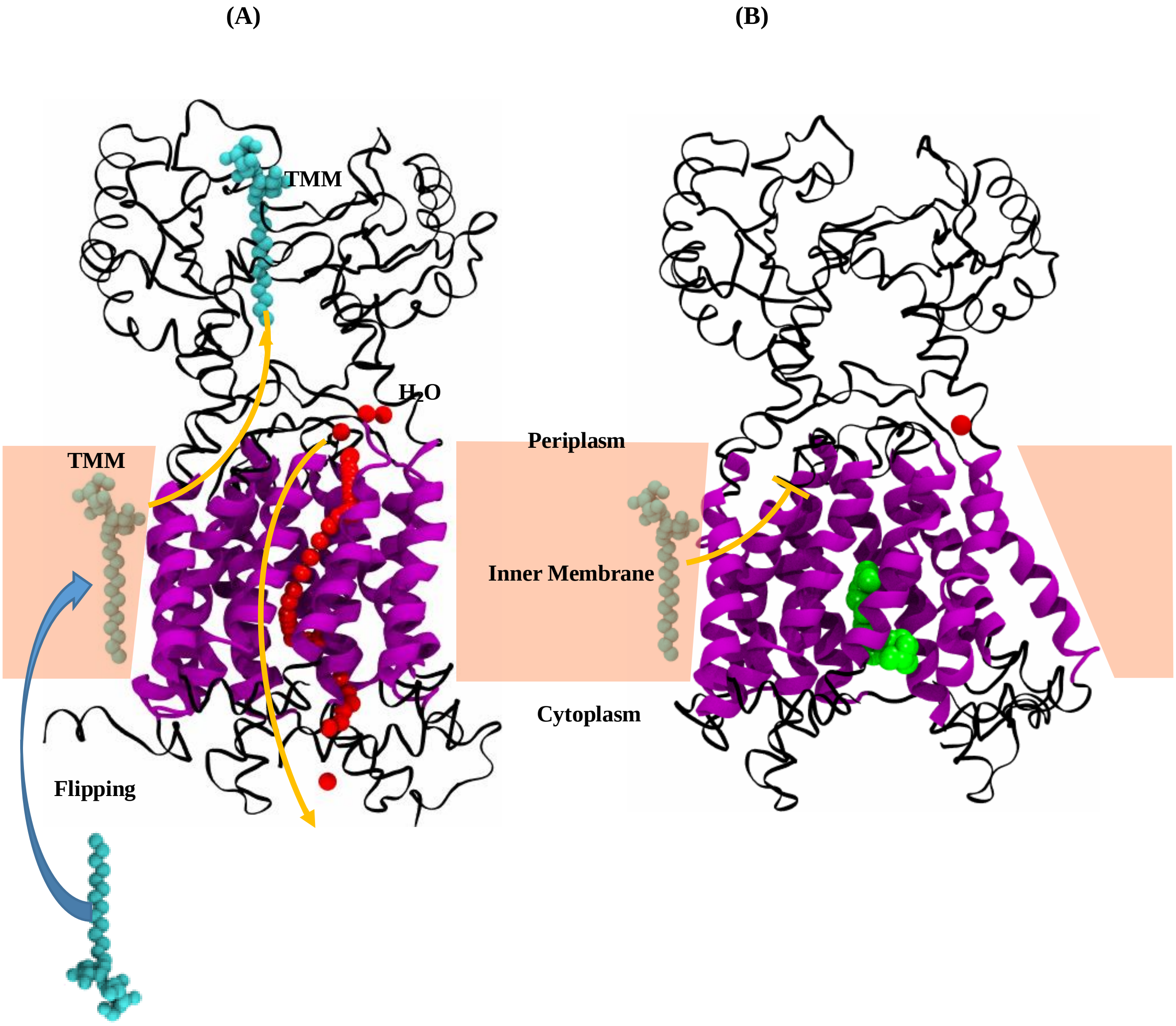

Figure S41. Proposed mechanism of (**A**) proton translocation in the apo form MmpL3 and (**B**) proton translocation inhibition via SQ109.
